# Supplementary material for: Quantitative Checklist for Autism in Toddlers (Q-CHAT). A population screening study with follow-up: the case for multiple time-point screening for autism
Source: BMJ Paediatr Open. 2021 May 28;5(1):e000700. doi: 10.1136/bmjpo-2020-000700 (PMC8166626; doi:10.1136/bmjpo-2020-000700)
Supplement: Supplementary data [file bmjpo-2020-000700supp001.pdf]

Supplementary Appendix

Supplementary methods

Sensitivity analyses

Analysis was undertaken to maximise the sensitivity and specificity using a range of cut points across the Q-CHAT distribution. Additional cut points that also maximised sensitivity and specificity but had a larger number of false positives are provided. In addition a-priori a measure of parental concern taken at the same time as the screening test was included to investigate whether it improved the screening properties. Additionally, an analysis for initial non-response was undertaken to show the results were robust to the initial missing data.

Supplementary Table 1: Study Characteristics

		Phase I				Phase II			
		Total		Assessment		Total		Assessment	
PCT	Luton	416	(11%)	22	(18%)	176	(9%)	19	(12%)
	Bedfordshire	1217	(33%)	39	(32%)	638	(32%)	55	(35%)
	Cambridgeshire	2018	(55%)	60	(50%)	1183	(59%)	84	(53%)
Child sex	Female	1759	(48%)	42	(35%)	972	(49%)	56	(35%)
	Male	1884	(52%)	79	(65%)	1025	(51%)	102	(65%)
	Unknown*	70		0		1		0	
Age child at Phase I	16–19 months	251	(7%)	15	(12%)	142	(7%)	13	(8%)
	20–24 months	1478	(41%)	49	(40%)	831	(42%)	69	(44%)
	25–29 months	1474	(40%)	46	(38%)	814	(41%)	66	(42%)
	30–34 months	440	(12%)	11	(9%)	210	(11%)	10	(6%)
	Unknown*	125		0		6		0	

* Children with unknown sex or age were excluded from the analysis and not included within the rest of the table

+ General Certificate of Secondary Education

		Phase I				Phase II			
		**Total**		**Assessment**		**Total**		**Assessment**	
Mothers age at birth (Median, IQR)		32	[28,35]	31	[27,36]	33	[29,36]	32	[29,36]
Mothers education	None / <5 GCSE+	480	(13%)	25	(21%)	166	(8%)	19	(12%)
	5 GCSE	548	(15%)	21	(17%)	252	(13%)	24	(15%)
	2+ A levels	512	(14%)	11	(9%)	255	(13%)	15	(9%)
	Degree	1684	(45%)	48	(40%)	1068	(53%)	80	(51%)
	Unknown	546	(14%)	16	(13%)	267	(13%)	20	(13%)
Mother occupation	Not working	1545	(41%)	58	(48%)	736	(37%)	67	(42%)
	Student	86	(2%)	4	(3%)	40	(2%)	5	(3%)
	Employed	1044	(28%)	28	(23%)	592	(29%)	43	(27%)
	Self employed no employees	176	(5%)	5	(4%)	105	(5%)	9	(6%)
	Self employed with employees, employed supervisor	835	(22%)	22	(18%)	513	(26%)	31	(20%)
	Unknown	84	(2%)	4	(3%)	22	(1%)	3	(2%)
Mother	White	3236	(86%)	94	(78%)	1795	(89%)	141	(89%)
	Black (African or Caribbean)	103	(3%)	6	(5%)	36	(2%)	4	(3%)
	South East Asian	231	(5%)	13	(11%)	78	(4%)	7	(4%)
	Other / Unknown	200	(5%)	8	(7%)	99	(5%)	6	(4%)

		Phase I				Phase II			
		**Total**		**Assessment**		**Total**		**Assessment**	
Fathers age at birth (Median, IQR)		34	[31,38]	34	[31,37]	35	[31,39]	35	[31,39]
Father education	None / <5 GCSE	613	(16%)	22	(18%)	268	(13%)	23	(15%)
	5 GCSE	477	(13%)	11	(9%)	223	(11%)	14	(9%)
	2+ A levels	439	(12%)	14	(12%)	232	(12%)	22	(14%)
	Degree	1547	(41%)	41	(34%)	983	(49%)	62	(39%)
	Unknown	694	(18%)	33	(27%)	302	(15%)	37	(23%)
Father occupation	Not working	166	(4%)	10	(8%)	61	(3%)	5	(3%)
	Student	40	(1%)	1	(1%)	14	(1%)	1	(1%)
	Employed	1153	(31%)	38	(31%)	634	(32%)	51	(32%)
	Self–employed – no employees	351	(9%)	11	(9%)	196	(10%)	20	(13%)
	Self–employed – with employees, employed supervisor	1729	(46%)	41	(34%)	994	(49%)	66	(42%)
	Unknown	331	(9%)	20	(17%)	109	(5%)	15	(9%)
Father ethnicity	White	3171	(84%)	96	(79%)	1780	(87%)	138	(87%)
	Black (African or Caribbean)	142	(4%)	8	(7%)	52	(3%)	6	(4%)
	South East Asian	237	(6%)	10	(8%)	78	(4%)	6	(4%)
	Other / Unknown	220	(6%)	7	(6%)	98	(5%)	8	(5%)

Supplementary Figure 1: Phase 1 study flow

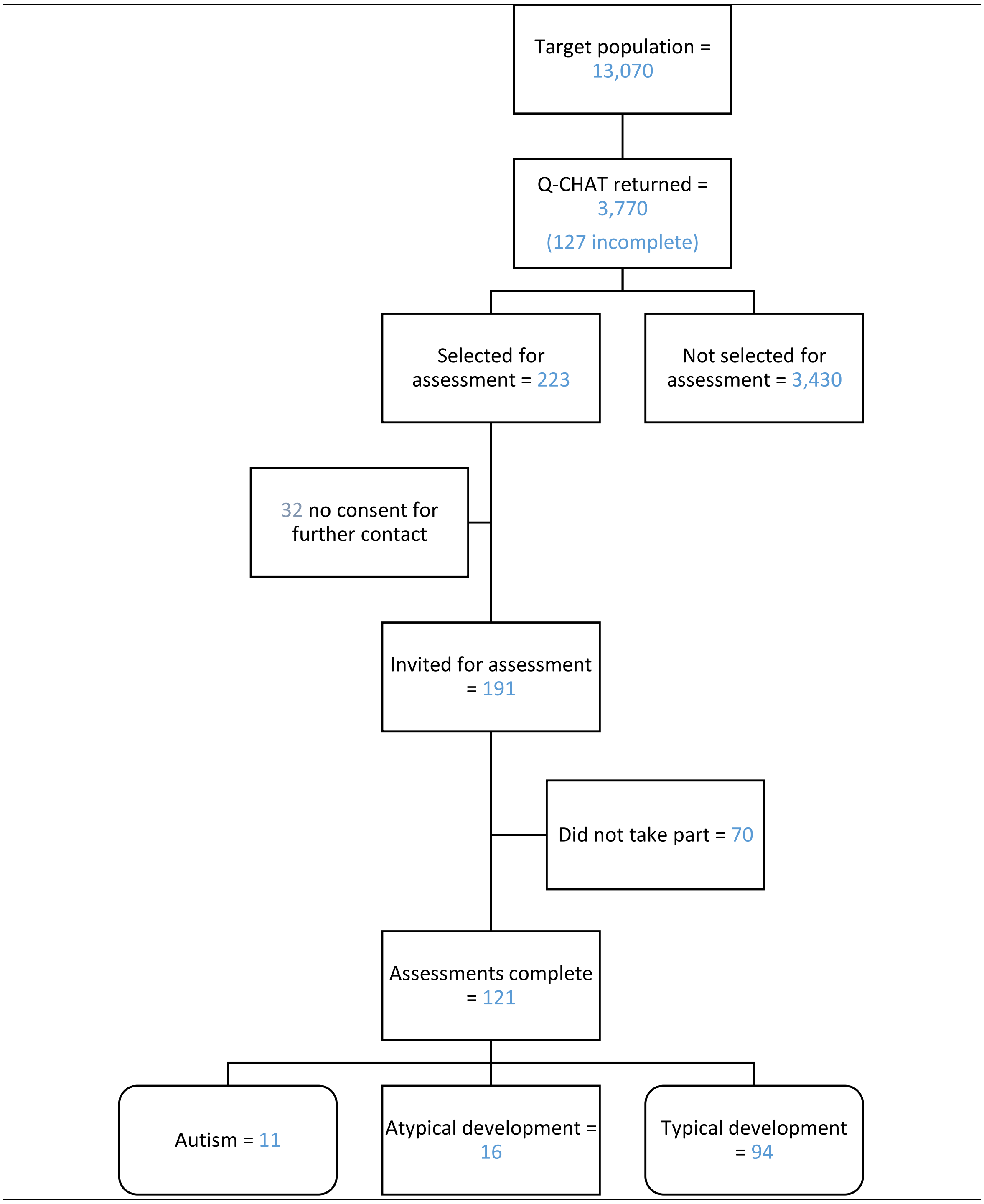

Allison C, *et al. BMJ Paediatrics Open* 2021; 5:e000700. doi: 10.1136/bmjpo-2020-000700

Supplementary Figure 2: Distribution of Q–CHAT at first interview, with marks for screening groups

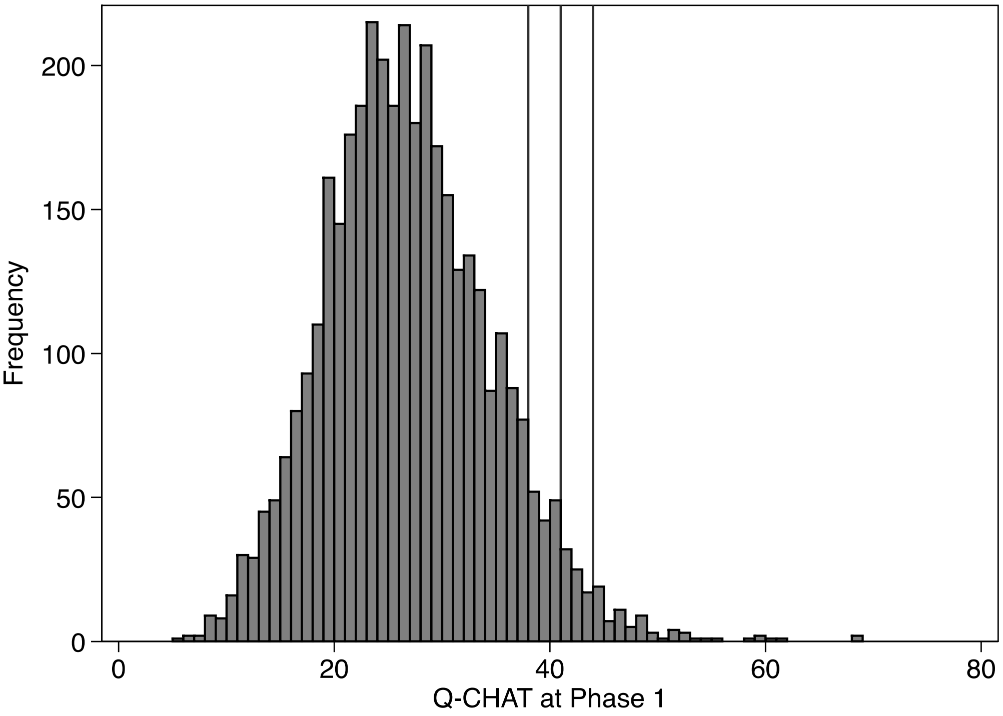

Allison C, *et al. BMJ Paediatrics Open* 2021; 5:e000700. doi: 10.1136/bmjpo-2020-000700

Supplementary Figure 3: ROC curves for Q–CHAT and CAST on Phase 1 and Phase 2 outcomes for autism

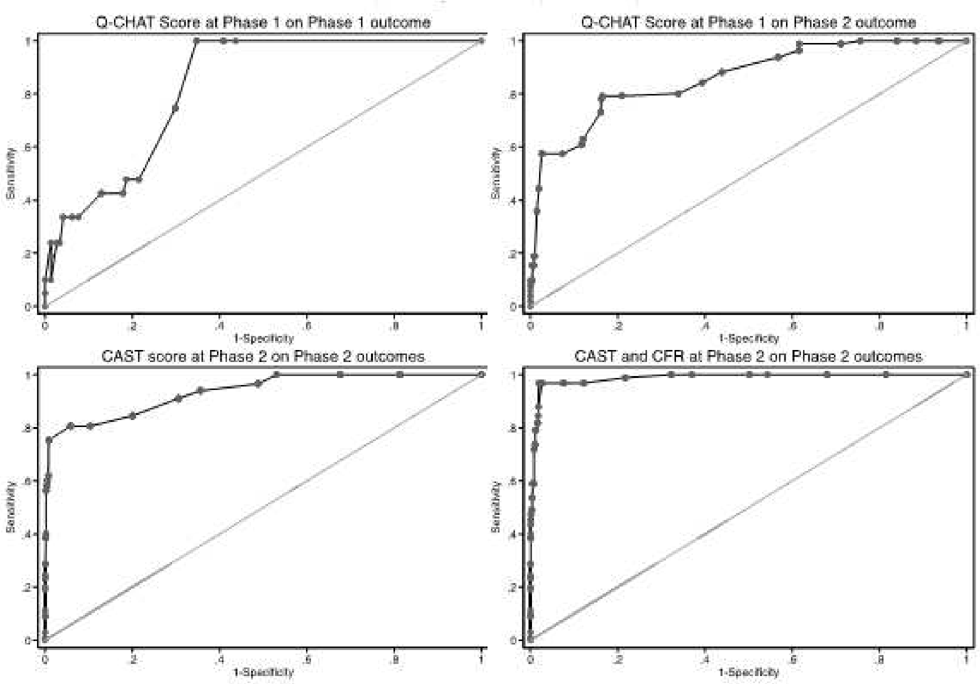

Supplementary Figure 4: Box-plot of the distribution of Q-CHAT scores split by both Phase 1 and Phase 2 diagnosis

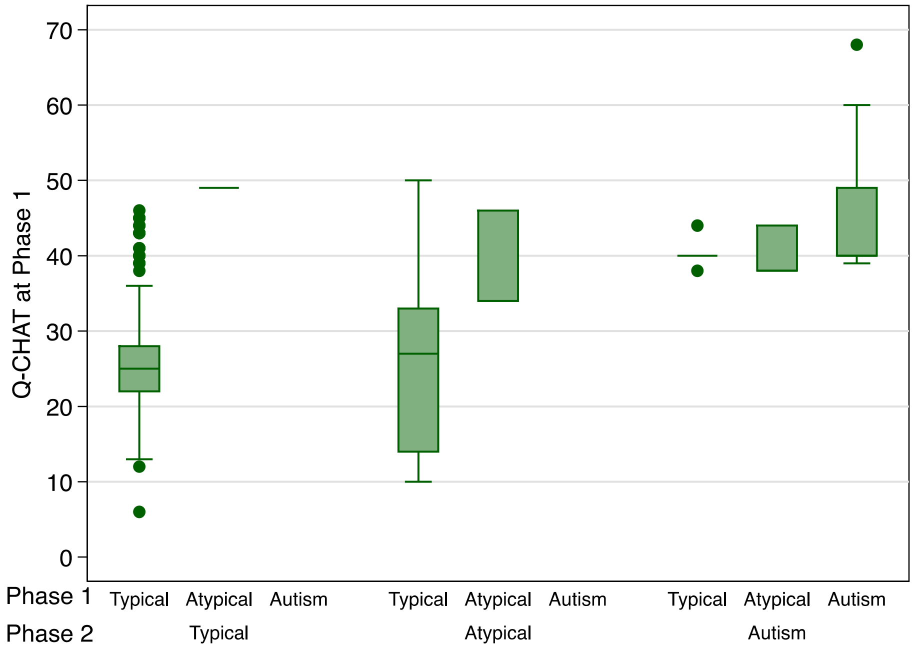

Allison C, *et al. BMJ Paediatrics Open* 2021; 5:e000700. doi: 10.1136/bmjpo-2020-000700
