# Supplementary material for: Quantitative Checklist for Autism in Toddlers (Q-CHAT). A population screening study with follow-up: the case for multiple time-point screening for autism
Source: BMJ Paediatr Open. 2021 May 28;5(1):e000700. doi: 10.1136/bmjpo-2020-000700 (PMC8166626; doi:10.1136/bmjpo-2020-000700)
Supplement: Author's manuscript [file bmjpo-2020-000700.draft_revisions.pdf]

BMJ Paediatrics Open

BMJ Paediatrics Open is committed to open peer review. As part of this commitment we make the peer review history of every article we publish publicly available.

When an article is published we post the peer reviewers’ comments and the authors’ responses online. We also post the versions of the paper that were used during peer review. These are the versions that the peer review comments apply to.

The versions of the paper that follow are the versions that were submitted during the peer review process. They are not the versions of record or the final published versions. They should not be cited or distributed as the published version of this manuscript.

BMJ Paediatrics Open is an open access journal and the full, final, typeset and author-corrected version of record of the manuscript is available on our site with no access controls, subscription charges or pay-per-view fees (http://bmjpaedsopen.bmj.com).

If you have any questions on BMJ Paediatrics Open’s open peer review process please email info.bmjpo@bmj.com

BMJ Paediatrics Open

The Quantitative Checklist for Autism in Toddlers (Q–CHAT). A population screening study with follow-up: the case for multiple time–point screening for autism.

Journal:	*BMJ Paediatrics Open*
Manuscript ID	bmjpo-2020-000700
Article Type:	Original research
Date Submitted by the Author:	18-Sep-2020

Complete List of Authors:	Allison, Carrie; Autism Research Centre, Psychiatry Department<br>Matthews, Fiona; Newcastle University, Institute for Health and Society, Biomedical Research Building, Campus for Ageing and Vitality<br>Ruta, Liliana; National Research Council, Institute of Applied Sciences and Intelligent Systems, "Eduardo Caianiello" (ScienceApp)<br>Pasco, Greg; Institute of Psychiatry Psychology and Neuroscience, Psychology<br>Soufer, Renee; Autism Research Centre, Psychiatry Department<br>Brayne, Carol; University of Cambridge, Institute of Public Health<br>Charman, Tony; King's College London, Institute of Psychiatry, Department of Psychology<br>Baron-Cohen, Simon; Autism Research Centre, Psychiatry Department

Keywords:	Autism, Screening

SCHOLARONE™
Manuscripts

I, the Submitting Author has the right to grant and does grant on behalf of all authors of the Work (as defined in the below author licence), an exclusive licence and/or a non-exclusive licence for contributions from authors who are: i) UK Crown employees; ii) where BMJ has agreed a CC-BY licence shall apply, and/or iii) in accordance with the terms applicable for US Federal Government officers or employees acting as part of their official duties; on a worldwide, perpetual, irrevocable, royalty-free basis to BMJ Publishing Group Ltd ("BMJ") its licensees and where the relevant Journal is co-owned by BMJ to the co-owners of the Journal, to publish the Work in this journal and any other BMJ products and to exploit all rights, as set out in our licence.

The Submitting Author accepts and understands that any supply made under these terms is made by BMJ to the Submitting Author unless you are acting as an employee on behalf of your employer or a postgraduate student of an affiliated institution which is paying any applicable article publishing charge ("APC") for Open Access articles. Where the Submitting Author wishes to make the Work available on an Open Access basis (and intends to pay the relevant APC), the terms of reuse of such Open Access shall be governed by a Creative Commons licence – details of these licences and which Creative Commons licence will apply to this Work are set out in our licence referred to above.

Other than as permitted in any relevant BMJ Author's Self Archiving Policies, I confirm this Work has not been accepted for publication elsewhere, is not being considered for publication elsewhere and does not duplicate material already published. I confirm all authors consent to publication of this Work and authorise the granting of this licence.

Confidential: For Review Only

The Quantitative Checklist for Autism in Toddlers (Q–CHAT). A population screening study with follow-up: the case for multiple time–point screening for autism.

Confidential: For Review Only

Allison, C.*[1], Matthews, F.E. [2], Ruta, L.[1,3], Pasco, G.[4], Soufer, R.[1], Brayne, C.[5], Charman, T.[4], & Baron–Cohen, S. [1]

[1] Autism Research Centre, University of Cambridge, Department of Psychiatry, Douglas House, 18B Trumpington Road, Cambridge, CB2 8AH, UK

[2] Institute of Health and Society, Campus for Ageing and Vitality, Newcastle University, Newcastle upon Tyne, NE4 5PL, UK

[3] Institute of Applied Sciences and Intelligent Systems, "Eduardo Caianiello" (ScienceApp), National Research Council of Italy (CNR), Messina, Italy.

[4] Department of Psychology, Institute of Psychiatry, Psychology & Neuroscience, King's College London, DeCrespigny Park, Denmark Hill, London, SE5 8AF, UK

[5] Department of Public Health and Primary Care, Cambridge Institute of Public Health, University of Cambridge, CB2 0SR

* Corresponding author cla29@cam.ac.uk tel 01223 465233

Objectives: Prospective population screening study for autism in 18-30-month-old toddlers using the Quantitative Checklist for Autism in Toddlers (Q-CHAT), with follow-up at age 4 years.

Design: Observational Study.

Setting: Luton and Bedfordshire, and Cambridgeshire in the United Kingdom.

Participants: 13,070 toddlers registered on the Child Health Surveillance Database between March 2008 and April 2009, with follow–up at age 4; 3,770 (29%) were screened for autism at 18–30 months using the Q–CHAT and the Childhood Autism Screening Test (CAST) at follow up at age 4 years.

Interventions: A stratified sample across the Q-CHAT score distribution was invited for diagnostic assessment (Phase 1). The 4 year follow–up included the Childhood Autism Screening Test (CAST) and Checklist for Referral (CFR). All with CAST ≥15, Phase 1 diagnostic assessment, or, with developmental concerns on the CFR were invited for diagnostic assessment (Phase 2). Standardised diagnostic assessment at both time-points was conducted to establish the test accuracy of the Q-CHAT.

Main outcome measures: Consensus diagnostic outcome at Phase 1 and Phase 2.

Results: Phase 1: 3,770 Q–CHATs were returned (29% response), 121 undertook diagnostic assessment, of whom 11 met criteria for autism. All 11 screened positive on the Q–CHAT. The positive predictive value (PPV) at a cut–point of 39 was 17% (95%CI 8%–31%). Phase 2: 2,005/3,472 CASTs were returned (58% response). 159 underwent diagnostic assessment, including 82 assessed in Phase 1. All children meeting criteria for autism were identified via the Q–CHAT at Phase 1 also met criteria at Phase 2. PPV was 28% (95%CI 15%–46%).

Conclusions: The Q–CHAT can be used at 18–30 months to identify autism and enable accelerated referral for diagnostic assessment. For every true positive there would, however, be 5 false positives. At follow–up, new cases were identified illustrating the need for continued surveillance and re–screening at multiple time–points using developmentally sensitive instruments. Not all children who later receive a diagnosis are detectable during the toddler period.

Confidential: For Review Only

Research in Context

What is already known on this topic

In the UK, universal screening for autism is not recommended to facilitate early detection. There is limited research evidence of specific autism screening tools with longitudinal follow-up.

What this study adds

We examined the accuracy of the Quantitative Checklist for Autism in Toddlers (Q-CHAT) in a population cohort screened at 18-30 month and followed-up at age 4 years. The sensitivity and positive predictive value of the Q-CHAT were marginally higher than other screening tools, which supports other studies that have found it is not possible to identify all autistic children using a single instrument at a single time–point.

Confidential: For Review Only

Introduction

Autism affects approximately 1–2% of the population.[1] Screening requires a standardised and systematic approach to detect children who may require a diagnostic assessment. The costs and benefits of autism population screening need to be carefully balanced. Undue anxiety in false positives and the need for sufficient support services for those diagnosed should be offset by the many benefits of accurate screening that include earlier diagnosis, more timely access to specific interventions[2] and, better support for parents. In the UK, no autism specific screening instrument is recommended by the National Institute of Health and Care Excellence (NICE).[3] Autism is variable in onset and the significance over time of specific symptoms. No existing screening instrument, used at a single time–point, reaches a minimum of 80% sensitivity and specificity. In contrast, the American Academy of Pediatrics[4] recommends routine screening at well–child checks using the Checklist for Autism in Toddlers (CHAT)[5, 6] and the Modified CHAT (M–CHAT),[7] despite the limited research evidence for their use,[8] and insufficient evidence to determine if certain risk factors modify the performance characteristics of the tests.[9]

Lengthy delays between first concerns and an eventual diagnosis are often experienced.[10] There has been no evidence of a reduction in the age at diagnosis over the past decade in the UK.[11] Beliefs held by health professionals about screening for autism (e.g. system capacity, interventions available) may influence practice.[12] Equally, young autistic children may not present salient atypical behaviour in a short consultation to warrant an assessment referral. Atypical behaviours, such as Restricted and Repetitive Behaviours (RRBs) extend to the neurotypical child on a continuum[13] and spontaneously reduce with time.[14] The reduction of inappropriate referrals to already over–stretched services could serve to benefit children, their families and health services. 60% of referrals to a child development centre in the UK for possible autism resulted in an autism diagnosis. It is unclear what the outcome was for the remainder of the referrals.[15]

The CHAT[5] is a short parent–report questionnaire combined with a health professional observation. The CHAT was tested prospectively in 18 month old toddlers from the general population with diagnostic follow–up of those who scored above the cut–point to allow for the estimation of both sensitivity and specificity. At 18 months the CHAT identified 92% of autistic children when assessed and diagnosed by two independent psychiatrists using DSM–III–R criteria as the gold standard at that time.[6] By the 6–year follow–up, the sensitivity was

Confidential: For Review Only

38% indicating that many children did not present with sufficient features of autism to meet diagnostic criteria at 18 months. Nevertheless, the CHAT had very high specificity (98%) and was the first demonstration internationally that autism could be diagnosed as early as 18 months of age, when previously age–at–first diagnosis was typically not until later in childhood.[16] The CHAT was based on toddler developmental precursors of 'theory of mind',[17] such as joint attention[18-20] and pretend play,[21] so stemmed from a cognitive developmental theory about the nature of autism.[22]

The Modified Checklist for Autism in Toddlers (M–CHAT),[7] is a 23–item parent–report questionnaire, used in the United States healthcare system. It incorporates the original nine items from the CHAT, and includes additional items that may be characteristic of a young autistic child. Initial results reported sensitivity of 0·97, specificity of 0·99, and positive predictive value (PPV) of 0·68 when used with a telephone follow–up. Despite limitations to the initial studies[23] (e.g. referred and unselected samples were combined, and no systematic follow–up), the M–CHAT has been examined in a large population sample at 18–months.[24] Specificity was high (0·93), but sensitivity was low (0·34) and PPV was 0·35. The authors concluded that it may not be possible to detect all autistic children at 18–months. The M–CHAT has subsequently been revised (M–CHAT, Revised with Follow–Up (M–CHAT–R/F)),[25] consisting of 20 items, including examples to provide developmental context and clarity. This was tested in a large population (N=16,071), finding PPV to be 0·48, while another study[26] reported PPV to be 0·54. Sensitivity could not be determined in these two studies because only the screen positives were followed up.

The Quantitative Checklist for Autism in Toddlers (Q–CHAT)[27] was developed to dimensionalise autism in toddlerhood: it has been demonstrated that autistic traits can be measured quantitatively in the general population, using the Autism Spectrum Quotient (AQ) in adulthood,[28] the AQ–Adolescent,[29] the AQ–Child[30] and the Social–Responsiveness Scale (SRS),[31] in older children and adults. The Q–CHAT is a parent–report 25–item questionnaire that includes the CHAT and additional items. Each item is converted to a rating scale, thus quantifying autistic traits. This allows for the possibility of reporting behaviour at a reduced frequency. Q–CHAT domains include social communication, repetitive, stereotyped and sensory behaviours. A preliminary study found Q–CHAT discriminated well between young autistic children and unselected toddlers in the population at 18–24 months.[27] A short version of the Q–CHAT (Q–CHAT–10)[32] was developed and tested retrospectively on toddlers with

Confidential: For Review Only

an autism diagnosis compared to toddlers from the unselected population reported. Sensitivity and specificity were 0·91, and 0·89 respectively. However, these studies were not longitudinal, so cannot address the question of whether it is best to screen for autism at single or multiple time–points.

The goal of this study is to report a population screening study of nearly 4,000 toddlers at 18–30 months using the Q–CHAT with diagnostic assessments on a sub–sample of responders, and subsequent follow–up at four years. Screening was conducted using the child health surveillance database to identify the population eligible to participate. The study is reported in two phases, as undertaken. The aim of Phase 1 was to determine test accuracy of the Q–CHAT in the toddler period. The aim of Phase 2 was to identify autistic children who were not detected at Phase 1. We used the Childhood Autism Spectrum Test (CAST)[33] and the Checklist for Referral (CFR) to seek information on the child's history of developmental concerns. We conducted diagnostic assessments to identify any missed cases (false negatives) from Phase 1, as well as to confirm the diagnostic outcome of those already identified. This two–phase design allowed us to report sensitivity, specificity and PPV. Phase 1 was not evaluated at the time to ensure researchers remained blind to the results when undertaking Phase 2.

Methods

Patients and Public Involvement Statement

Patients or the public WERE NOT involved in the design, or conduct, or reporting, or dissemination plans of our research

Setting, Study Design and Procedure

Phase 1

All caregivers of infants between the ages of 18-30 months who were registered on the Child Health Surveillance Database at the specified Primary Care Trusts (PCT) on the date of mailing were invited to complete the Q-CHAT between March 2008 and April 2009. Screening was conducted via a postal questionnaire, distributed by the PCT. Luton questionnaires were mailed in March 2008. Bedfordshire questionnaires were mailed in May 2008. Cambridgeshire questionnaires were mailed in April 2009. Questionnaires were posted twice, two weeks apart, to maximise response. Returned questionnaires were scored by the research team and the sampling strategy was applied to establish the assessment sample.

https://mc.manuscriptcentral.com/bmjpo
Confidential: For Review Only

Research diagnostic assessments were arranged as soon as possible after completion of the Q-CHAT. All members of the assessment team remained blind to the child's Q-CHAT score. The Q-CHAT was re-administered prior to any of the assessment battery. Diagnostic outcome was defined by the researchers reaching a consensus diagnosis using all assessment data and researcher judgement.

Phase 1 Sampling Strategy

Sampling for assessment was weighted towards those with higher scores. Determination of the best cut-point for the Q-CHAT was one aim of the study and a sampling cut-point of 44 was chosen as the point at which 100% of children who scored ≥ 44 would be selected for assessment. The rationale for choosing 44 as the sampling cut-point was based on anticipated estimates of prevalence of autism (approximately 1%) balanced with the knowledge that over 70% of children with autism scored above 44 in our initial study[27], maximising sensitivity whilst generating a feasible number of assessments to be completed. To ensure that children with missing data were not missed from being included in a high scoring group, Q-CHAT items that were missing were allocated the maximum item score (four) and the total Q-CHAT score recalculated. Each Q-CHAT questionnaire therefore had two scores: the observed (assuming each item of missing data would score as not impaired) and the maximum (assuming each item of missing data would score as impaired). Observed and maximum Q-CHAT scores were grouped into four bands (≥44, 41-43, 38-40, ≤37). This ensured that missing data meant a child was more likely to be assessed.

The final sampling groups were determined according to the maximum score, taking into account the movement across score groups from observed score to maximum score. Sampling group allocation and randomisation took place prior to establishing whether or not the family had consented for further contact.

When the chance of being selected was less than 100%, selection for assessment was undertaken using random.org 8. 1% of children who had a maximum score ≤37 were selected for assessment to ensure that the assessment team were blind to whether there were likely to be concerns about the child at the time of assessment.

Phase 2

https://mc.manuscriptcentral.com/bmjpo

Confidential: For Review Only

All caregivers who consented to further contact following the Phase 1 screening (N=3,472) were invited to participate in Phase 2 at a minimum age of 48 months, between January and July 2011. Caregivers were sent the Childhood Autism Spectrum Test (CAST), a 37-item tool to identify children at risk for autism in children aged 4–11 years, in a non-clinical setting[33, 34], Parents were also sent a brief checklist enquiring about whether the child had ever been referred or diagnosed with any developmental and/or medical condition (Checklist for Referral, CFR).

Phase 2 Sampling

All children who scored ≥15 on the CAST (the recommended screening cut-point for epidemiological research)[33] were invited for a Phase 2 diagnostic assessment, along with all children whose caregiver indicated on the CFR that they had been referred for any of the following reasons: language delay/disorder, Hyperactivity/Attention Deficit Disorder (ADHD, ADD), dyspraxia, autism spectrum condition (autism), genetic/chromosomal abnormality, epilepsy, Tourette syndrome, and tuberous sclerosis. There was also a space on the CFR for the caregiver to provide more information about the reason for the referral and whether any diagnosis had been made. If the caregiver indicated that their child had been referred for any other reason not specified on the CFR, the research team decided on a case–by–case basis whether or not to invite the family for the assessment. All children who participated in Phase 1 assessments were invited for a Phase 2 assessment.

Diagnostic Outcome

In both Phase 1 and 2, diagnostic outcome was defined by the researchers reaching a consensus diagnosis using all assessment data and researcher judgement as autism or autism spectrum (if they met the International Statistical Classification of Diseases 10th Edition (ICD-10) criteria[35] for a pervasive developmental disorders diagnosis), atypical (if the child showed developmental concerns that were not related to autism) or typical (if there were no developmental concerns about the child). It was not possible for all of the assessment team to remain blind to the diagnostic outcome from the Phase 1 assessments at Phase 2. However, the assessment team re-examined blind to the Q-CHAT score throughout Phase 2 assessments.

Confidential: For Review Only

Ethical approval was granted by the Cambridge Local Research Ethics Committee (05/Q0108/53). The University of Cambridge was the sponsor of this research. Informed consent was obtained from all caregivers included in the study.

Measures

Q-CHAT

The Q-CHAT has a forced choice design, with five possible responses for each item (with the exception of item 4, see below). For each item, response representing the most 'autistic' symptomatology scores four points and the least 'autistic' response scores zero. On approximately half the items (items 3, 7, 8, 11, 12, 13, 16, 18, 20, 22, 23, 24, 25) the 'autistic' response is the positive one. On the other half, (items 1, 2, 4, 5, 6, 9, 10, 14, 15, 17, 19, 21) the 'autistic' response is the negative one. Item 4 is concerned with speech and includes a sixth option of 'my child does not speak', also scoring four points. Total Q-CHAT score is obtained by summing the score on each item, giving a maximum of 100. Each of the 25 questions is accompanied by a colour illustration to attempt to increase both response and comprehensibility of the items.

Demographic Data

Data collected about the child included age, sex, birth order, ethnicity and multiple birth status. Data collected about the caregivers included Socio-Economic Status (SES) and parental educational attainment. SES was derived at person level rather than household level, using the five class system of the National Statistics-Socio Economic Classification (NS-SEC)[36]. Parental educational attainment was assessed by the highest level of qualification of each parent.

Diagnostic Assessment

The ADOS-G[37] was administered. The ADOS-G is a semi-structured, standardized assessment of social interaction, communication, play, and imagination. It is a reliable and valid measure for the diagnosis of autism relative to those with non-autistic conditions. Module 1 was chosen at Phase 1 since it was not expected that many children would have expressive language abilities beyond basic phrase speech. Module 2 or 3 was chosen at Phase 2. ADOS-G assessments were videotaped, but item scoring was conducted independently by two members of the assessment team and consensus codes were agreed as soon as possible after the assessment. The assessment team comprised of at least one very experienced

Confidential: For Review Only

research psychologist alongside a research assistant who also had been trained to reliability standards.

The Autism Diagnostic Interview-Revised (ADI-R)[38] was administered with the caregiver. The ADI-R is a diagnostic semi-structured interview that generates an algorithm score based on behaviours in three domains: social communication, social interaction and repetitive and stereotyped behaviours and interests. The interview also enquires about age of onset of the symptoms. Due to the practicalities in arranging the assessments, it was not possible to counterbalance administration of the ADOS-G and ADI-R. However, the ADI-R and ADOS-G was never carried out by the same researcher to reduce the possibility of experimenter expectation bias. All ADI-R assessments were audio-taped.

The Mullen Scales of Early Learning (MSEL)[39] is a standardised measure of cognitive, expressive and receptive language, motor and perceptual abilities. The MSEL was usually carried out while the other researcher was administering the ADI-R with the caregiver. The gross motor scale was not completed as this does not contribute to the overall Early Learning Composite (ELC). The Vineland Adaptive Behavior Scale (VABS)[40] is a standardised measure of personal and social skills. The interview version was administered to the parent. There are four domains that measure communication, daily living skills, socialisation and motor skills, as well as an optional maladaptive domain (not administered here). The VABS was usually administered at the end of the testing session.

Confidential: For Review Only

Table 1: Phase 1 sampling strategy, with study numbers

Sampling Group	Observed Q-CHAT (missing items = 0)	Maximum Q-CHAT (missing items = 4)	% sampled	Total	Assessment numbers		
					Selected	Invited	Agreed
1	≥38	≥44	100%	86	86	74	40
2	≤37	≥44	50%	21	10	9	6
3	41–43	41–43	50%	61	31	28	16
4	≤40	41–43	50%	21	10	8	5
5	38–40	38–40	25%	129	31	28	20
6	≤37	38–40	25%	25	5	5	2
7	≤37	≤37	1%	3300	40	39	32
**Total**				**3643**	**213**	**191**	**121**

Confidential: For Review Only

Statistical methods

Due to the stratified sample all analyses were adjusted for the probability of being selected into the assessment subsample at each Phase via inverse probability weighting. This takes account of the differences in sampling proportions and the selective participation in the assessment across the sampling groups. A sensitivity analysis also included within the weights are the health trust non–response rates at each stage. A receiver operator characteristic (ROC) area under the curve (AUC) analysis assessed the discriminant power of the Q–CHAT and CAST and CFR in distinguishing autism cases from non–autism cases (defined as typical and atypical) and from autism and all other atypical development from typically developing children, across all score values. Confidence intervals were obtained from robust standard errors. All analysis was undertaken in Stata 14.[41] A series of sensitivity analyses to decisions on the screening instrument were undertaken (see Table 2).

Role of the funding source

The funding source had no role in the data collection, data analysis, data interpretation, or writing of the report. The corresponding author had full access to all the data in the study and had final responsibility for the decision to submit for publication.

Results

Phase 1

3,770 Q–CHAT questionnaires were returned 436 (14% response) from Luton, 1,256 (25% response) from Bedfordshire and 2,078 (42% response) from Cambridgeshire. 223 were selected for assessment, 32 did not give consent to be contacted, therefore 191 were invited and 121 competed the assessments. See Table 1 for Phase 1 sampling strategy with study numbers, Supplementary Figure 1 for the flow chart from phase 1 and Supplementary Table 1 for characteristics of children and their families throughout the study, supplementary Figure 2 shows the distribution of Q–CHAT with sampling bands indicated.

Following assessment, 11 children were defined as autistic, 16 as atypical (7 language delay, 5 developmental delay, 4 other atypical), and 94 as typical. At a cut–point of ≥39, the positive predictive value (PPV) for autism was 17% (95%CI 8%–31%) (Table 2).

Confidential: For Review Only

Autism	TP	FN	FP	TN	Sensitivity	Specificity	PPV	NPV	ROC
**Main analysis – phase 1**									
Q–CHAT 39+	36	0	179	3428	1.00 (0.72–1.0)*	0.95 (0.92–0.97)	0.17 (0.08–0.31)	1.00 (0.93–1.0)*	0.98 (0.96–1.00)
**Main analysis – phase 2**									
**Q–CHAT 39+**	**27**	**34**	**69**	**3513**	**0.44 (0.26–0.64)**	**0.98 (0.97–0.99)**	**0.28 (0.15–0.46)**	**0.99 (0.98–0.99)**	**0.86 (0.78–0.94)**
CAST ≥15+ / CFR positive	50	7	98	3488	0.88 (0.63–0.97)	0.97 (0.95–0.98)	0.34 (0.23–0.46)	1.00 (0.99–1.00)	0.99 (0.98–1.00)
**Sensitivity analysis – phase 1**									
Q–CHAT 31+	36	0	1104	2504	1.00 (0.72–1.0)*	0.69 (0.53–0.82)	0.03 (0.01–0.07)	1.00 (0.88–1.0)*	Same as for 39+
Q–CHAT 38+	36	0	238	3369	1.00 (0.71–1.0)*	0.93 (0.90–0.96)	0.13 (0.06–0.25)	1.00 (0.92–1.0)*	Same as for 39+
Q–CHAT 39+ with parental concern	30	6	73	3534	0.84 (0.43–0.97)	0.98 (0.97–0.99)	0.29 (0.13–0.52)	1.00 (0.99–1.00)	1.00 (0.99–1.00)
Q–CHAT 39+ adjust initial non–response	49	0	201	3393	1.00 (0.72–1.0*)	0.94 (0.91–0.97)	0.20 (0.09–0.37)	1.00 (0.93–1.0)*	0.98 (0.96–1.00)
**Sensitivity analysis – phase 2**									
Q–CHAT 31+	48	13	589	2993	0.79 (0.64–0.89)	0.84 (0.65–0.93)	0.08 (0.03–0.19)	1.00 (0.99–1.00)	Same as for 39+
Q–CHAT 38+	35	26	92	3489	0.57 (0.39–0.74)	0.97 (0.96–0.99)	0.28 (0.16–0.43)	0.99 (0.99–1.00)	Same as for 39+
Q–CHAT 39+ with parental concern	24	37	29	3553	0.40 (0.23–0.60)	0.99 (0.98–1.00)	0.46 (0.24–0.69)	0.99 (0.98–0.99)	0.90 (0.84–0.96)
Q–CHAT 39+ adjust initial non–response	38	35	73	3497	0.51 (0.30–0.72)	0.98 (0.96–0.99)	0.34 (0.17–0.55)	0.99 (0.98–0.99)	0.89 (0.81–0.96)
CAST ≥15	32	25	8	3578	0.56 (0.37–0.74)	1.00 (0.99–1.00)	0.81 (0.59–0.92)	0.99 (0.99–1.00)	0.94 (0.88–0.99)
Q–CHAT 39+ include parent reported cases	30	34	67	3512	0.47 (0.30–0.65)	0.98 (0.97–0.99)	0.31 (0.52–0.82)	0.99 (0.98–0.99)	0.86 (0.79–0.94)

Table 2: Autism diagnosis results Phase 1 and Phase 2

Confidential: For Review Only

Phase 2

3,472 consenting caregivers from Phase 1 were sent the Phase 2 study materials. 2,005 returned both the CFR and CAST (58% response), a further 6 returned just the CAST and 5 returned just the CFR. 42 children who had an assessment in Phase 1 did not return the CAST or CFR.

172 caregivers reported developmental concerns on the CFR and 43 children scored 15 and above on the CAST (not in Phase 1 assessment sample) and were selected for assessment. 81 children who had been assessed in Phase 1 completed the assessment (1 child did not complete all the assessments and is therefore not included in the analysis). In total, 158 children completed the assessment at Phase 2, 23 with CFR concerns and CAST score ≥ 15, 63 with CFR concerns and CAST scores ≤ 15, 6 with CAST scores of ≥ 15 with no CFR concerns, 56 who scored ≤ 15 on the CAST and who did not report any developmental concerns on the CFR but who were assessed in Phase 1, and 10 children who were assessed in Phase 1 but who did not return the CFR or CAST (Figure 1 shows assessment flow and diagnostic outcome, with Figure 2 showing the characteristics of the two screening tests). 11 children were considered as possibly autistic (prevalence of 0·98%, 95%CI 0·45%–2·16%).

81 children were assessed at both Phase 1 and 2. Of the 9 children whose diagnostic outcome at Phase 1 was autism who took part in Phase 2, all were still classified as autism. A further 6 children were now classified as autism (4 who were typical at Phase 1, and 2 who were atypical). 77 children were assessed for the first time at Phase 2. Of these 29 were classified as autism, 24 as atypical development and 24 as typically developing, giving a prevalence of autism of 1·57% (95%CI 0·90%–2·74%). Figure 3 shows the study flow from Phase 1 to Phase 2.

Confidential: For Review Only

Insert Figure 1 here

Using the CAST alone as the screen at Phase 2, 1,994 children had complete data. At a cut–point of ≥15, sensitivity was 56% (95%CI 37%–74%) specificity was 100% (95%CI 99%–100%), and PPV was 81% (95%CI 59%–92%) (Table 2). The area under the ROC curve was 0·94 (95%CI 0·88–0·99) (Supplementary Figure 3). Adding in the CFR selection criteria improved sensitivity to 88% (95%CI 63%–97%) but decreased specificity slightly to 97% (95%CI 95%–98%), with a PPV of 34% (95%CI 23%–46%) and an area under the ROC curve of 0·99 (95%CI 0·98–1·00).

At a cut–point of ≥39, the sensitivity of the Q–CHAT to predict Phase 2 outcome (autism vs atypical and/or typical) was 44% (95%CI 26%–64%), specificity was 98% (95%CI 97%–99%), and PPV was 28% (95%CI 15%–46%) (Table 2). The area under the ROC curve was 0·86 (95%CI 0·78–0·94).

Sensitivity analyses: Optimising the Q–CHAT cut–point for Phase 2 outcome found two additional cut–points, Q–CHAT 31+ maximized sensitivity and specificity at the cost of false positives and using Q–CHAT 38+ marginally improved sensitivity with little additional benefit. Other sensitivity analyses at Phase 1 and Phase 2 are shown in Table 2. See Figure 2 for characteristics and comparison of the screening instruments.

Insert Figure 2 here

Insert Figure 3 here

Discussion

At Phase 1 outcome, all children who were classified as autistic failed the Q–CHAT at a cut–point of 39, demonstrating that early detection and diagnosis of autism is possible. The PPV of the Q–CHAT for autism was 17%. Misinterpretation of behaviours that are not well–established as well as developmental immaturity may contribute to low PPV. In Phase 2 we identified autistic children at age 4 who were not identified by the Q–CHAT. At a cut–point of ≥39, the sensitivity of the Q–CHAT to predict autism was 44% (95%CI 26%–64%), specificity was 98% (95%CI 97%–99%), and PPV was 28% (95%CI 15%–46%). This demonstrates that the Q–CHAT alone cannot be used to detect all autistic children. This might reflect the Q–CHAT properties, but equally it likely reflects a subgroup of autistic

https://mc.manuscriptcentral.com/bmjpo
Confidential: For Review Only

children who do not show symptoms of sufficient 'severity' until later in childhood, and/or that symptoms of autism change with age, so require developmentally sensitive instruments at different ages. The CAST picked up many of the cases of autism that were missed by the Q–CHAT, demonstrating the need for repeat screening rather than relying on a single instrument at one time point.. However, the sensitivity of the CAST at a cut point of 15 was only moderate (56%), compared to our earlier study showing sensitivity of 100%.[33]

A strength of this study was the prospective, population–based design that allowed us to assess the Q–CHAT accurately at two time–points. Our approach to missing data and subsequent sampling strategy was extremely cautious and conservative, and ensured maximum capture of potential cases in the assessment phase, maximising sensitivity. However, studies such as the current one are challenged by modest participation rates, selection bias and attrition over time. Despite adopting strategies to attempt to increase response, overall it was low, but not unusual for an unsolicited postal survey. Whether or not those who responded initially to the Q–CHAT (which determined the population for the rest of the study) were biased towards those with concerns (either justified or the 'worried well') or in the opposite direction remains largely unknown. A second limitation is that in Phase 1, only 1% of children who scored ≤37 were sampled. This proportion was chosen for pragmatic reasons in relation to feasibility (i.e. to keep the number of lengthy diagnostic assessments in children with no developmental concerns to a minimum, and to reduce expectation bias in the research team). None of the sampled children with scores ≤37 met our assessment case definition. It is important to underline that these results are based purely on outcome at Phase 1 and do not reflect the overall test accuracy statistics from Phase 2.

Few studies attempt population screening, partly because they are very time consuming and expensive. Younger toddlers have mild or transient developmental delays that resolve, resulting in low PPV, as was found in another study.[42] Introducing a follow–up interview (such as used with the M–CHAT[23, 25, 26]) for those who screen positive may reduce the number of unnecessary referrals for a diagnostic assessment, especially with children older than 20 months.[43] Nevertheless, a recent meta–analysis of M–CHAT studies found a lack of evidence on its performance in low–risk children or at age 18 months.[44] A universal, primary care-based screening study was conducted using the Modified Checklist for Autism in Toddlers with Follow-Up (M-CHAT/F) in the United States. Overall, the sensitivity was 38.8%, and PPV was 14.6%. Sensitivity was higher in older toddlers and with repeated

Confidential: For Review Only

screenings, whereas PPV was lower in girls[45]. This study was the first using the M-CHAT to follow up children screened in the population, thus allowing for an accurate estimate of the sensitivity of the instrument. Since the publication of the original CHAT[5, 6, 16] no study in the UK to date has achieved screening data on such a large population sample of toddlers and accurately determined sensitivity through follow up.

Overall, this study demonstrates that (1) that the Q–CHAT detected every child who received an autism diagnosis at Phase 1 outcome and therefore it is possible to detect autism at 18–30 months; and (2) it is not possible to detect every child at a very young age who will later be diagnosed as autistic. The Q–CHAT can be used to screen toddlers at 18–30 months for autism to enable accelerated referral into the diagnostic pathway. However, the PPV is low, thus potentially generating a high number of children who may not have autism, but who may still require a developmental assessment to determine whether they warrant a referral for intervention and support. Outcome data from Phase 2 revealed many autistic children that the Q–CHAT did not identify. The autism spectrum is so broad that it is not reasonable to expect that every child will be detectable as a young toddler. Therefore, when looking at the Q–CHAT longitudinally, it is evident that screening at a single time–point results in less than optimal test properties. We suspect that the missed cases reflects a subgroup of autistic whose symptoms are not severe enough to warrant a diagnosis during toddlerhood, and that the symptoms of autism change with age. We therefore recommend continued surveillance and re–screening at multiple time–points using developmentally sensitive instruments in order to identify a higher proportion of cases for maximal public health impact. This study demonstrates that no single instrument will detect all cases of autism at a single time point. Therefore, continued monitoring throughout the life course is necessary in order to identify when autistic traits begin to impair an individual's life. High quality evidence from random controlled trials is needed to determine whether early detection and consequent early implementation of specific interventions is able to change outcomes in autistic toddlers.

Total Q–CHAT score was used to determine which sampling band each child fell into. A sum (total) score based on equal weighting of all items may not the most appropriate method by which to quantify an individual's autistic traits. Other work by our group has shown that there are at least two items on the Q–CHAT whereby the prevalence of endorsement of the trait in the autistic direction is lower for toddlers with a diagnosis of autism, compared to population controls (items 8 and 18).[32] Item response analysis will be conducted to determine whether

Confidential: For Review Only

every item equally contributes useful information about the underlying latent dimensions. Defining a threshold on each factor that can be measured quantitatively (continuous approach) may be a more appropriate scoring method. Furthermore, exploration of different age–specific scoring algorithms should be implemented to determine which variables carry greatest relative importance to optimise screening results.[2, 43]

Word Count: 5571

Figure 1: Assessment flow and diagnostic outcome

Figure 2: Characteristics and comparison of the screening instruments (not weighted)

Figure 3: Study flow from Phase 1 to Phase 2

Funding: The study was funded by the National Lottery Community Fund, the Autism Research Trust, and was conducted in association with the Gillings Family Charitable Trust and the National Institute for Health Research (NIHR) Collaboration for Leadership in Applied Health Research and Care (CLAHRC) East of England at Cambridgeshire and Peterborough NHS Foundation Trust, and by the NIHR Cambridge Biomedical Research Centre. The views expressed are those of the author(s) and not necessarily those of the NHS, the NIHR or the Department of Health. SBC and TC were supported by the Medical Research Council UK and the Innovative Medicines Initiative (IMI) 2 Joint Undertaking (JU) under grant agreement no. 777394. FEM was supported by MRC U105292687. The JU receives support from the European Union's Horizon 2020 research and innovation programme and EFPIA and Autism Speaks, Autistica, and SFARI. SBC and CA were also supported by the Templeton World Charitable Foundation and the Wellcome Trust during the period of this work. Grant numbers not applicable.

Acknowledgements: We are grateful to the families who participated in all phases of the study. We are also grateful to staff at Luton, Bedfordshire and Cambridgeshire CCGs for their cooperation in defining the target population and mailing the questionnaires. We are grateful to Gina Gomez, Kristelle Hudry, Sally Clifford, Georgina Woods, Emma Robson, Philippa Lewington, Susan Sadek, Kimberly Armstrong, Martine Roelfsema and Kim Davies for help with data collection.

Author Contribution: CA, FM, CB, TC and SB–C contributed to the study design. CA, LR, GP and RS collected the data. CA and FEM did the data analysis and FEM is guarantor of the analysis.

https://mc.manuscriptcentral.com/bmjpo
Confidential: For Review Only

All authors contributed to data interpretation and writing of the report.

Competing Interests: Dr. Charman has received research grant support from the Medical Research Council (UK), the National Institute for Health Research, Horizon 2020 and the Innovative Medicines Initiative (European Commission), MQ, Autistica, FP7 (European Commission), the Charles Hawkins Fund, and the Waterloo Foundation. He has served as a consultant to F. Hoffmann–La Roche Ltd. He has received royalties from Sage Publications and Guilford Publications. All other authors report no biomedical financial interests or potential conflicts of interest.

Transparency declaration: The lead author and guarantor affirms that the manuscript is an honest, accurate, and transparent account of the study being reported; that no important aspects of the study have been omitted; and that any discrepancies from the study as planned and registered have been explained.

Dissemination to participants and related patient and public communities: The authors plan to send a summary of the results to participating NHS Trusts. A lay summary of the results will be available on the Autism Research Centre's website. Results will be presented at international conferences.

Patient and public involvement: Patients or the public were not involved in the design, or conduct, or reporting, or dissemination plans of our research.

Data sharing: At the time of data collection, consent was not obtained from participants for the purposes of data sharing. Therefore even anonymised data are not available.

Confidential: For Review Only

References

1. Baron-Cohen S, Scott FJ, Allison C, Williams J, Bolton P, Matthews FE, et al. Prevalence of autism-spectrum conditions: UK school-based population study. Br J Psychiatry. 2009;194(6):500-9.
2. Zwaigenbaum L, Bauman ML, Fein D, Pierce K, Buie T, Davis PA, et al. Early Screening of Autism Spectrum Disorder: Recommendations for Practice and Research. Pediatrics. 2015;136 Suppl 1:S41-59.
3. National Institute for Health and Clinical Excellence. Autism: recognition, referral and diagnosis of children and young people on the autism spectrum. (Clinical guideline 128). https://www.nice.org.uk/guidance/cg128; 2011.
4. Johnson CP, Myers SM. Identification and Evaluation of Children With Autism Spectrum Disorders. Pediatrics. 2007;120(5):1183-215.
5. Baron-Cohen S, Allen J, Gillberg C. Can autism be detected at 18 months? The needle, the haystack, and the CHAT. Br J Psychiatry. 1992;161:839-43.
6. Baron-Cohen S, Cox A, Baird G, Swettenham J, Nightingale N, Morgan K, et al. Psychological markers in the detection of autism in infancy in a large population. Br J Psychiatry. 1996;168(2):158-63.
7. Robins DL, Fein D, Barton ML, Green JA. The Modified Checklist for Autism in Toddlers: an initial study investigating the early detection of autism and pervasive developmental disorders. J Autism Dev Disord. 2001;31(2):131-44.
8. Al-Qabandi M, Gorter JW, Rosenbaum P. Early autism detection: are we ready for routine screening? Pediatrics. 2011;128(1):e211-7.
9. Siu AL, and the U. S. Preventive Services Task Force. Screening for autism spectrum disorder in young children: Us preventive services task force recommendation statement. JAMA. 2016;315(7):691-6.
10. Crane L, Chester JW, Goddard L, Henry LA, Hill E. Experiences of autism diagnosis: A survey of over 1000 parents in the United Kingdom. Autism. 2016;20(2):153-62.
11. Brett D, Warnell F, McConachie H, Parr JR. Factors Affecting Age at ASD Diagnosis in UK: No Evidence that Diagnosis Age has Decreased Between 2004 and 2014. J Autism Dev Disord. 2016;46(6):1974-84.
12. Zwaigenbaum L, Bauman ML, Choueiri R, Kasari C, Carter A, Granpeesheh D, et al. Early Intervention for Children With Autism Spectrum Disorder Under 3 Years of Age: Recommendations for Practice and Research. Pediatrics. 2015;136 Suppl 1:S60-81.
13. Leekam S, Tandos J, McConachie H, Meins E, Parkinson K, Wright C, et al. Repetitive behaviours in typically developing 2-year-olds. J Child Psychol Psychiatry. 2007;48(11):1131-8.
14. Leekam SR, Prior MR, Uljarevic M. Restricted and repetitive behaviors in autism spectrum disorders: a review of research in the last decade. Psychol Bull. 2011;137(4):562-93.
15. Williams AN, Mold B, Kilbey L, Naganna P. Forty years of referrals and outcomes to a UK Child Development Centre (CDC): Has demand plateaued? Child Care Health Dev. 2018.
16. Baird G, Charman T, Baron-Cohen S, Cox A, Swettenham J, Wheelwright S, et al. A screening instrument for autism at 18 months of age: a 6-year follow-up study. J Am Acad Child Adolesc Psychiatry. 2000;39(6):694-702.
17. Baron-Cohen S. Autism: A specific cognitive disorder of "mind-blindness.". International Review of Psychiatry. 1990;2(1):81-90.
18. Baron-Cohen S. Perceptual role taking and protodeclarative pointing in autism. British Journal of Developmental Psychology. 1989;7(2):113-27.

Confidential: For Review Only

19. Baron-Cohen S. Joint-attention deficits in autism: Towards a cognitive analysis. Development and Psychopathology. 1989;1(3):185-9.
20. Goodhart F, Baron-Cohen S. How many ways can the point be made? Evidence from children with and without autism. First Language. 1993;13(38, Pt 2):225-33.
21. Baron-Cohen S. Autism and symbolic play. British Journal of Developmental Psychology. 1987;5(2):139-48.
22. Charman T, Baron-Cohen S, Swettenham J, Baird G, Cox A, Drew A. Testing joint attention, imitation, and play as infancy precursors to language and theory of mind. Cognitive Development. 2000;15(4):481-98.
23. Kleinman JM, Robins DL, Ventola PE, Pandey J, Boorstein HC, Esser EL, et al. The modified checklist for autism in toddlers: a follow-up study investigating the early detection of autism spectrum disorders. J Autism Dev Disord. 2008;38(5):827-39.
24. Stenberg N, Bresnahan M, Gunnes N, Hirtz D, Hornig M, Lie KK, et al. Identifying children with autism spectrum disorder at 18 months in a general population sample. Paediatr Perinat Epidemiol. 2014;28(3):255-62.
25. Robins DL, Casagrande K, Barton M, Chen CM, Dumont-Mathieu T, Fein D. Validation of the modified checklist for Autism in toddlers, revised with follow-up (M-CHAT-R/F). Pediatrics. 2014;133(1):37-45.
26. Chlebowski C, Robins DL, Barton ML, Fein D. Large-scale use of the modified checklist for autism in low-risk toddlers. Pediatrics. 2013;131(4):e1121-7.
27. Allison C, Baron-Cohen S, Wheelwright S, Charman T, Richler J, Pasco G, et al. The Q-CHAT (Quantitative CHecklist for Autism in Toddlers): a normally distributed quantitative measure of autistic traits at 18-24 months of age: preliminary report. J Autism Dev Disord. 2008;38(8):1414-25.
28. Baron-Cohen S, Wheelwright S, Skinner R, Martin J, Clubley E. The autism-spectrum quotient (AQ): evidence from Asperger syndrome/high-functioning autism, males and females, scientists and mathematicians. J Autism Dev Disord. 2001;31(1):5-17.
29. Baron-Cohen S, Hoekstra RA, Knickmeyer R, Wheelwright S. The Autism-Spectrum Quotient (AQ)--adolescent version. J Autism Dev Disord. 2006;36(3):343-50.
30. Auyeung B, Baron-Cohen S, Wheelwright S, Allison C. The Autism Spectrum Quotient: Children's Version (AQ-Child). J Autism Dev Disord. 2008;38(7):1230-40.
31. Constantino JN, Davis SA, Todd RD, Schindler MK, Gross MM, Brophy SL, et al. Validation of a brief quantitative measure of autistic traits: comparison of the social responsiveness scale with the autism diagnostic interview-revised. J Autism Dev Disord. 2003;33(4):427-33.
32. Allison C, Auyeung B, Baron-Cohen S. Toward brief "Red Flags" for autism screening: The Short Autism Spectrum Quotient and the Short Quantitative Checklist for Autism in toddlers in 1,000 cases and 3,000 controls [corrected]. J Am Acad Child Adolesc Psychiatry. 2012;51(2):202-12 e7.
33. Williams J, Scott F, Stott C, Allison C, Bolton P, Baron-Cohen S, et al. The CAST (Childhood Asperger Syndrome Test): test accuracy. Autism. 2005;9(1):45-68.
34. Scott FJ, Baron-Cohen S, Bolton P, Brayne C. The CAST (Childhood Asperger Syndrome Test): preliminary development of a UK screen for mainstream primary-school-age children. Autism. 2002;6(1):9-31.
35. World Health Organization. The ICD-10 Classification of Mental and Behavioural Disorders: Diagnostic Criteria for Research. Geneva: WHO; 1993.
36. Statistics. OfN. Self–coded version of the NS–SEC 2008. Available from: http://www.ons.gov.uk/about–statistics/classifications/current/ns–sec/self–coded/index.html.
37. Lord C, Risi S, Lambrecht L, Cook EH, Jr., Leventhal BL, DiLavore PC, et al. The autism diagnostic observation schedule-generic: a standard measure of social and

Confidential: For Review Only

communication deficits associated with the spectrum of autism. J Autism Dev Disord. 2000;30(3):205-23.
38. Lord C, Rutter M, Le Couteur A. Autism Diagnostic Interview-Revised: a revised version of a diagnostic interview for caregivers of individuals with possible pervasive developmental disorders. J Autism Dev Disord. 1994;24(5):659-85.
39. Mullen EM. Mullen Scales of Early Learning manual. AGS ed. Circle Pines, MN: American Guidance Service; 1995. ix, 85 p. p.
40. Sparrow SS, Balla DA, Cicchetti DV. Vineland adaptive behavior scales: Survey forms manual: AGS Publ.; 2005.
41. StataCorp. Stata Statistical Software: Release 14. College Station, TX: StataCorp LP; 2015.
42. Pandey J, Verbalis A, Robins DL, Boorstein H, Klin AM, Babitz T, et al. Screening for autism in older and younger toddlers with the Modified Checklist for Autism in Toddlers. Autism. 2008;12(5):513-35.
43. Sturner R, Howard B, Bergmann P, Morrel T, Landa R, Walton K, et al. Accurate Autism Screening at the 18-Month Well-Child Visit Requires Different Strategies than at 24 Months. J Autism Dev Disord. 2017.
44. Yuen T, Penner M, Carter MT, Szatmari P, Ungar WJ. Assessing the accuracy of the Modified Checklist for Autism in Toddlers: a systematic review and meta-analysis. Dev Med Child Neurol. 2018.
45. Guthrie W, Wallis K, Bennett A, Brooks E, Dudley J, Gerdes M, et al. Accuracy of Autism Screening in a Large Pediatric Network. Pediatrics. 2019;144(4).

Confidential: For Review Only

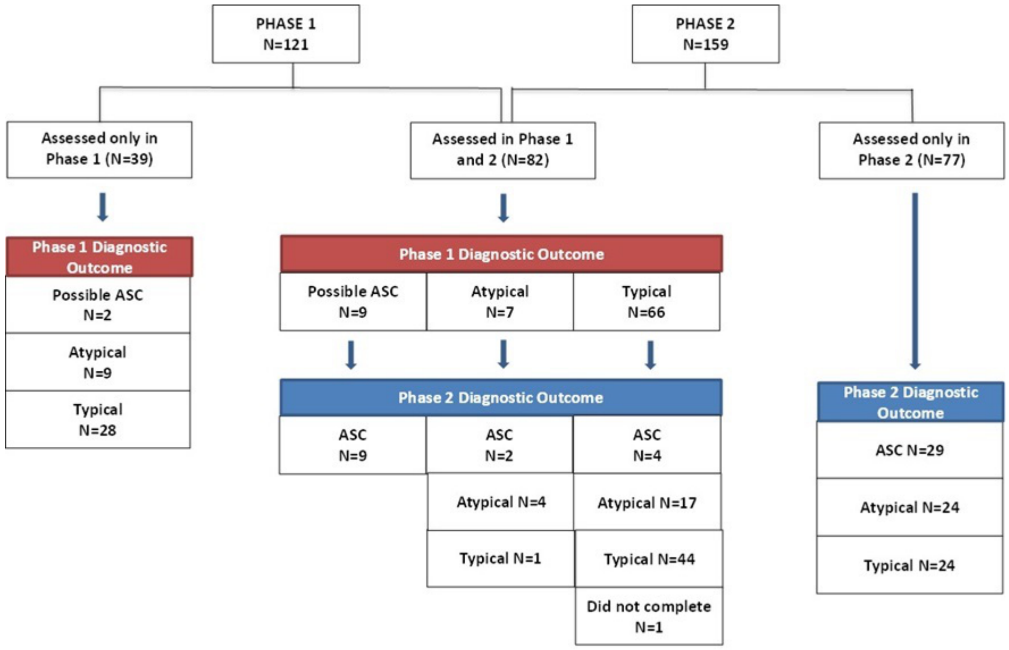
PHASE 1
N=121
PHASE 2
N=159
Assessed only in Phase 1 (N=39)
Assessed in Phase 1 and 2 (N=82)
Assessed only in Phase 2 (N=77)
Phase 1 Diagnostic Outcome
Possible ASC N=2
Atypical N=9
Typical N=28
Phase 1 Diagnostic Outcome
Possible ASC N=9
Atypical N=7
Typical N=66
Phase 2 Diagnostic Outcome
ASC N=9
ASC N=2
ASC N=4
Atypical N=4
Atypical N=17
Typical N=1
Typical N=44
Did not complete N=1
Phase 2 Diagnostic Outcome
ASC N=29
Atypical N=24
Typical N=24

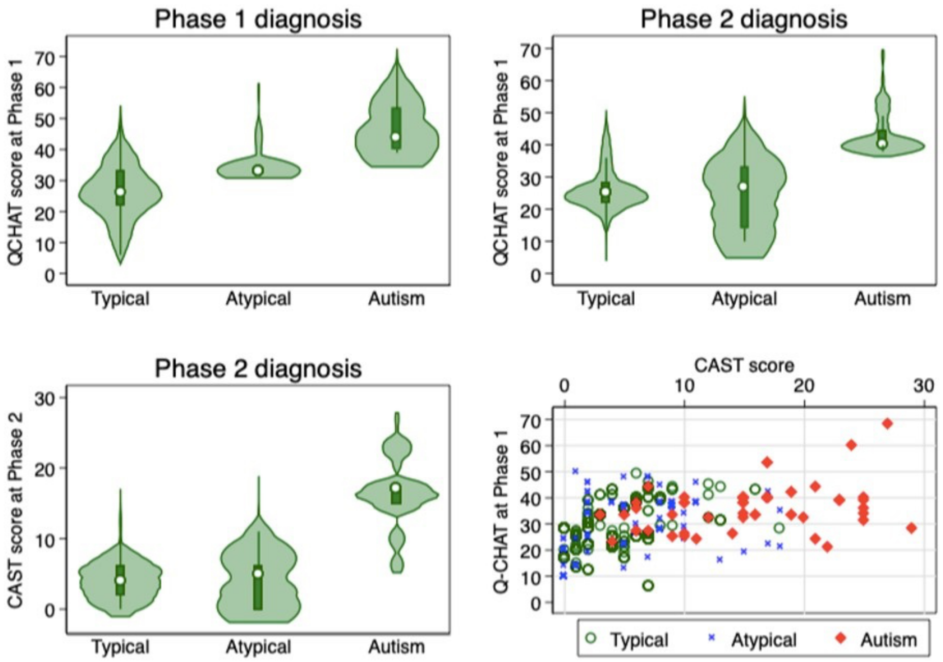
Phase 1 diagnosis
QCHAT score at Phase 1
Typical
Atypical
Autism
Phase 2 diagnosis
QCHAT score at Phase 1
Typical
Atypical
Autism
Phase 2 diagnosis
CAST score at Phase 2
Typical
Atypical
Autism
CAST score
Q-CHAT at Phase 1
Typical
Atypical
Autism

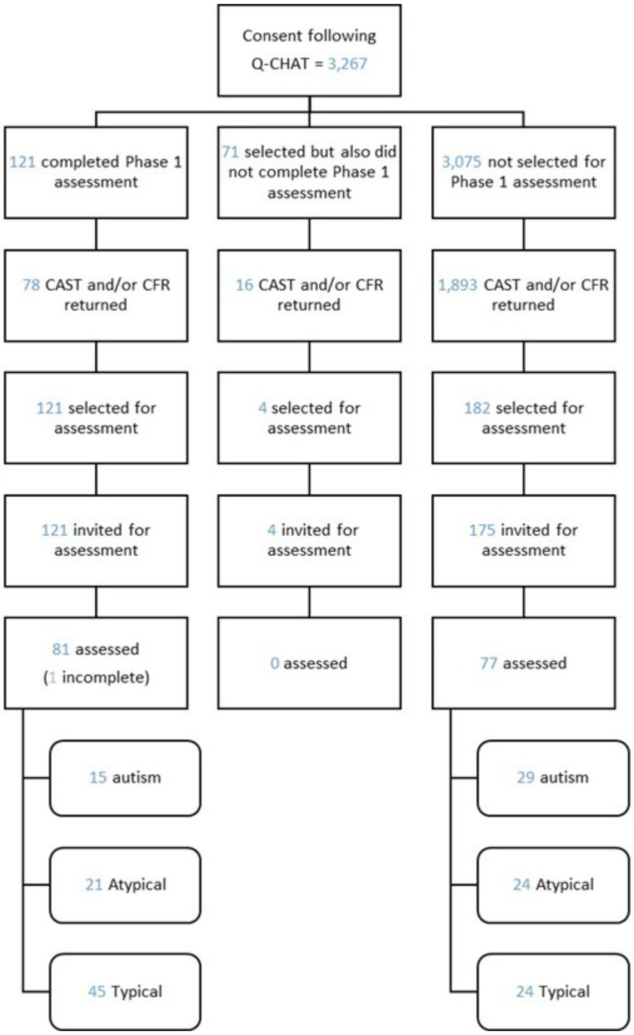
Consent following Q-CHAT = 3,267
121 completed Phase 1 assessment
71 selected but also did not complete Phase 1 assessment
3,075 not selected for Phase 1 assessment
78 CAST and/or CFR returned
16 CAST and/or CFR returned
1,893 CAST and/or CFR returned
121 selected for assessment
4 selected for assessment
182 selected for assessment
121 invited for assessment
4 invited for assessment
175 invited for assessment
81 assessed (1 incomplete)
0 assessed
77 assessed
15 autism
21 Atypical
45 Typical
29 autism
24 Atypical
24 Typical

Supplementary Appendix

Supplementary Table 1: Study Characteristics

		Phase I				Phase II			
		Total		Assessment		Total		Assessment	
PCT	Luton	416	(11%)	22	(18%)	176	(9%)	19	(12%)
	Bedfordshire	1217	(33%)	39	(32%)	638	(32%)	55	(35%)
	Cambridgeshire	2018	(55%)	60	(50%)	1183	(59%)	84	(53%)
Child sex	Female	1759	(48%)	42	(35%)	972	(49%)	56	(35%)
	Male	1884	(52%)	79	(65%)	1025	(51%)	102	(65%)
	Unknown*	70		0		1		0	
Age child at Phase I	16–19 months	251	(7%)	15	(12%)	142	(7%)	13	(8%)
	20–24 months	1478	(41%)	49	(40%)	831	(42%)	69	(44%)
	25–29 months	1474	(40%)	46	(38%)	814	(41%)	66	(42%)
	30–34 months	440	(12%)	11	(9%)	210	(11%)	10	(6%)
	Unknown*	125		0		6		0	

* Children with unknown sex or age were excluded from the analysis and not included within the rest of the table

Confidential: For Review Only

		Phase I				Phase II			
		Total		Assessment		Total		Assessment	
Mothers age at birth (Median, IQR)		32	[28,35]	31	[27,36]	33	[29,36]	32	[29,36]
Mothers education	None / <5 GCSE	480	(13%)	25	(21%)	166	(8%)	19	(12%)
	5 GCSE	548	(15%)	21	(17%)	252	(13%)	24	(15%)
	2+ A levels	512	(14%)	11	(9%)	255	(13%)	15	(9%)
	Degree	1684	(45%)	48	(40%)	1068	(53%)	80	(51%)
	Unknown	546	(14%)	16	(13%)	267	(13%)	20	(13%)
Mother occupation	Not working	1545	(41%)	58	(48%)	736	(37%)	67	(42%)
	Student	86	(2%)	4	(3%)	40	(2%)	5	(3%)
	Employed	1044	(28%)	28	(23%)	592	(29%)	43	(27%)
	Self employed no employees	176	(5%)	5	(4%)	105	(5%)	9	(6%)
	Self employed with employees, employed supervisor	835	(22%)	22	(18%)	513	(26%)	31	(20%)
	Unknown	84	(2%)	4	(3%)	22	(1%)	3	(2%)
Mother	White	3236	(86%)	94	(78%)	1795	(89%)	141	(89%)
	Black (African or Caribbean)	103	(3%)	6	(5%)	36	(2%)	4	(3%)
	South East Asian	231	(5%)	13	(11%)	78	(4%)	7	(4%)
	Other / Unknown	200	(5%)	8	(7%)	99	(5%)	6	(4%)

Confidential: For Review Only

		Phase I				Phase II			
		Total		Assessment		Total		Assessment	
Fathers age at birth (Median, IQR)		34	[31,38]	34	[31,37]	35	[31,39]	35	[31,39]
Father education	None / <5 GCSE	613	(16%)	22	(18%)	268	(13%)	23	(15%)
	5 GCSE	477	(13%)	11	(9%)	223	(11%)	14	(9%)
	2+ A levels	439	(12%)	14	(12%)	232	(12%)	22	(14%)
	Degree	1547	(41%)	41	(34%)	983	(49%)	62	(39%)
	Unknown	694	(18%)	33	(27%)	302	(15%)	37	(23%)
Father occupation	Not working	166	(4%)	10	(8%)	61	(3%)	5	(3%)
	Student	40	(1%)	1	(1%)	14	(1%)	1	(1%)
	Employed	1153	(31%)	38	(31%)	634	(32%)	51	(32%)
	Self–employed – no employees	351	(9%)	11	(9%)	196	(10%)	20	(13%)
	Self–employed – with employees, employed supervisor	1729	(46%)	41	(34%)	994	(49%)	66	(42%)
	Unknown	331	(9%)	20	(17%)	109	(5%)	15	(9%)
Father ethnicity	White	3171	(84%)	96	(79%)	1780	(87%)	138	(87%)
	Black (African or Caribbean)	142	(4%)	8	(7%)	52	(3%)	6	(4%)
	South East Asian	237	(6%)	10	(8%)	78	(4%)	6	(4%)
	Other / Unknown	220	(6%)	7	(6%)	98	(5%)	8	(5%)

Supplementary Figure 1: Phase 1 study flow

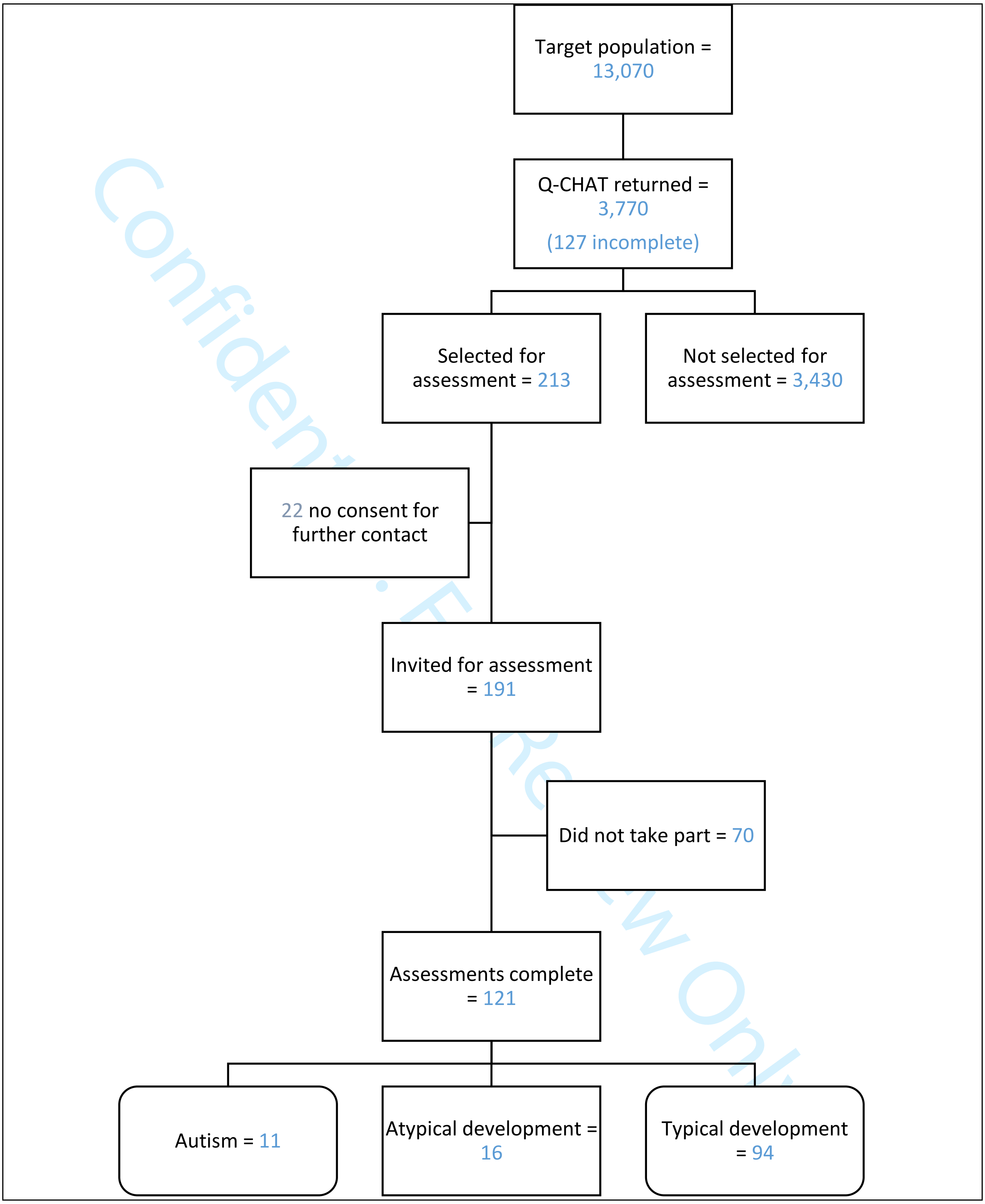

Confidential: For Review Only

Supplementary Figure 2: Distribution of Q–CHAT at first interview, with marks for screening groups

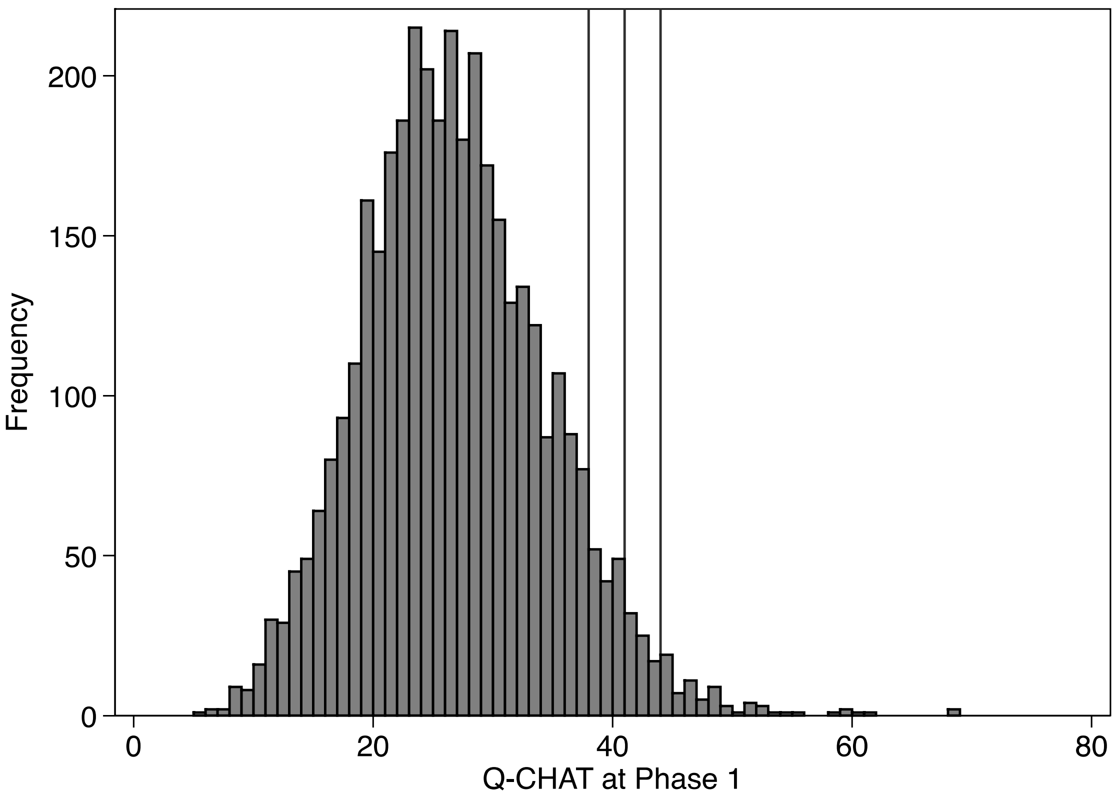

Review Only

Supplementary Figure 3: ROC curves for Q–CHAT and CAST on Phase 1 and Phase 2 outcomes for autism

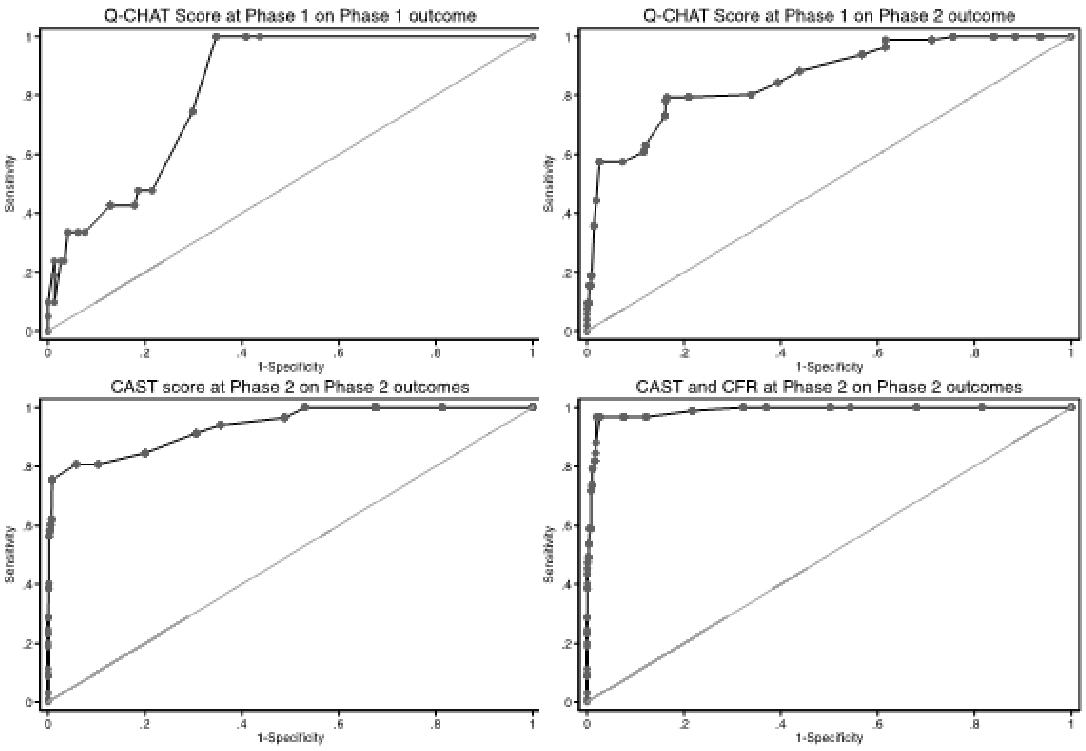

For Peer Review Only

BMJ Paediatrics Open

The Quantitative Checklist for Autism in Toddlers (Q–CHAT). A population screening study with follow-up: the case for multiple time–point screening for autism.

Journal:	*BMJ Paediatrics Open*
Manuscript ID	bmjpo-2020-000700.R1
Article Type:	Original research
Date Submitted by the Author:	08-Jan-2021

Complete List of Authors:	Allison, Carrie; Autism Research Centre, Psychiatry Department<br>Matthews, Fiona; Newcastle University, Population Health Sciences Institute, Campus for Ageing and Vitality<br>Ruta, Liliana; National Research Council, Institute of Applied Sciences and Intelligent Systems, "Eduardo Caianiello" (ScienceApp)<br>Pasco, Greg; Institute of Psychiatry Psychology and Neuroscience, Psychology<br>Soufer, Renee; Autism Research Centre, Psychiatry Department<br>Brayne, Carol; University of Cambridge, Institute of Public Health<br>Charman, Tony; King's College London, Institute of Psychiatry, Department of Psychology<br>Baron-Cohen, Simon; Autism Research Centre, Psychiatry Department

Keywords:	Autism, Screening

SCHOLARONE™
Manuscripts

I, the Submitting Author has the right to grant and does grant on behalf of all authors of the Work (as defined in the below author licence), an exclusive licence and/or a non-exclusive licence for contributions from authors who are: i) UK Crown employees; ii) where BMJ has agreed a CC-BY licence shall apply, and/or iii) in accordance with the terms applicable for US Federal Government officers or employees acting as part of their official duties; on a worldwide, perpetual, irrevocable, royalty-free basis to BMJ Publishing Group Ltd ("BMJ") its licensees and where the relevant Journal is co-owned by BMJ to the co-owners of the Journal, to publish the Work in this journal and any other BMJ products and to exploit all rights, as set out in our licence.

The Submitting Author accepts and understands that any supply made under these terms is made by BMJ to the Submitting Author unless you are acting as an employee on behalf of your employer or a postgraduate student of an affiliated institution which is paying any applicable article publishing charge ("APC") for Open Access articles. Where the Submitting Author wishes to make the Work available on an Open Access basis (and intends to pay the relevant APC), the terms of reuse of such Open Access shall be governed by a Creative Commons licence – details of these licences and which Creative Commons licence will apply to this Work are set out in our licence referred to above.

Other than as permitted in any relevant BMJ Author's Self Archiving Policies, I confirm this Work has not been accepted for publication elsewhere, is not being considered for publication elsewhere and does not duplicate material already published. I confirm all authors consent to publication of this Work and authorise the granting of this licence.

Confidential: For Review Only

The Quantitative Checklist for Autism in Toddlers (Q–CHAT). A population screening study with follow-up: the case for multiple time–point screening for autism.

Confidential: For Review Only

Allison, C.*[1], Matthews, F.E. [2], Ruta, L.[1,3], Pasco, G.[4], Soufer, R.[1], Brayne, C.[5], Charman, T.[4], & Baron–Cohen, S. [1]

[1] Autism Research Centre, University of Cambridge, Department of Psychiatry, Douglas House, 18B Trumpington Road, Cambridge, CB2 8AH, UK

[2] Institute of Health and Society, Campus for Ageing and Vitality, Newcastle University, Newcastle upon Tyne, NE4 5PL, UK

[3] Institute of Applied Sciences and Intelligent Systems, "Eduardo Caianiello" (ScienceApp), National Research Council of Italy (CNR), Messina, Italy.

[4] Department of Psychology, Institute of Psychiatry, Psychology & Neuroscience, King's College London, DeCrespigny Park, Denmark Hill, London, SE5 8AF, UK

[5] Department of Public Health and Primary Care, Cambridge Institute of Public Health, University of Cambridge, CB2 0SR

* Corresponding author cla29@cam.ac.uk tel 01223 465233

Objectives: Prospective population screening study for autism in 18-30-month-old toddlers using the Quantitative Checklist for Autism in Toddlers (Q-CHAT), with follow-up at age 4 years.

Design: Observational Study.

Setting: Luton and Bedfordshire, and Cambridgeshire in the United Kingdom.

Participants: 13,070 toddlers registered on the Child Health Surveillance Database between March 2008 and April 2009, with follow–up at age 4; 3,770 (29%) were screened for autism at 18–30 months using the Q–CHAT and the Childhood Autism Screening Test (CAST) at follow up at age 4 years.

Interventions: A stratified sample across the Q-CHAT score distribution was invited for diagnostic assessment (Phase 1). The 4 year follow–up included the Childhood Autism Screening Test (CAST) and Checklist for Referral (CFR). All with CAST ≥15, Phase 1 diagnostic assessment, or, with developmental concerns on the CFR were invited for diagnostic assessment (Phase 2). Standardised diagnostic assessment at both time-points was conducted to establish the test accuracy of the Q-CHAT.

Main outcome measures: Consensus diagnostic outcome at Phase 1 and Phase 2.

Results: Phase 1: 3,770 Q–CHATs were returned (29% response), 121 undertook diagnostic assessment, of whom 11 met criteria for autism. All 11 screened positive on the Q–CHAT. The positive predictive value (PPV) at a cut–point of 39 was 17% (95%CI 8%–31%). Phase 2: 2,005/3,472 CASTs were returned (58% response). 159 underwent diagnostic assessment, including 82 assessed in Phase 1. All children meeting criteria for autism were identified via the Q–CHAT at Phase 1 also met criteria at Phase 2. PPV was 28% (95%CI 15%–46%).

Conclusions: The Q–CHAT can be used at 18–30 months to identify autism and enable accelerated referral for diagnostic assessment. For every true positive there would, however, be 5 false positives. At follow–up, new cases were identified illustrating the need for continued surveillance and re–screening at multiple time–points using developmentally sensitive instruments. Not all children who later receive a diagnosis are detectable during the toddler period.

Confidential: For Review Only

Research in Context

What is already known on this topic

In the UK, universal screening for autism is not recommended to facilitate early detection. There is limited research evidence of specific autism screening tools with longitudinal follow-up.

What this study adds

We examined the accuracy of the Quantitative Checklist for Autism in Toddlers (Q-CHAT) in a population cohort screened at 18-30 month and followed-up at age 4 years. The sensitivity and positive predictive value of the Q-CHAT were marginally higher than other screening tools, which supports other studies that have found it is not possible to identify all autistic children using a single instrument at a single time–point.

Confidential: For Review Only

Introduction

Autism affects approximately 1–2% of the population.[1] Screening requires a standardised and systematic approach to detect children who may require a diagnostic assessment. The costs and benefits of autism population screening need to be carefully balanced. Undue anxiety in false positives and the need for sufficient support services for those diagnosed should be offset by the many benefits of accurate screening that include earlier diagnosis, more timely access to specific interventions[2] and, better support for parents. In the UK, no autism specific screening instrument is recommended by the National Institute of Health and Care Excellence (NICE).[3] Autism is variable in onset and the significance over time of specific symptoms. No existing screening instrument, used at a single time–point, reaches a minimum of 80% sensitivity and specificity. In contrast, the American Academy of Pediatrics[4] recommends routine screening at well–child checks using the Checklist for Autism in Toddlers (CHAT)[5, 6] and the Modified CHAT (M–CHAT),[7] despite the limited research evidence for their use,[8] and insufficient evidence to determine if certain risk factors modify the performance characteristics of the tests.[9]

Lengthy delays between first concerns and an eventual diagnosis are often experienced.[10] There has been no evidence of a reduction in the age at diagnosis over the past decade in the UK.[11] Beliefs held by health professionals about screening for autism (e.g. system capacity, interventions available) may influence practice.[12] Equally, young autistic children may not present salient atypical behaviour in a short consultation to warrant an assessment referral. Atypical behaviours, such as Restricted and Repetitive Behaviours (RRBs) extend to the neurotypical child on a continuum[13] and spontaneously reduce with time.[14] The reduction of inappropriate referrals to already over–stretched services could serve to benefit children, their families and health services. 60% of referrals to a child development centre in the UK for possible autism resulted in an autism diagnosis. It is unclear what the outcome was for the remainder of the referrals.[15]

The CHAT[5] is a short parent–report questionnaire combined with a health professional observation. The CHAT was tested prospectively in 18 month old toddlers from the general population with diagnostic follow–up of those who scored above the cut–point to allow for the estimation of both sensitivity and specificity. At 18 months the CHAT identified 92% of autistic children when assessed and diagnosed by two independent psychiatrists using DSM–III–R criteria as the gold standard at that time.[6] By the 6–year follow–up, the sensitivity was

Confidential: For Review Only

38% indicating that many children did not present with sufficient features of autism to meet diagnostic criteria at 18 months. Nevertheless, the CHAT had very high specificity (98%) and was the first demonstration internationally that autism could be diagnosed as early as 18 months of age, when previously age–at–first diagnosis was typically not until later in childhood.[16] The CHAT was based on toddler developmental precursors of 'theory of mind',[17] such as joint attention[18-20] and pretend play,[21] so stemmed from a cognitive developmental theory about the nature of autism.[22]

The Modified Checklist for Autism in Toddlers (M–CHAT),[7] is a 23–item parent–report questionnaire, used in the United States healthcare system. It incorporates the original nine items from the CHAT, and includes additional items that may be characteristic of a young autistic child. Initial results reported sensitivity of 0·97, specificity of 0·99, and positive predictive value (PPV) of 0·68 when used with a telephone follow–up. Despite limitations to the initial studies[23] (e.g. referred and unselected samples were combined, and no systematic follow–up), the M–CHAT has been examined in a large population sample at 18–months.[24] Specificity was high (0·93), but sensitivity was low (0·34) and PPV was 0·35. The authors concluded that it may not be possible to detect all autistic children at 18–months. The M–CHAT has subsequently been revised (M–CHAT, Revised with Follow–Up (M–CHAT–R/F)),[25] consisting of 20 items, including examples to provide developmental context and clarity. This was tested in a large population (N=16,071), finding PPV to be 0·48, while another study[26] reported PPV to be 0·54. Sensitivity could not be determined in these two studies because only the screen positives were followed up.

The Quantitative Checklist for Autism in Toddlers (Q–CHAT)[27] was developed to dimensionalise autism in toddlerhood: it has been demonstrated that autistic traits can be measured quantitatively in the general population, using the Autism Spectrum Quotient (AQ) in adulthood,[28] the AQ–Adolescent,[29] the AQ–Child[30] and the Social–Responsiveness Scale (SRS),[31] in older children and adults. The Q–CHAT is a parent–report 25–item questionnaire that includes the CHAT and additional items. Each item is converted to a rating scale, thus quantifying autistic traits. This allows for the possibility of reporting behaviour at a reduced frequency. Q–CHAT domains include social communication, repetitive, stereotyped and sensory behaviours. A preliminary study found Q–CHAT discriminated well between young autistic children and unselected toddlers in the population at 18–24 months.[27] A short version of the Q–CHAT (Q–CHAT–10)[32] was developed and tested retrospectively on toddlers with

https://mc.manuscriptcentral.com/bmjpo
Confidential: For Review Only

an autism diagnosis compared to toddlers from the unselected population reported. Sensitivity and specificity were 0·91, and 0·89 respectively. However, these studies were not longitudinal, so cannot address the question of whether it is best to screen for autism at single or multiple time–points.

The goal of this study is to report a population screening study of nearly 4,000 toddlers at 18–30 months using the Q–CHAT with diagnostic assessments on a sub–sample of responders, and subsequent follow–up at four years. Screening was conducted using the child health surveillance database to identify the population eligible to participate. The study is reported in two phases, as undertaken. The aim of Phase 1 was to determine test accuracy of the Q–CHAT in the toddler period. The aim of Phase 2 was to identify autistic children who were not detected at Phase 1. We used the Childhood Autism Spectrum Test (CAST)[33] and the Checklist for Referral (CFR) to seek information on the child's history of developmental concerns. We conducted diagnostic assessments to identify any missed cases (false negatives) from Phase 1, as well as to confirm the diagnostic outcome of those already identified. This two–phase design allowed us to report sensitivity, specificity and PPV. Phase 1 was not evaluated at the time to ensure researchers remained blind to the results when undertaking Phase 2.

Methods

Patients and Public Involvement Statement

Patients or the public WERE NOT involved in the design, or conduct, or reporting, or dissemination plans of our research

Setting, Study Design and Procedure

Phase 1

All caregivers of infants between the ages of 18-30 months who were registered on the Child Health Surveillance Database at the specified Primary Care Trusts (PCT) on the date of mailing were invited to complete the Q-CHAT between March 2008 and April 2009. Screening was conducted via a postal questionnaire, distributed by the PCT. Luton questionnaires were mailed in March 2008. Bedfordshire questionnaires were mailed in May 2008. Cambridgeshire questionnaires were mailed in April 2009. Questionnaires were posted twice, two weeks apart, to maximise response. Returned questionnaires were scored by the research team and the sampling strategy was applied to establish the assessment sample.

https://mc.manuscriptcentral.com/bmjpo
Confidential: For Review Only

Research diagnostic assessments were arranged as soon as possible after completion of the Q-CHAT. All members of the assessment team remained blind to the child's Q-CHAT score. The Q-CHAT was re-administered prior to any of the assessment battery. Diagnostic outcome was defined by the researchers reaching a consensus diagnosis using all assessment data and researcher judgement.

Phase 1 Sampling Strategy

Sampling for assessment was weighted towards those with higher scores. Determination of the best cut-point for the Q-CHAT was one aim of the study and a sampling cut-point of 44 was chosen as the point at which 100% of children who scored ≥ 44 would be selected for assessment. The rationale for choosing 44 as the sampling cut-point was based on anticipated estimates of prevalence of autism (approximately 1%) balanced with the knowledge that over 70% of children with autism scored above 44 in our initial study[27], maximising sensitivity whilst generating a feasible number of assessments to be completed. To ensure that children with missing data were not missed from being included in a high scoring group, Q-CHAT items that were missing were allocated the maximum item score (four) and the total Q-CHAT score recalculated. Each Q-CHAT questionnaire therefore had two scores: the observed (assuming each item of missing data would score as not impaired) and the maximum (assuming each item of missing data would score as impaired). Observed and maximum Q-CHAT scores were grouped into four bands (≥44, 41-43, 38-40, ≤37). This ensured that missing data meant a child was more likely to be assessed.

The final sampling groups were determined according to the maximum score, taking into account the movement across score groups from observed score to maximum score. Sampling group allocation and randomisation took place prior to establishing whether or not the family had consented for further contact.

When the chance of being selected was less than 100%, selection for assessment was undertaken using random.org 8. 1% of children who had a maximum score ≤37 were selected for assessment to ensure that the assessment team were blind to whether there were likely to be concerns about the child at the time of assessment.

Phase 2

https://mc.manuscriptcentral.com/bmjpo
Confidential: For Review Only

All caregivers who consented to further contact following the Phase 1 screening (N=3,472) were invited to participate in Phase 2 at a minimum age of 48 months, between January and July 2011. Caregivers were sent the Childhood Autism Spectrum Test (CAST), a 37-item tool to identify children at risk for autism in children aged 4–11 years, in a non-clinical setting[33, 34], Parents were also sent a brief checklist enquiring about whether the child had ever been referred or diagnosed with any developmental and/or medical condition (Checklist for Referral, CFR).

Phase 2 Sampling

All children who scored ≥15 on the CAST (the recommended screening cut-point for epidemiological research)[33] were invited for a Phase 2 diagnostic assessment, along with all children whose caregiver indicated on the CFR that they had been referred for any of the following reasons: language delay/disorder, Hyperactivity/Attention Deficit Disorder (ADHD, ADD), dyspraxia, autism spectrum condition (autism), genetic/chromosomal abnormality, epilepsy, Tourette syndrome, and tuberous sclerosis. There was also a space on the CFR for the caregiver to provide more information about the reason for the referral and whether any diagnosis had been made. If the caregiver indicated that their child had been referred for any other reason not specified on the CFR, the research team decided on a case–by–case basis whether or not to invite the family for the assessment. All children who participated in Phase 1 assessments were invited for a Phase 2 assessment.

Diagnostic Outcome

In both Phase 1 and 2, diagnostic outcome was defined by the researchers reaching a consensus diagnosis using all assessment data and researcher judgement as autism or autism spectrum (if they met the International Statistical Classification of Diseases 10th Edition (ICD-10) criteria[35] for a pervasive developmental disorders diagnosis), atypical (if the child showed developmental concerns that were not related to autism) or typical (if there were no developmental concerns about the child). It was not possible for all of the assessment team to remain blind to the diagnostic outcome from the Phase 1 assessments at Phase 2. However, the assessment team re-examined blind to the Q-CHAT score throughout Phase 2 assessments.

https://mc.manuscriptcentral.com/bmjpo

Ethical approval was granted by the Cambridge Local Research Ethics Committee (05/Q0108/53). The University of Cambridge was the sponsor of this research. Informed consent was obtained from all caregivers included in the study.

Measures

Q-CHAT

The Q-CHAT has a forced choice design, with five possible responses for each item (with the exception of item 4, see below). For each item, response representing the most 'autistic' symptomatology scores four points and the least 'autistic' response scores zero. On approximately half the items (items 3, 7, 8, 11, 12, 13, 16, 18, 20, 22, 23, 24, 25) the 'autistic' response is the positive one. On the other half, (items 1, 2, 4, 5, 6, 9, 10, 14, 15, 17, 19, 21) the 'autistic' response is the negative one. Item 4 is concerned with speech and includes a sixth option of 'my child does not speak', also scoring four points. Total Q-CHAT score is obtained by summing the score on each item, giving a maximum of 100. Each of the 25 questions is accompanied by a colour illustration to attempt to increase both response and comprehensibility of the items.

Demographic Data

Data collected about the child included age, sex, birth order, ethnicity and multiple birth status. Data collected about the caregivers included Socio-Economic Status (SES) and parental educational attainment. SES was derived at person level rather than household level, using the five class system of the National Statistics-Socio Economic Classification (NS-SEC)[36]. Parental educational attainment was assessed by the highest level of qualification of each parent.

Diagnostic Assessment

The ADOS-G[37] was administered. The ADOS-G is a semi-structured, standardized assessment of social interaction, communication, play, and imagination. It is a reliable and valid measure for the diagnosis of autism relative to those with non-autistic conditions. Module 1 was chosen at Phase 1 since it was not expected that many children would have expressive language abilities beyond basic phrase speech. Module 2 or 3 was chosen at Phase 2. ADOS-G assessments were videotaped, but item scoring was conducted independently by two members of the assessment team and consensus codes were agreed as soon as possible after the assessment. The assessment team comprised of at least one very experienced

Confidential: For Review Only

research psychologist alongside a research assistant who also had been trained to reliability standards.

The Autism Diagnostic Interview-Revised (ADI-R)[38] was administered with the caregiver. The ADI-R is a diagnostic semi-structured interview that generates an algorithm score based on behaviours in three domains: social communication, social interaction and repetitive and stereotyped behaviours and interests. The interview also enquires about age of onset of the symptoms. Due to the practicalities in arranging the assessments, it was not possible to counterbalance administration of the ADOS-G and ADI-R. However, the ADI-R and ADOS-G was never carried out by the same researcher to reduce the possibility of experimenter expectation bias. All ADI-R assessments were audio-taped.

The Mullen Scales of Early Learning (MSEL)[39] is a standardised measure of cognitive, expressive and receptive language, motor and perceptual abilities. The MSEL was usually carried out while the other researcher was administering the ADI-R with the caregiver. The gross motor scale was not completed as this does not contribute to the overall Early Learning Composite (ELC). The Vineland Adaptive Behavior Scale (VABS)[40] is a standardised measure of personal and social skills. The interview version was administered to the parent. There are four domains that measure communication, daily living skills, socialisation and motor skills, as well as an optional maladaptive domain (not administered here). The VABS was usually administered at the end of the testing session.

Confidential: For Review Only

Table 1: Phase 1 sampling strategy, with study numbers

Sampling Group	Observed Q-CHAT (missing items = 0)	Maximum Q-CHAT (missing items = 4)	% sampled	Total	Assessment numbers		
					Selected	Invited	Agreed
1	≥38	≥44	100%	86	86	74	40
2	≤37	≥44	50%	21	10	9	6
3	41–43	41–43	50%	61	31	28	16
4	≤40	41–43	50%	21	10	8	5
5	38–40	38–40	25%	129	31	28	20
6	≤37	38–40	25%	25	5	5	2
7	≤37	≤37	1%	3300	40	39	32
**Total**				**3643**	**213**	**191**	**121**

Confidential: For Review Only

Statistical methods

Due to the stratified sample all analyses were adjusted for the probability of being selected into the assessment subsample at each Phase via inverse probability weighting. This takes account of the differences in sampling proportions and the selective participation in the assessment across the sampling groups. A sensitivity analysis also included within the weights are the health trust non–response rates at each stage. A receiver operator characteristic (ROC) area under the curve (AUC) analysis assessed the discriminant power of the Q–CHAT and CAST and CFR in distinguishing autism cases from non–autism cases (defined as typical and atypical) and from autism and all other atypical development from typically developing children, across all score values. Confidence intervals were obtained from robust standard errors. All analysis was undertaken in Stata 14.[41] A series of sensitivity analyses to decisions on the screening instrument were undertaken (see Table 2).

Role of the funding source

The funding source had no role in the data collection, data analysis, data interpretation, or writing of the report. The corresponding author had full access to all the data in the study and had final responsibility for the decision to submit for publication.

Results

Phase 1

3,770 Q–CHAT questionnaires were returned 436 (14% response) from Luton, 1,256 (25% response) from Bedfordshire and 2,078 (42% response) from Cambridgeshire. 223 were selected for assessment, 32 did not give consent to be contacted, therefore 191 were invited and 121 competed the assessments. See Table 1 for Phase 1 sampling strategy with study numbers, Supplementary Figure 1 for the flow chart from phase 1 and Supplementary Table 1 for characteristics of children and their families throughout the study, supplementary Figure 2 shows the distribution of Q–CHAT with sampling bands indicated.

Following assessment, 11 children were defined as autistic, 16 as atypical (7 language delay, 5 developmental delay, 4 other atypical), and 94 as typical. At a cut–point of ≥39, the positive predictive value (PPV) for autism was 17% (95%CI 8%–31%) (Table 2).

Confidential: For Review Only

Autism	TP	FN	FP	TN	Sensitivity	Specificity	PPV	NPV	ROC
**Main analysis – phase 1**									
Q–CHAT 39+	36	0	179	3428	1.00 (0.72–1.0)*	0.95 (0.92–0.97)	0.17 (0.08–0.31)	1.00 (0.93–1.0)*	0.98 (0.96–1.00)
**Main analysis – phase 2**									
**Q–CHAT 39+**	**27**	**34**	**69**	**3513**	**0.44 (0.26–0.64)**	**0.98 (0.97–0.99)**	**0.28 (0.15–0.46)**	**0.99 (0.98–0.99)**	**0.86 (0.78–0.94)**
CAST ≥15+ / CFR positive	50	7	98	3488	0.88 (0.63–0.97)	0.97 (0.95–0.98)	0.34 (0.23–0.46)	1.00 (0.99–1.00)	0.99 (0.98–1.00)
**Sensitivity analysis – phase 1**									
Q–CHAT 31+	36	0	1104	2504	1.00 (0.72–1.0)*	0.69 (0.53–0.82)	0.03 (0.01–0.07)	1.00 (0.88–1.0)*	Same as for 39+
Q–CHAT 38+	36	0	238	3369	1.00 (0.71–1.0)*	0.93 (0.90–0.96)	0.13 (0.06–0.25)	1.00 (0.92–1.0)*	Same as for 39+
Q–CHAT 39+ with parental concern	30	6	73	3534	0.84 (0.43–0.97)	0.98 (0.97–0.99)	0.29 (0.13–0.52)	1.00 (0.99–1.00)	1.00 (0.99–1.00)
Q–CHAT 39+ adjust initial non–response	49	0	201	3393	1.00 (0.72–1.0*)	0.94 (0.91–0.97)	0.20 (0.09–0.37)	1.00 (0.93–1.0)*	0.98 (0.96–1.00)
**Sensitivity analysis – phase 2**									
Q–CHAT 31+	48	13	589	2993	0.79 (0.64–0.89)	0.84 (0.65–0.93)	0.08 (0.03–0.19)	1.00 (0.99–1.00)	Same as for 39+
Q–CHAT 38+	35	26	92	3489	0.57 (0.39–0.74)	0.97 (0.96–0.99)	0.28 (0.16–0.43)	0.99 (0.99–1.00)	Same as for 39+
Q–CHAT 39+ with parental concern	24	37	29	3553	0.40 (0.23–0.60)	0.99 (0.98–1.00)	0.46 (0.24–0.69)	0.99 (0.98–0.99)	0.90 (0.84–0.96)
Q–CHAT 39+ adjust initial non–response	38	35	73	3497	0.51 (0.30–0.72)	0.98 (0.96–0.99)	0.34 (0.17–0.55)	0.99 (0.98–0.99)	0.89 (0.81–0.96)
CAST ≥15	32	25	8	3578	0.56 (0.37–0.74)	1.00 (0.99–1.00)	0.81 (0.59–0.92)	0.99 (0.99–1.00)	0.94 (0.88–0.99)
Q–CHAT 39+ include parent reported cases	30	34	67	3512	0.47 (0.30–0.65)	0.98 (0.97–0.99)	0.31 (0.52–0.82)	0.99 (0.98–0.99)	0.86 (0.79–0.94)

Table 2: Autism diagnosis results Phase 1 and Phase 2

Confidential: For Review Only

Phase 2

3,472 consenting caregivers from Phase 1 were sent the Phase 2 study materials. 2,005 returned both the CFR and CAST (58% response), a further 6 returned just the CAST and 5 returned just the CFR. 42 children who had an assessment in Phase 1 did not return the CAST or CFR.

172 caregivers reported developmental concerns on the CFR and 43 children scored 15 and above on the CAST (not in Phase 1 assessment sample) and were selected for assessment. 81 children who had been assessed in Phase 1 completed the assessment (1 child did not complete all the assessments and is therefore not included in the analysis). In total, 158 children completed the assessment at Phase 2, 23 with CFR concerns and CAST score ≥ 15, 63 with CFR concerns and CAST scores ≤ 15, 6 with CAST scores of ≥ 15 with no CFR concerns, 56 who scored ≤ 15 on the CAST and who did not report any developmental concerns on the CFR but who were assessed in Phase 1, and 10 children who were assessed in Phase 1 but who did not return the CFR or CAST (Figure 1 shows assessment flow and diagnostic outcome, with Figure 2 showing the characteristics of the two screening tests). 11 children were considered as possibly autistic (prevalence of 0·98%, 95%CI 0·45%–2·16%).

81 children were assessed at both Phase 1 and 2. Of the 9 children whose diagnostic outcome at Phase 1 was autism who took part in Phase 2, all were still classified as autism. A further 6 children were now classified as autism (4 who were typical at Phase 1, and 2 who were atypical). 77 children were assessed for the first time at Phase 2. Of these 29 were classified as autism, 24 as atypical development and 24 as typically developing, giving a prevalence of autism of 1·57% (95%CI 0·90%–2·74%). Figure 3 shows the study flow from Phase 1 to Phase 2.

https://mc.manuscriptcentral.com/bmjpo

Confidential: For Review Only

Insert Figure 1 here

Using the CAST alone as the screen at Phase 2, 1,994 children had complete data. At a cut–point of ≥15, sensitivity was 56% (95%CI 37%–74%) specificity was 100% (95%CI 99%–100%), and PPV was 81% (95%CI 59%–92%) (Table 2). The area under the ROC curve was 0·94 (95%CI 0·88–0·99) (Supplementary Figure 3). Adding in the CFR selection criteria improved sensitivity to 88% (95%CI 63%–97%) but decreased specificity slightly to 97% (95%CI 95%–98%), with a PPV of 34% (95%CI 23%–46%) and an area under the ROC curve of 0·99 (95%CI 0·98–1·00).

At a cut–point of ≥39, the sensitivity of the Q–CHAT to predict Phase 2 outcome (autism vs atypical and/or typical) was 44% (95%CI 26%–64%), specificity was 98% (95%CI 97%–99%), and PPV was 28% (95%CI 15%–46%) (Table 2). The area under the ROC curve was 0·86 (95%CI 0·78–0·94).

Sensitivity analyses: Optimising the Q–CHAT cut–point for Phase 2 outcome found two additional cut–points, Q–CHAT 31+ maximized sensitivity and specificity at the cost of false positives and using Q–CHAT 38+ marginally improved sensitivity with little additional benefit. Other sensitivity analyses at Phase 1 and Phase 2 are shown in Table 2. See Figure 2 for characteristics and comparison of the screening instruments.

Insert Figure 2 here

Insert Figure 3 here

Discussion

At Phase 1 outcome, all children who were classified as autistic failed the Q–CHAT at a cut–point of 39, demonstrating that early detection and diagnosis of autism is possible. The PPV of the Q–CHAT for autism was 17%. Misinterpretation of behaviours that are not well–established as well as developmental immaturity may contribute to low PPV. In Phase 2 we identified autistic children at age 4 who were not identified by the Q–CHAT. At a cut–point of ≥39, the sensitivity of the Q–CHAT to predict autism was 44% (95%CI 26%–64%), specificity was 98% (95%CI 97%–99%), and PPV was 28% (95%CI 15%–46%). This demonstrates that the Q–CHAT alone cannot be used to detect all autistic children. This might reflect the Q–CHAT properties, but equally it likely reflects a subgroup of autistic

Confidential: For Review Only

children who do not show symptoms of sufficient 'severity' until later in childhood, and/or that symptoms of autism change with age, so require developmentally sensitive instruments at different ages. The CAST picked up many of the cases of autism that were missed by the Q–CHAT, demonstrating the need for repeat screening rather than relying on a single instrument at one time point.. However, the sensitivity of the CAST at a cut point of 15 was only moderate (56%), compared to our earlier study showing sensitivity of 100%.[33]

A strength of this study was the prospective, population–based design that allowed us to assess the Q–CHAT accurately at two time–points. Our approach to missing data and subsequent sampling strategy was extremely cautious and conservative, and ensured maximum capture of potential cases in the assessment phase, maximising sensitivity. However, studies such as the current one are challenged by modest participation rates, selection bias and attrition over time. Despite adopting strategies to attempt to increase response, overall it was low, but not unusual for an unsolicited postal survey. Whether or not those who responded initially to the Q–CHAT (which determined the population for the rest of the study) were biased towards those with concerns (either justified or the 'worried well') or in the opposite direction remains largely unknown. A second limitation is that in Phase 1, only 1% of children who scored ≤37 were sampled. This proportion was chosen for pragmatic reasons in relation to feasibility (i.e. to keep the number of lengthy diagnostic assessments in children with no developmental concerns to a minimum, and to reduce expectation bias in the research team). None of the sampled children with scores ≤37 met our assessment case definition. It is important to underline that these results are based purely on outcome at Phase 1 and do not reflect the overall test accuracy statistics from Phase 2.

Few studies attempt population screening, partly because they are very time consuming and expensive. Younger toddlers have mild or transient developmental delays that resolve, resulting in low PPV, as was found in another study.[42] Introducing a follow–up interview (such as used with the M–CHAT[23, 25, 26]) for those who screen positive may reduce the number of unnecessary referrals for a diagnostic assessment, especially with children older than 20 months.[43] Nevertheless, a recent meta–analysis of M–CHAT studies found a lack of evidence on its performance in low–risk children or at age 18 months.[44] A universal, primary care-based screening study was conducted using the Modified Checklist for Autism in Toddlers with Follow-Up (M-CHAT/F) in the United States. Overall, the sensitivity was 38.8%, and PPV was 14.6%. Sensitivity was higher in older toddlers and with repeated

Confidential: For Review Only

screenings, whereas PPV was lower in girls[45]. This study was the first using the M-CHAT to follow up children screened in the population, thus allowing for an accurate estimate of the sensitivity of the instrument. Since the publication of the original CHAT[5, 6, 16] no study in the UK to date has achieved screening data on such a large population sample of toddlers and accurately determined sensitivity through follow up.

Overall, this study demonstrates that (1) that the Q–CHAT detected every child who received an autism diagnosis at Phase 1 outcome and therefore it is possible to detect autism at 18–30 months; and (2) it is not possible to detect every child at a very young age who will later be diagnosed as autistic. The Q–CHAT can be used to screen toddlers at 18–30 months for autism to enable accelerated referral into the diagnostic pathway. However, the PPV is low, thus potentially generating a high number of children who may not have autism, but who may still require a developmental assessment to determine whether they warrant a referral for intervention and support. Outcome data from Phase 2 revealed many autistic children that the Q–CHAT did not identify. The autism spectrum is so broad that it is not reasonable to expect that every child will be detectable as a young toddler. Therefore, when looking at the Q–CHAT longitudinally, it is evident that screening at a single time–point results in less than optimal test properties. We suspect that the missed cases reflects a subgroup of autistic whose symptoms are not severe enough to warrant a diagnosis during toddlerhood, and that the symptoms of autism change with age. We therefore recommend continued surveillance and re–screening at multiple time–points using developmentally sensitive instruments in order to identify a higher proportion of cases for maximal public health impact. This study demonstrates that no single instrument will detect all cases of autism at a single time point. Therefore, continued monitoring throughout the life course is necessary in order to identify when autistic traits begin to impair an individual's life. High quality evidence from random controlled trials is needed to determine whether early detection and consequent early implementation of specific interventions is able to change outcomes in autistic toddlers.

Total Q–CHAT score was used to determine which sampling band each child fell into. A sum (total) score based on equal weighting of all items may not the most appropriate method by which to quantify an individual's autistic traits. Other work by our group has shown that there are at least two items on the Q–CHAT whereby the prevalence of endorsement of the trait in the autistic direction is lower for toddlers with a diagnosis of autism, compared to population controls (items 8 and 18).[32] Item response analysis will be conducted to determine whether

Confidential: For Review Only

every item equally contributes useful information about the underlying latent dimensions. Defining a threshold on each factor that can be measured quantitatively (continuous approach) may be a more appropriate scoring method. Furthermore, exploration of different age–specific scoring algorithms should be implemented to determine which variables carry greatest relative importance to optimise screening results.[2, 43]

Word Count: 5571

Figure 1: Assessment flow and diagnostic outcome
Figure 2: Characteristics and comparison of the screening instruments (not weighted)
Figure 3: Study flow from Phase 1 to Phase 2

Funding: The study was funded by the National Lottery Community Fund, the Autism Research Trust, and was conducted in association with the Gillings Family Charitable Trust and the National Institute for Health Research (NIHR) Collaboration for Leadership in Applied Health Research and Care (CLAHRC) East of England at Cambridgeshire and Peterborough NHS Foundation Trust, and by the NIHR Cambridge Biomedical Research Centre. The views expressed are those of the author(s) and not necessarily those of the NHS, the NIHR or the Department of Health. SBC and TC were supported by the Medical Research Council UK and the Innovative Medicines Initiative (IMI) 2 Joint Undertaking (JU) under grant agreement no. 777394. FEM was supported by MRC U105292687. The JU receives support from the European Union's Horizon 2020 research and innovation programme and EFPIA and Autism Speaks, Autistica, and SFARI. SBC and CA were also supported by the Templeton World Charitable Foundation and the Wellcome Trust during the period of this work. Grant numbers not applicable.

Acknowledgements: We are grateful to the families who participated in all phases of the study. We are also grateful to staff at Luton, Bedfordshire and Cambridgeshire CCGs for their cooperation in defining the target population and mailing the questionnaires. We are grateful to Gina Gomez, Kristelle Hudry, Sally Clifford, Georgina Woods, Emma Robson, Philippa Lewington, Susan Sadek, Kimberly Armstrong, Martine Roelfsema and Kim Davies for help with data collection.

Author Contribution: CA, FM, CB, TC and SB–C contributed to the study design. CA, LR, GP and RS collected the data. CA and FEM did the data analysis and FEM is guarantor of the analysis.

Confidential: For Review Only

All authors contributed to data interpretation and writing of the report.

Competing Interests: Dr. Charman has received research grant support from the Medical Research Council (UK), the National Institute for Health Research, Horizon 2020 and the Innovative Medicines Initiative (European Commission), MQ, Autistica, FP7 (European Commission), the Charles Hawkins Fund, and the Waterloo Foundation. He has served as a consultant to F. Hoffmann–La Roche Ltd. He has received royalties from Sage Publications and Guilford Publications. All other authors report no biomedical financial interests or potential conflicts of interest.

Transparency declaration: The lead author and guarantor affirms that the manuscript is an honest, accurate, and transparent account of the study being reported; that no important aspects of the study have been omitted; and that any discrepancies from the study as planned and registered have been explained.

Dissemination to participants and related patient and public communities: The authors plan to send a summary of the results to participating NHS Trusts. A lay summary of the results will be available on the Autism Research Centre's website. Results will be presented at international conferences.

Patient and public involvement: Patients or the public were not involved in the design, or conduct, or reporting, or dissemination plans of our research.

Data sharing: At the time of data collection, consent was not obtained from participants for the purposes of data sharing. Therefore even anonymised data are not available.

Confidential: For Review Only

References

1. Baron-Cohen S, Scott FJ, Allison C, Williams J, Bolton P, Matthews FE, et al. Prevalence of autism-spectrum conditions: UK school-based population study. Br J Psychiatry. 2009;194(6):500-9.
2. Zwaigenbaum L, Bauman ML, Fein D, Pierce K, Buie T, Davis PA, et al. Early Screening of Autism Spectrum Disorder: Recommendations for Practice and Research. Pediatrics. 2015;136 Suppl 1:S41-59.
3. National Institute for Health and Clinical Excellence. Autism: recognition, referral and diagnosis of children and young people on the autism spectrum. (Clinical guideline 128). https://www.nice.org.uk/guidance/cg128; 2011.
4. Johnson CP, Myers SM. Identification and Evaluation of Children With Autism Spectrum Disorders. Pediatrics. 2007;120(5):1183-215.
5. Baron-Cohen S, Allen J, Gillberg C. Can autism be detected at 18 months? The needle, the haystack, and the CHAT. Br J Psychiatry. 1992;161:839-43.
6. Baron-Cohen S, Cox A, Baird G, Swettenham J, Nightingale N, Morgan K, et al. Psychological markers in the detection of autism in infancy in a large population. Br J Psychiatry. 1996;168(2):158-63.
7. Robins DL, Fein D, Barton ML, Green JA. The Modified Checklist for Autism in Toddlers: an initial study investigating the early detection of autism and pervasive developmental disorders. J Autism Dev Disord. 2001;31(2):131-44.
8. Al-Qabandi M, Gorter JW, Rosenbaum P. Early autism detection: are we ready for routine screening? Pediatrics. 2011;128(1):e211-7.
9. Siu AL, and the U. S. Preventive Services Task Force. Screening for autism spectrum disorder in young children: Us preventive services task force recommendation statement. JAMA. 2016;315(7):691-6.
10. Crane L, Chester JW, Goddard L, Henry LA, Hill E. Experiences of autism diagnosis: A survey of over 1000 parents in the United Kingdom. Autism. 2016;20(2):153-62.
11. Brett D, Warnell F, McConachie H, Parr JR. Factors Affecting Age at ASD Diagnosis in UK: No Evidence that Diagnosis Age has Decreased Between 2004 and 2014. J Autism Dev Disord. 2016;46(6):1974-84.
12. Zwaigenbaum L, Bauman ML, Choueiri R, Kasari C, Carter A, Granpeesheh D, et al. Early Intervention for Children With Autism Spectrum Disorder Under 3 Years of Age: Recommendations for Practice and Research. Pediatrics. 2015;136 Suppl 1:S60-81.
13. Leekam S, Tandos J, McConachie H, Meins E, Parkinson K, Wright C, et al. Repetitive behaviours in typically developing 2-year-olds. J Child Psychol Psychiatry. 2007;48(11):1131-8.
14. Leekam SR, Prior MR, Uljarevic M. Restricted and repetitive behaviors in autism spectrum disorders: a review of research in the last decade. Psychol Bull. 2011;137(4):562-93.
15. Williams AN, Mold B, Kilbey L, Naganna P. Forty years of referrals and outcomes to a UK Child Development Centre (CDC): Has demand plateaued? Child Care Health Dev. 2018.
16. Baird G, Charman T, Baron-Cohen S, Cox A, Swettenham J, Wheelwright S, et al. A screening instrument for autism at 18 months of age: a 6-year follow-up study. J Am Acad Child Adolesc Psychiatry. 2000;39(6):694-702.
17. Baron-Cohen S. Autism: A specific cognitive disorder of "mind-blindness.". International Review of Psychiatry. 1990;2(1):81-90.
18. Baron-Cohen S. Perceptual role taking and protodeclarative pointing in autism. British Journal of Developmental Psychology. 1989;7(2):113-27.

https://mc.manuscriptcentral.com/bmjpo
Confidential: For Review Only

19. Baron-Cohen S. Joint-attention deficits in autism: Towards a cognitive analysis. Development and Psychopathology. 1989;1(3):185-9.
20. Goodhart F, Baron-Cohen S. How many ways can the point be made? Evidence from children with and without autism. First Language. 1993;13(38, Pt 2):225-33.
21. Baron-Cohen S. Autism and symbolic play. British Journal of Developmental Psychology. 1987;5(2):139-48.
22. Charman T, Baron-Cohen S, Swettenham J, Baird G, Cox A, Drew A. Testing joint attention, imitation, and play as infancy precursors to language and theory of mind. Cognitive Development. 2000;15(4):481-98.
23. Kleinman JM, Robins DL, Ventola PE, Pandey J, Boorstein HC, Esser EL, et al. The modified checklist for autism in toddlers: a follow-up study investigating the early detection of autism spectrum disorders. J Autism Dev Disord. 2008;38(5):827-39.
24. Stenberg N, Bresnahan M, Gunnes N, Hirtz D, Hornig M, Lie KK, et al. Identifying children with autism spectrum disorder at 18 months in a general population sample. Paediatr Perinat Epidemiol. 2014;28(3):255-62.
25. Robins DL, Casagrande K, Barton M, Chen CM, Dumont-Mathieu T, Fein D. Validation of the modified checklist for Autism in toddlers, revised with follow-up (M-CHAT-R/F). Pediatrics. 2014;133(1):37-45.
26. Chlebowski C, Robins DL, Barton ML, Fein D. Large-scale use of the modified checklist for autism in low-risk toddlers. Pediatrics. 2013;131(4):e1121-7.
27. Allison C, Baron-Cohen S, Wheelwright S, Charman T, Richler J, Pasco G, et al. The Q-CHAT (Quantitative CHecklist for Autism in Toddlers): a normally distributed quantitative measure of autistic traits at 18-24 months of age: preliminary report. J Autism Dev Disord. 2008;38(8):1414-25.
28. Baron-Cohen S, Wheelwright S, Skinner R, Martin J, Clubley E. The autism-spectrum quotient (AQ): evidence from Asperger syndrome/high-functioning autism, males and females, scientists and mathematicians. J Autism Dev Disord. 2001;31(1):5-17.
29. Baron-Cohen S, Hoekstra RA, Knickmeyer R, Wheelwright S. The Autism-Spectrum Quotient (AQ)--adolescent version. J Autism Dev Disord. 2006;36(3):343-50.
30. Auyeung B, Baron-Cohen S, Wheelwright S, Allison C. The Autism Spectrum Quotient: Children's Version (AQ-Child). J Autism Dev Disord. 2008;38(7):1230-40.
31. Constantino JN, Davis SA, Todd RD, Schindler MK, Gross MM, Brophy SL, et al. Validation of a brief quantitative measure of autistic traits: comparison of the social responsiveness scale with the autism diagnostic interview-revised. J Autism Dev Disord. 2003;33(4):427-33.
32. Allison C, Auyeung B, Baron-Cohen S. Toward brief "Red Flags" for autism screening: The Short Autism Spectrum Quotient and the Short Quantitative Checklist for Autism in toddlers in 1,000 cases and 3,000 controls [corrected]. J Am Acad Child Adolesc Psychiatry. 2012;51(2):202-12 e7.
33. Williams J, Scott F, Stott C, Allison C, Bolton P, Baron-Cohen S, et al. The CAST (Childhood Asperger Syndrome Test): test accuracy. Autism. 2005;9(1):45-68.
34. Scott FJ, Baron-Cohen S, Bolton P, Brayne C. The CAST (Childhood Asperger Syndrome Test): preliminary development of a UK screen for mainstream primary-school-age children. Autism. 2002;6(1):9-31.
35. World Health Organization. The ICD-10 Classification of Mental and Behavioural Disorders: Diagnostic Criteria for Research. Geneva: WHO; 1993.
36. Statistics. OfN. Self–coded version of the NS–SEC 2008. Available from: http://www.ons.gov.uk/about–statistics/classifications/current/ns–sec/self–coded/index.html.
37. Lord C, Risi S, Lambrecht L, Cook EH, Jr., Leventhal BL, DiLavore PC, et al. The autism diagnostic observation schedule-generic: a standard measure of social and

Confidential: For Review Only

communication deficits associated with the spectrum of autism. J Autism Dev Disord. 2000;30(3):205-23.
38. Lord C, Rutter M, Le Couteur A. Autism Diagnostic Interview-Revised: a revised version of a diagnostic interview for caregivers of individuals with possible pervasive developmental disorders. J Autism Dev Disord. 1994;24(5):659-85.
39. Mullen EM. Mullen Scales of Early Learning manual. AGS ed. Circle Pines, MN: American Guidance Service; 1995. ix, 85 p. p.
40. Sparrow SS, Balla DA, Cicchetti DV. Vineland adaptive behavior scales: Survey forms manual: AGS Publ.; 2005.
41. StataCorp. Stata Statistical Software: Release 14. College Station, TX: StataCorp LP; 2015.
42. Pandey J, Verbalis A, Robins DL, Boorstein H, Klin AM, Babitz T, et al. Screening for autism in older and younger toddlers with the Modified Checklist for Autism in Toddlers. Autism. 2008;12(5):513-35.
43. Sturner R, Howard B, Bergmann P, Morrel T, Landa R, Walton K, et al. Accurate Autism Screening at the 18-Month Well-Child Visit Requires Different Strategies than at 24 Months. J Autism Dev Disord. 2017.
44. Yuen T, Penner M, Carter MT, Szatmari P, Ungar WJ. Assessing the accuracy of the Modified Checklist for Autism in Toddlers: a systematic review and meta-analysis. Dev Med Child Neurol. 2018.
45. Guthrie W, Wallis K, Bennett A, Brooks E, Dudley J, Gerdes M, et al. Accuracy of Autism Screening in a Large Pediatric Network. Pediatrics. 2019;144(4).

Confidential: For Review Only

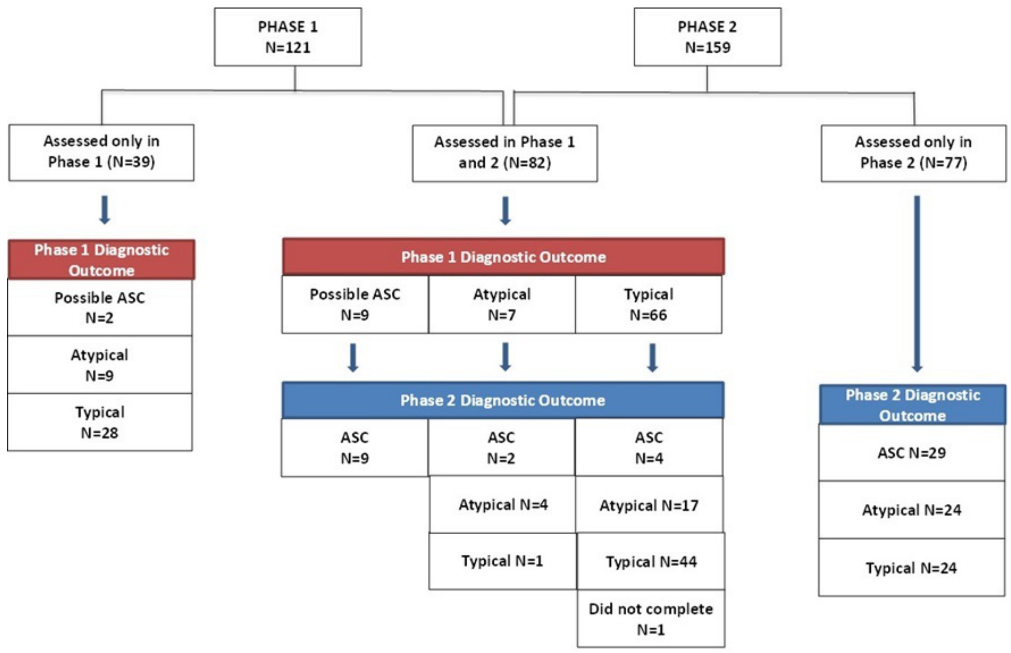
PHASE 1
N=121
PHASE 2
N=159
Assessed only in Phase 1 (N=39)
Assessed in Phase 1 and 2 (N=82)
Assessed only in Phase 2 (N=77)
Phase 1 Diagnostic Outcome
Possible ASC N=2
Atypical N=9
Typical N=28
Phase 1 Diagnostic Outcome
Possible ASC N=9
Atypical N=7
Typical N=66
Phase 2 Diagnostic Outcome
ASC N=9
ASC N=2
ASC N=4
Atypical N=4
Atypical N=17
Typical N=1
Typical N=44
Did not complete N=1
Phase 2 Diagnostic Outcome
ASC N=29
Atypical N=24
Typical N=24

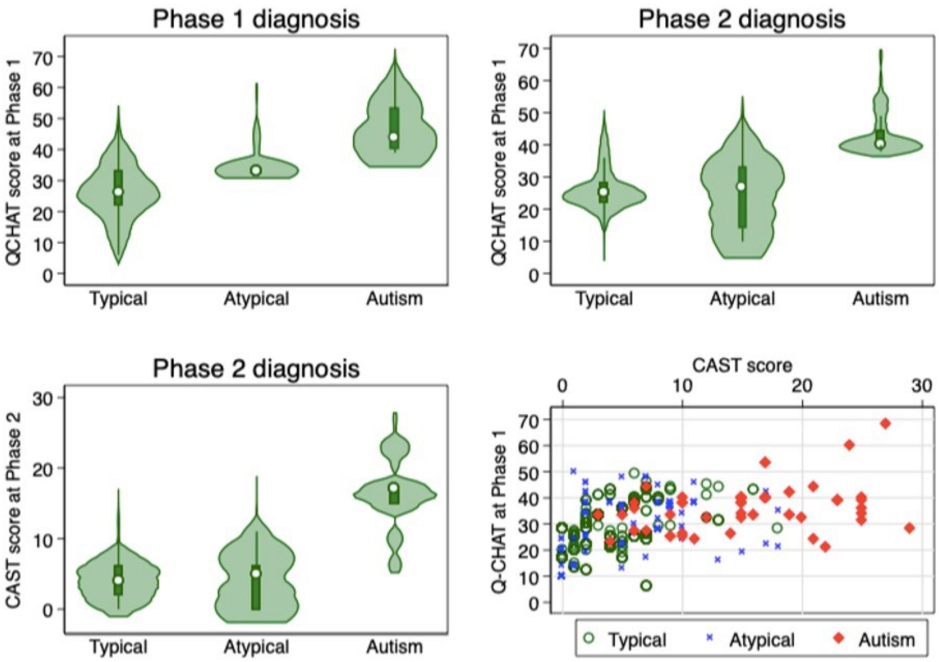
Phase 1 diagnosis
QCHAT score at Phase 1
Typical
Atypical
Autism
Phase 2 diagnosis
QCHAT score at Phase 1
Typical
Atypical
Autism
Phase 2 diagnosis
CAST score at Phase 2
Typical
Atypical
Autism
CAST score
Q-CHAT at Phase 1
Typical
Atypical
Autism

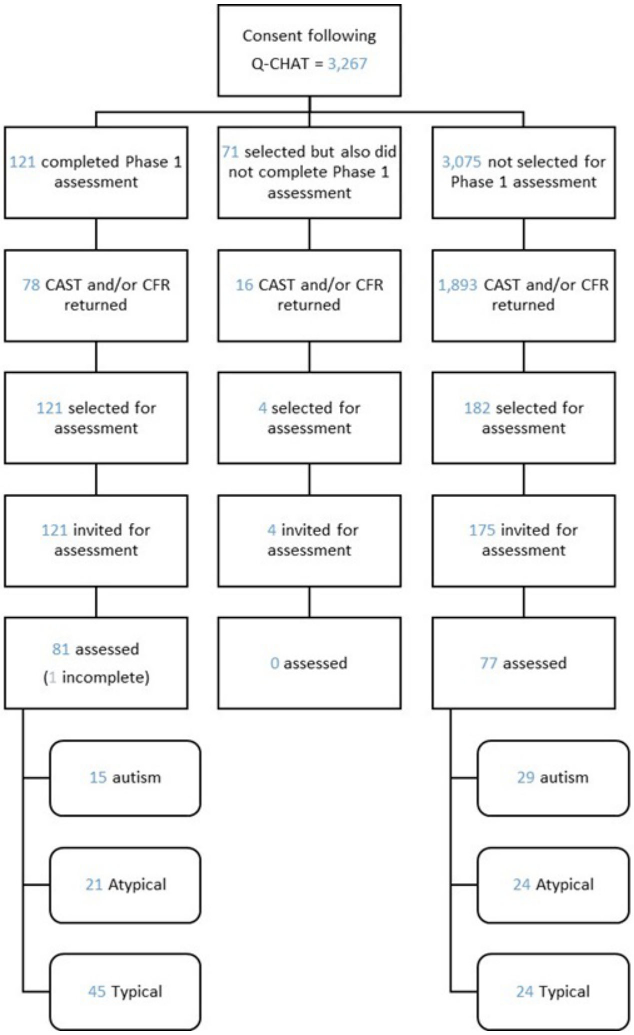
Consent following Q-CHAT = 3,267
121 completed Phase 1 assessment
71 selected but also did not complete Phase 1 assessment
3,075 not selected for Phase 1 assessment
78 CAST and/or CFR returned
16 CAST and/or CFR returned
1,893 CAST and/or CFR returned
121 selected for assessment
4 selected for assessment
182 selected for assessment
121 invited for assessment
4 invited for assessment
175 invited for assessment
81 assessed (1 incomplete)
0 assessed
77 assessed
15 autism
21 Atypical
45 Typical
29 autism
24 Atypical
24 Typical

Supplementary Appendix

Supplementary methods

Sensitivity analyses

Analysis was undertaken to maximise the sensitivity and specificity using a range of cut points across the Q-CHAT distribution. Additional cut points that also maximised sensitivity and specificity but had a larger number of false positives are provided. In addition a-priori a measure of parental concern taken at the same time as the screening test was included to investigate whether it improved the screening properties. Additionally, an analysis for initial non-response was undertaken to show the results were robust to the initial missing data.

Confidential: For Review Only

Supplementary Table 1: Study Characteristics

		Phase I				Phase II			
		Total		Assessment		Total		Assessment	
PCT	Luton	416	(11%)	22	(18%)	176	(9%)	19	(12%)
	Bedfordshire	1217	(33%)	39	(32%)	638	(32%)	55	(35%)
	Cambridgeshire	2018	(55%)	60	(50%)	1183	(59%)	84	(53%)
Child sex	Female	1759	(48%)	42	(35%)	972	(49%)	56	(35%)
	Male	1884	(52%)	79	(65%)	1025	(51%)	102	(65%)
	Unknown*	70		0		1		0	
Age child at Phase I	16–19 months	251	(7%)	15	(12%)	142	(7%)	13	(8%)
	20–24 months	1478	(41%)	49	(40%)	831	(42%)	69	(44%)
	25–29 months	1474	(40%)	46	(38%)	814	(41%)	66	(42%)
	30–34 months	440	(12%)	11	(9%)	210	(11%)	10	(6%)
	Unknown*	125		0		6		0	

* Children with unknown sex or age were excluded from the analysis and not included within the rest of the table
+ General Certificate of Secondary Education

Confidential: For Review Only

		Phase I				Phase II			
		Total		Assessment		Total		Assessment	
Mothers age at birth (Median, IQR)		32	[28,35]	31	[27,36]	33	[29,36]	32	[29,36]
Mothers education	None / <5 GCSE[+]	480	(13%)	25	(21%)	166	(8%)	19	(12%)
	5 GCSE	548	(15%)	21	(17%)	252	(13%)	24	(15%)
	2+ A levels	512	(14%)	11	(9%)	255	(13%)	15	(9%)
	Degree	1684	(45%)	48	(40%)	1068	(53%)	80	(51%)
	Unknown	546	(14%)	16	(13%)	267	(13%)	20	(13%)
Mother occupation	Not working	1545	(41%)	58	(48%)	736	(37%)	67	(42%)
	Student	86	(2%)	4	(3%)	40	(2%)	5	(3%)
	Employed	1044	(28%)	28	(23%)	592	(29%)	43	(27%)
	Self employed no employees	176	(5%)	5	(4%)	105	(5%)	9	(6%)
	Self employed with employees, employed supervisor	835	(22%)	22	(18%)	513	(26%)	31	(20%)
	Unknown	84	(2%)	4	(3%)	22	(1%)	3	(2%)
Mother	White	3236	(86%)	94	(78%)	1795	(89%)	141	(89%)
	Black (African or Caribbean)	103	(3%)	6	(5%)	36	(2%)	4	(3%)
	South East Asian	231	(5%)	13	(11%)	78	(4%)	7	(4%)
	Other / Unknown	200	(5%)	8	(7%)	99	(5%)	6	(4%)

Confidential: For Review Only

		Phase I				Phase II			
		Total		Assessment		Total		Assessment	
Fathers age at birth (Median, IQR)		34	[31,38]	34	[31,37]	35	[31,39]	35	[31,39]
Father education	None / <5 GCSE	613	(16%)	22	(18%)	268	(13%)	23	(15%)
	5 GCSE	477	(13%)	11	(9%)	223	(11%)	14	(9%)
	2+ A levels	439	(12%)	14	(12%)	232	(12%)	22	(14%)
	Degree	1547	(41%)	41	(34%)	983	(49%)	62	(39%)
	Unknown	694	(18%)	33	(27%)	302	(15%)	37	(23%)
Father occupation	Not working	166	(4%)	10	(8%)	61	(3%)	5	(3%)
	Student	40	(1%)	1	(1%)	14	(1%)	1	(1%)
	Employed	1153	(31%)	38	(31%)	634	(32%)	51	(32%)
	Self–employed – no employees	351	(9%)	11	(9%)	196	(10%)	20	(13%)
	Self–employed – with employees, employed supervisor	1729	(46%)	41	(34%)	994	(49%)	66	(42%)
	Unknown	331	(9%)	20	(17%)	109	(5%)	15	(9%)
Father ethnicity	White	3171	(84%)	96	(79%)	1780	(87%)	138	(87%)
	Black (African or Caribbean)	142	(4%)	8	(7%)	52	(3%)	6	(4%)
	South East Asian	237	(6%)	10	(8%)	78	(4%)	6	(4%)
	Other / Unknown	220	(6%)	7	(6%)	98	(5%)	8	(5%)

Confidential: For Review Only

Supplementary Figure 1: Phase 1 study flow

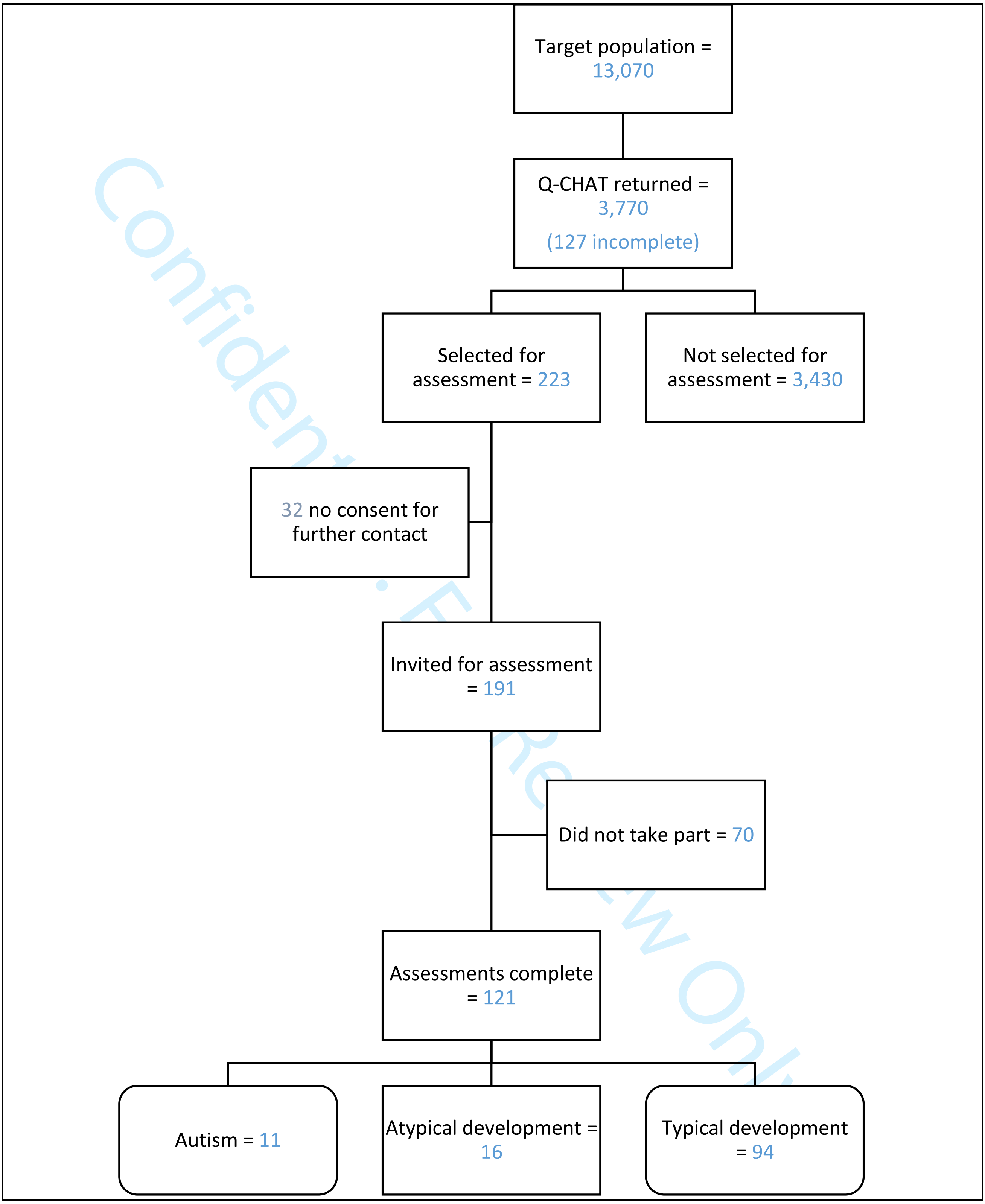

Confidential: For Review Only

Supplementary Figure 2: Distribution of Q–CHAT at first interview, with marks for screening groups

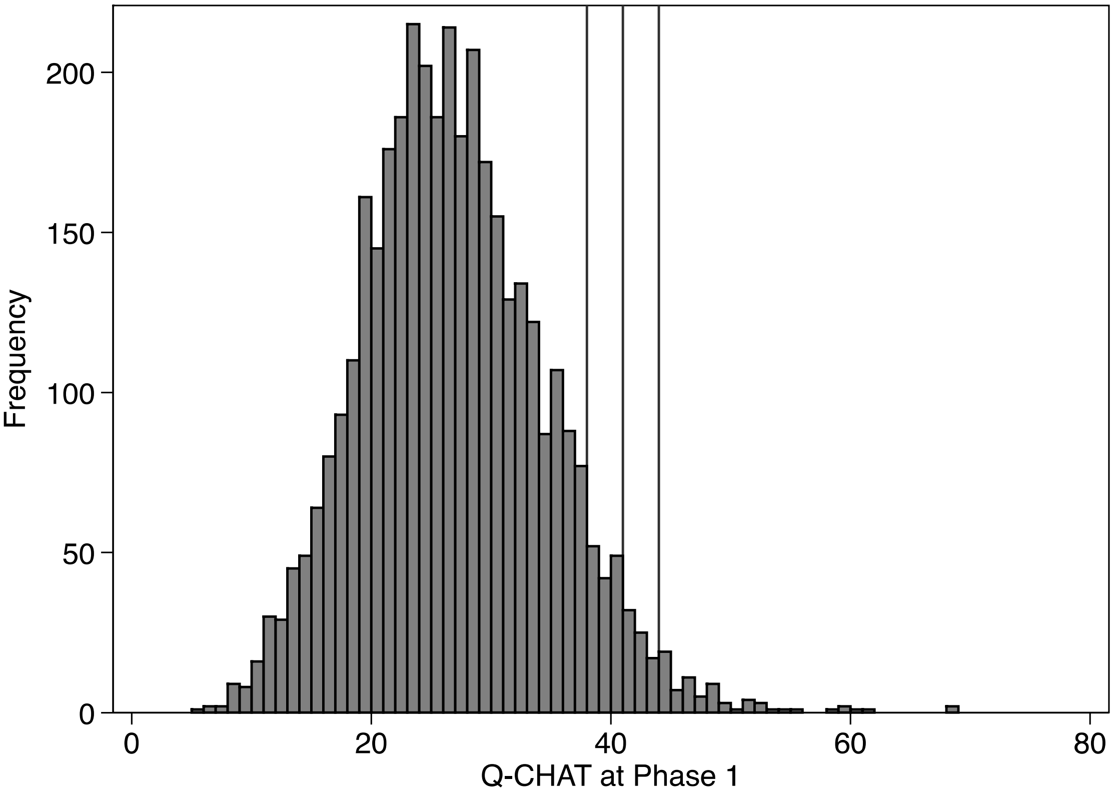

Review Only

Supplementary Figure 3: ROC curves for Q–CHAT and CAST on Phase 1 and Phase 2 outcomes for autism

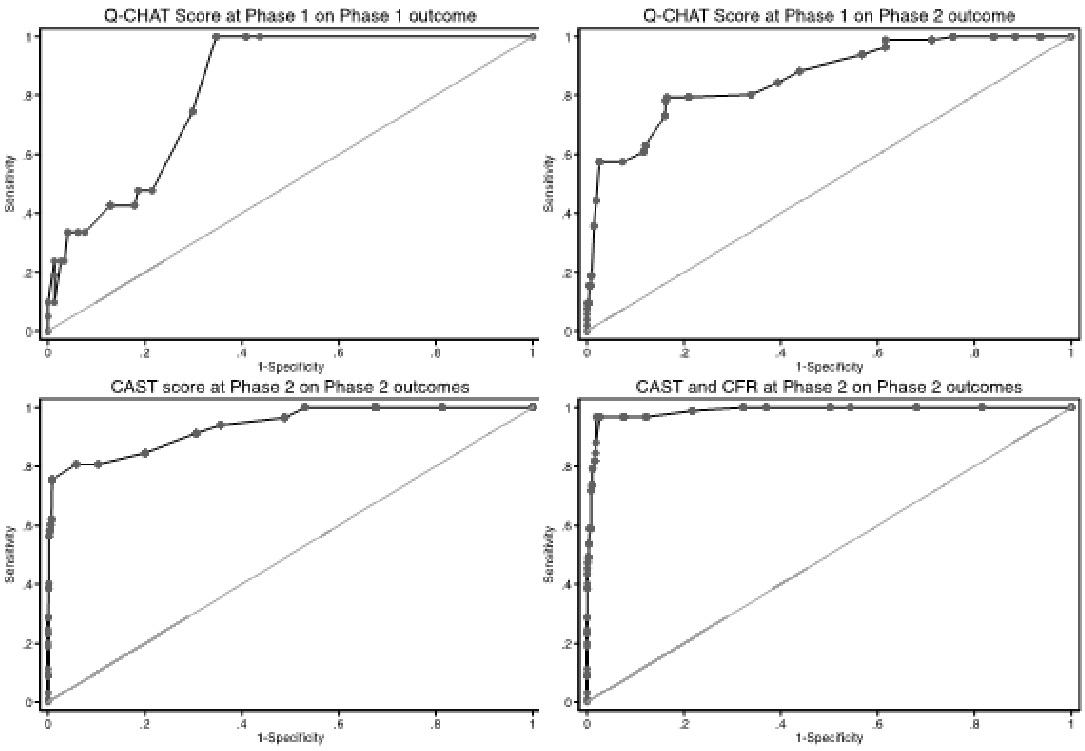

For Peer Review Only

Supplementary Figure 4: Box-plot of the distribution of Q-CHAT scores split by both Phase 1 and Phase 2 diagnosis

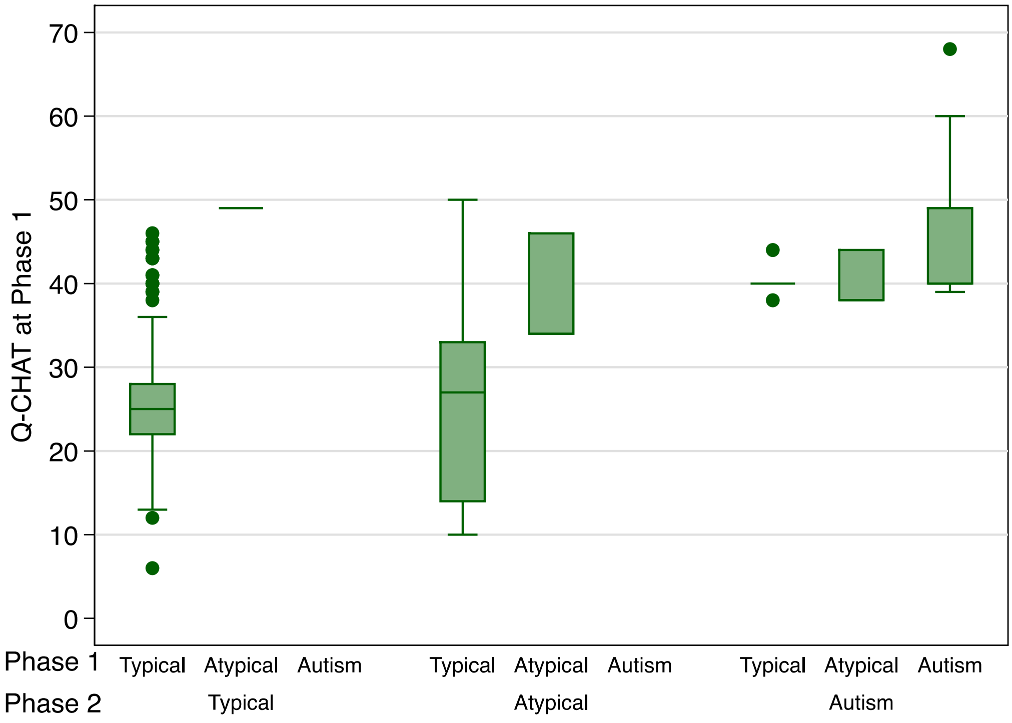

For Review Only

BMJ Paediatrics Open

The Quantitative Checklist for Autism in Toddlers (Q–CHAT). A population screening study with follow-up: the case for multiple time–point screening for autism.

Journal:	*BMJ Paediatrics Open*
Manuscript ID	bmjpo-2020-000700.R2
Article Type:	Original research
Date Submitted by the Author:	13-Apr-2021
Complete List of Authors:	Allison, Carrie; Autism Research Centre, Psychiatry Department<br>Matthews, Fiona; Newcastle University, Population Health Sciences Institute, Campus for Ageing and Vitality<br>Ruta, Liliana; National Research Council, Institute of Applied Sciences and Intelligent Systems, "Eduardo Caianiello" (ScienceApp)<br>Pasco, Greg; Institute of Psychiatry Psychology and Neuroscience, Psychology<br>Soufer, Renee; Autism Research Centre, Psychiatry Department<br>Brayne, Carol; University of Cambridge, Institute of Public Health<br>Charman, Tony; King's College London, Institute of Psychiatry, Department of Psychology<br>Baron-Cohen, Simon; Autism Research Centre, Psychiatry Department
Keywords:	Autism, Screening

SCHOLARONE™
Manuscripts

I, the Submitting Author has the right to grant and does grant on behalf of all authors of the Work (as defined in the below author licence), an exclusive licence and/or a non-exclusive licence for contributions from authors who are: i) UK Crown employees; ii) where BMJ has agreed a CC-BY licence shall apply, and/or iii) in accordance with the terms applicable for US Federal Government officers or employees acting as part of their official duties; on a worldwide, perpetual, irrevocable, royalty-free basis to BMJ Publishing Group Ltd ("BMJ") its licensees and where the relevant Journal is co-owned by BMJ to the co-owners of the Journal, to publish the Work in this journal and any other BMJ products and to exploit all rights, as set out in our licence.

The Submitting Author accepts and understands that any supply made under these terms is made by BMJ to the Submitting Author unless you are acting as an employee on behalf of your employer or a postgraduate student of an affiliated institution which is paying any applicable article publishing charge ("APC") for Open Access articles. Where the Submitting Author wishes to make the Work available on an Open Access basis (and intends to pay the relevant APC), the terms of reuse of such Open Access shall be governed by a Creative Commons licence – details of these licences and which Creative Commons licence will apply to this Work are set out in our licence referred to above.

Other than as permitted in any relevant BMJ Author's Self Archiving Policies, I confirm this Work has not been accepted for publication elsewhere, is not being considered for publication elsewhere and does not duplicate material already published. I confirm all authors consent to publication of this Work and authorise the granting of this licence.

Confidential: For Review Only

The Quantitative Checklist for Autism in Toddlers (Q–CHAT). A population screening study with follow-up: the case for multiple time–point screening for autism.

Confidential: For Review Only

Allison, C.*[1], Matthews, F.E. [2], Ruta, L.[1,3], Pasco, G.[4], Soufer, R.[1], Brayne, C.[5], Charman, T.[4], & Baron–Cohen, S. [1]

[1] Autism Research Centre, University of Cambridge, Department of Psychiatry, Douglas House, 18B Trumpington Road, Cambridge, CB2 8AH, UK

[2] Population Health Sciences Institute, Campus for Ageing and Vitality, Newcastle University, Newcastle upon Tyne, NE4 5PL, UK

[3] Institute of Applied Sciences and Intelligent Systems, "Eduardo Caianiello" (ScienceApp), National Research Council of Italy (CNR), Messina, Italy.

[4] Department of Psychology, Institute of Psychiatry, Psychology & Neuroscience, King's College London, DeCrespigny Park, Denmark Hill, London, SE5 8AF, UK

[5] Department of Public Health and Primary Care, Cambridge Institute of Public Health, University of Cambridge, CB2 0SR

* Corresponding author cla29@cam.ac.uk tel 01223 465233

Objectives: Prospective population screening study for autism in 18-30-month-old toddlers using the Quantitative Checklist for Autism in Toddlers (Q-CHAT), with follow-up at age 4 years.

Design: Observational Study.

Setting: Luton and Bedfordshire, and Cambridgeshire in the United Kingdom.

Participants: 13,070 toddlers registered on the Child Health Surveillance Database between March 2008 and April 2009, with follow–up at age 4; 3,770 (29%) were screened for autism at 18–30 months using the Q–CHAT and the Childhood Autism Screening Test (CAST) at follow up at age 4 years.

Interventions: A stratified sample across the Q-CHAT score distribution was invited for diagnostic assessment (Phase 1). The 4 year follow–up included the Childhood Autism Screening Test (CAST) and Checklist for Referral (CFR). All with CAST ≥15, Phase 1 diagnostic assessment, or, with developmental concerns on the CFR were invited for diagnostic assessment (Phase 2). Standardised diagnostic assessment at both time-points was conducted to establish the test accuracy of the Q-CHAT.

Main outcome measures: Consensus diagnostic outcome at Phase 1 and Phase 2.

Results: Phase 1: 3,770 Q–CHATs were returned (29% response), 121 undertook diagnostic assessment, of whom 11 met criteria for autism. All 11 screened positive on the Q–CHAT. The positive predictive value (PPV) at a cut–point of 39 was 17% (95%CI 8%–31%). Phase 2: 2,005/3,472 CASTs and CFRs were returned (58% response). 159 underwent diagnostic assessment, including 82 assessed in Phase 1. All children meeting criteria for autism were identified via the Q–CHAT at Phase 1 also met criteria at Phase 2. PPV was 28% (95%CI 15%–46%) after Phase 1 and Phase 2.

Conclusions: The Q–CHAT can be used at 18–30 months to identify autism and enable accelerated referral for diagnostic assessment. The low PPV suggests that for every true positive there would, however, be ~4-5 false positives. At follow–up, new cases were identified illustrating the need for continued surveillance and re–screening at multiple time–points using developmentally sensitive instruments. Not all children who later receive a diagnosis of autism are detectable during the toddler period.

Confidential: For Review Only

Confidential: For Review Only

Research in Context

What is already known on this topic

In the UK, a systematic population screening programme for autism is not recommended to facilitate early detection, because general population screening tests that have been evaluated using systematic follow-up have not proved accurate.

What this study adds

It is possible to detect autism at 18–30 months. It is not possible to detect every child at a very young age who will later be diagnosed as autistic. The Q–CHAT can be used to screen toddlers at 18–30 months.

Introduction

Autism affects approximately 1–2% of the population.[1, 2] Screening requires a standardised and systematic approach to detect children who may require a diagnostic assessment. The costs and benefits of autism population screening need to be carefully balanced. Undue anxiety in false positives and the need for sufficient support services for those diagnosed should be offset by the many benefits of accurate screening that include earlier diagnosis, more timely access to specific interventions[3] and better support for parents. In the UK, no autism specific screening instrument is recommended by the National Institute of Health and Care Excellence (NICE).[4] Autism is variable in onset and the significance over time of specific symptoms. No existing screening instrument, used at a single time–point, reaches a minimum of 80% sensitivity and specificity[5]. In contrast, the American Academy of Pediatrics[6] recommends routine screening at well–child checks using the Checklist for Autism in Toddlers (CHAT)[7, 8] and the Modified CHAT (M–CHAT),[9] despite the limited research evidence for their use,[10] and insufficient evidence to determine if certain risk factors modify the performance characteristics of the tests.[11]

Lengthy delays between first concerns and an eventual diagnosis are often experienced.[12] There has been no evidence of a reduction in the age at diagnosis over the past decade in the UK.[13] Beliefs held by health professionals about screening for autism (e.g. system capacity, interventions available) may influence practice.[14] Equally, young autistic children may not present salient atypical behaviour in a short consultation to warrant an assessment referral. Atypical behaviours, such as Restricted and Repetitive Behaviours (RRBs), extend to the neurotypical child on a continuum[15] and may reduce with time.[16] The reduction of inappropriate referrals to already over–stretched services could serve to benefit children, their families and health services. 60% of referrals to a child development centre in the UK for possible autism resulted in an autism diagnosis. It is unclear what the outcome was for the remainder of the referrals.[17]

The CHAT[7] is a short parent–report questionnaire combined with a health professional observation. The CHAT was tested prospectively in 18 month old toddlers from the general population with diagnostic follow–up of those who scored above the cut–point to allow for the estimation of both sensitivity and specificity. At 18 months the CHAT identified 92% of autistic children when assessed and diagnosed by two independent psychiatrists using Diagnostic and Statistical Manual of Mental Disorders, Third Edition, Revised (DSM–III–R)

https://mc.manuscriptcentral.com/bmjpo
Confidential: For Review Only

criteria as the gold standard at that time.[8] By the 6–year follow–up, the sensitivity was 38% indicating that many children did not present with sufficient features of autism to meet diagnostic criteria at 18 months. Nevertheless, the CHAT had very high specificity (98%) and was the first demonstration internationally that autism could be diagnosed as early as 18 months of age, when previously age–at–first diagnosis was typically not until later in childhood.[18] The CHAT was based on toddler developmental precursors of 'theory of mind',[19] such as joint attention[20-22] and pretend play,[23] so stemmed from a cognitive developmental theory about the nature of autism.[24]

The Modified Checklist for Autism in Toddlers (M–CHAT),[9] is a 23–item parent–report questionnaire, used in the United States healthcare system. It incorporates the original nine items from the CHAT and includes additional items that may be characteristic of a young autistic child. Initial results reported sensitivity of 0·97, specificity of 0·99, and positive predictive value (PPV) of 0·68 when used with a telephone follow–up. Despite limitations to the initial studies[25] (e.g. referred and unselected samples were combined, and no systematic follow–up), the M–CHAT has been examined in a large population sample at 18–months.[26] Specificity was high (0·93), but sensitivity was low (0·34) and PPV was 0·35. The authors concluded that it may not be possible to detect all autistic children at 18–months. The M–CHAT has subsequently been revised (M–CHAT, Revised with Follow–Up (M–CHAT–R/F)),[27] consisting of 20 items, including examples to provide developmental context and clarity. This was tested in a large population (N=16,071), finding PPV to be 0·48, while another study[28] reported PPV to be 0·54. Sensitivity could not be determined in these two studies because only the screen positives were followed up. An additional study examined the accuracy of the M-CHAT/F in a universal, primary care-based screening context that involved systematic follow-up up to 8 years.[29] Diagnoses were ascertained independent of the screen. The study reported sensitivity was 38.8%, and positive predictive value (PPV) was 14.6%, with almost universal screening being achieved (91%) by incorporating autism screening into routine check-ups and integrating these within the electronic health record.

The Quantitative Checklist for Autism in Toddlers (Q–CHAT)[30] was developed to dimensionalise autism in toddlerhood. It has been demonstrated that autistic traits can be measured quantitatively in the general population, using the Autism Spectrum Quotient (AQ) in adulthood,[31] the AQ–Adolescent,[32] the AQ–Child[33] and the Social–Responsiveness Scale (SRS),[34] in older children and adults. The Q–CHAT is a parent–report 25–item questionnaire

Confidential: For Review Only

that includes the CHAT and additional items. Each item is converted to a rating scale, thus quantifying autistic traits. This allows for the possibility of reporting behaviour at a reduced frequency. For example, the response options on the item concerning protodeclarative pointing, range from many times a day (least autistic response) through to never (most autistic response). Q–CHAT domains include social communication, repetitive, stereotyped and sensory behaviours. A preliminary study examined the distribution of the Q-CHAT in an unselected sample of 18-24 month old toddlers and in a sample of children already diagnosed on the autism spectrum. Results indicated that the Q–CHAT discriminated well between young autistic children and unselected toddlers in the population at 18–24 months.[30] The Q-CHAT was not evaluated as a prospective screen in this sample. The Q-CHAT has been translated and validated in other countries, including Italy and Chile.[35, 36] A short version of the Q–CHAT (Q–CHAT–10)[37] was developed and tested retrospectively on toddlers with an autism diagnosis compared to toddlers from the unselected population reported. Sensitivity and specificity were 0·91, and 0·89 respectively. However, these studies were not longitudinal, so cannot address the question of whether it is best to screen for autism at single or multiple time–points. See Petrocchi et al[38] for a recent systematic review outlining the psychometric properties of autism specific screening instruments.

The objective of this study is to report a population screening study of nearly 4,000 toddlers at 18–30 months using the Q–CHAT with diagnostic assessments on a sub-sample of responders, and subsequent follow–up at four years. The child health surveillance database was used to identify the population eligible to screen with the Q-CHAT. The study is reported in two phases, as undertaken. The aims of Phase 1 were: i) to determine test accuracy of the Q–CHAT in the toddler period and; ii) to determine an optimal screening cut-point that could be used to select toddlers for a diagnostic assessment. The aims of Phase 2 were i) to rescreen the population at a minimum age of four using the Childhood Autism Spectrum Test (CAST)[39] in order to identify autistic children who were not detected at Phase 1 (false negatives) and the Checklist for Referral (CFR) to seek information on the child's history of developmental concerns; ii) to confirm the outcome of those who were detected by the Q-CHAT at 18 – 30 months, and those who were not, by conducting diagnostic assessments; iii) to assess the stability of the diagnostic outcome from Phase 1 to Phase 2, and; iv) to assess the discriminant power of the Q–CHAT and CAST and CFR in distinguishing autism cases from non–autism cases. This two–phase design allowed us to report sensitivity, specificity

https://mc.manuscriptcentral.com/bmjpo

and PPV. Test accuracy at Phase 1 was not evaluated at the time to ensure researchers remained blind to the results when undertaking Phase 2.

Methods

Patients and Public Involvement Statement

The first and senior authors (CA and SBC) gave talks to parent support groups and clinicians about this research during all phases of the research, and received feedback through questions and discussion. The format of the Q-CHAT itself was co-designed with a parent support group.

Setting, Study Design and Procedure

Phase 1

All caregivers of infants between the ages of 18-30 months who were registered on the Child Health Surveillance Database at the three Primary Care Trusts (PCT) on the date of mailing were invited to complete the Q-CHAT between March 2008 and April 2009. Questionnaires were sent in three batches to manage capacity of the team. Screening was conducted via a postal questionnaire, distributed by the PCT. Luton questionnaires were mailed in March 2008. Bedfordshire questionnaires were mailed in May 2008. Cambridgeshire questionnaires were mailed in April 2009. Questionnaires were posted twice, two weeks apart, to maximise response. Returned questionnaires were scored by the research team and the sampling strategy as detailed below, was applied to establish the diagnostic assessment sample. Research diagnostic assessments were arranged as soon as possible after completion of the Q-CHAT. All members of the diagnostic assessment team remained blind to the child's Q-CHAT score. The Q-CHAT was completed a second time prior to any of the diagnostic assessment battery in order to later examine the test-retest reliability of the Q-CHAT in a sample enriched with high scorers, which will be reported separately. Diagnostic outcome was defined by the researchers reaching a consensus diagnosis using all diagnostic assessment data and researcher judgement.

Phase 1 Sampling Strategy

The strategy for selecting participants for follow-up diagnostic assessment was weighted towards those with higher scores. The rationale for choosing 44 as the cut-point for inviting participants for follow-up diagnostic assessment at Phase 1 was based on anticipated estimates of prevalence of autism (approximately 1%) balanced with the knowledge that over

Confidential: For Review Only

70% of the children who already had a diagnosis on the autism spectrum scored 44 and above in our initial study[30]. 44 was therefore chosen in order to maximise sensitivity whilst generating a feasible number of diagnostic assessments to be completed. In contrast, the strategy for identifying the optimal screening cut-point on the Q-CHAT was not determined until after Phase 2 diagnostic assessments were complete, which was one the of the aims of the study. At a cut-point of 44 on the Q-CHAT at Phase 1, 100% of children were selected for diagnostic assessment. To ensure that children with missing data were not missed from being included in a high scoring group (44+), missing Q-CHAT items were allocated the maximum item score (four) and the total Q-CHAT score recalculated. The rationale for incorporating missing data into the sampling strategy was to ensure that children with a high likelihood of having many autistic traits (and therefore potentially being autistic) but who had sufficient missing data to put them in a sampling band where the chance of being selected was low, were included in the follow up diagnostic assessments. Each Q-CHAT questionnaire therefore had two scores: the observed (assuming each item of missing data would score as not impaired) and the maximum (assuming each item of missing data would score as impaired). Observed and maximum Q-CHAT scores were grouped into four bands (≥44, 41-43, 38-40, ≤37). This ensured that missing data meant a child was more likely to be assessed.

The final sampling groups were determined according to the maximum score, taking into account the movement across score groups from observed score to maximum score. For example, if a child's observed score was 40 and there were two missing Q-CHAT items, eight was added to the observed score to give 48. Forty eight would therefore be that child's sampling score. Sampling group allocation and randomisation took place prior to establishing whether or not the family had consented for further contact.

When the chance of being selected was less than 100%, selection for diagnostic assessment was undertaken using a random number generator (www.random.org). 1% of children who had a maximum score ≤37 were selected for diagnostic assessment to ensure that the diagnostic assessment team were blind to whether there were likely to be concerns about the child at the time of diagnostic assessment.

Phase 2

All caregivers who consented to further contact following the Phase 1 screening (N=3,472) were invited to participate in Phase 2 at a minimum age of 48 months, between January and

Confidential: For Review Only

July 2011. Caregivers were sent the Childhood Autism Spectrum Test (CAST), a 37-item tool to identify children at risk for autism in children aged 4–11 years, in a non-clinical setting[39, 40], Parents were also sent a brief checklist enquiring about whether the child had ever been referred or diagnosed with any developmental and/or medical condition (Checklist for Referral, CFR).

Phase 2 Sampling

All children who scored ≥15 on the CAST (the recommended screening cut-point for epidemiological research)[33] were invited for a Phase 2 diagnostic assessment, along with all children whose caregiver indicated on the CFR that they had been referred for any of the following reasons: language delay/disorder, Hyperactivity/Attention Deficit Disorder (ADHD, ADD), dyspraxia, autism spectrum condition (autism), genetic/chromosomal abnormality, epilepsy, Tourette syndrome, and tuberous sclerosis. There was also a space on the CFR for the caregiver to provide more information about the reason for the referral and whether any diagnosis had been made. If the caregiver indicated that their child had been referred for any other reason not specified on the CFR, the research team decided on a case–by–case basis whether or not to invite the family for the diagnostic assessment. All children who participated in Phase 1 diagnostic assessments were invited for a Phase 2 diagnostic assessment in order to assess the stability of the diagnostic outcome from Phase 1 to Phase 2.

Diagnostic Outcome

In both Phase 1 and 2, diagnostic outcome was defined by the researchers reaching a consensus diagnosis using all diagnostic assessment data and researcher judgement as possible autism or autism spectrum (if they met the International Statistical Classification of Diseases 10th Edition (ICD-10) criteria[41] for a pervasive developmental disorders diagnosis), atypical (if the child showed developmental concerns that were not related to autism) or typical (if there were no developmental concerns about the child). We used the term 'possible' to reflect the reluctance of some clinicians to commit to an autism diagnostic label at such an early age. It was not possible for all of the diagnostic assessment team to remain blind to the diagnostic outcome from the Phase 1 diagnostic assessments at Phase 2. However, the diagnostic assessment team re-examined blind to the Q-CHAT score throughout Phase 2 diagnostic assessments.

Confidential: For Review Only

Ethical approval was granted by the Cambridge Local Research Ethics Committee (05/Q0108/53). The University of Cambridge was the sponsor of this research. Informed consent was obtained from all caregivers included in the study.

Measures

Q-CHAT

The Q-CHAT has a forced choice design, with five possible responses for each item (with the exception of item 4, see below). For each item, response representing the most 'autistic' symptomatology scores four points and the least 'autistic' response scores zero. On approximately half the items (items 3, 7, 8, 11, 12, 13, 16, 18, 20, 22, 23, 24, 25) the 'autistic' response is the positive one. On the other half, (items 1, 2, 4, 5, 6, 9, 10, 14, 15, 17, 19, 21) the 'autistic' response is the negative one. Item 4 is concerned with speech and includes a sixth option of 'my child does not speak', also scoring four points. Total Q-CHAT score is obtained by summing the score on each item, giving a maximum of 100. Each of the 25 questions is accompanied by a colour illustration to attempt to increase both response and comprehensibility of the items.

Demographic Data

Data collected about the child included age, sex, birth order, ethnicity and multiple birth status. Data collected about the caregivers included Socio-Economic Status (SES) and parental educational attainment. SES was derived at person level rather than household level, using the five class system of the National Statistics-Socio Economic Classification (NS-SEC)[42]. Parental educational attainment was assessed by the highest level of qualification of each parent.

Diagnostic Assessment

The ADOS-G[43] was completed. The ADOS-G is a semi-structured, standardized assessment of social interaction, communication, play, and imagination. It is a reliable and valid measure for the diagnosis of autism relative to those with non-autistic conditions. Module 1 was chosen at Phase 1 since it was not expected that many children would have expressive language abilities beyond basic phrase speech. Module 2 or 3 was chosen at Phase 2. ADOS-G assessments were videotaped, but item scoring was conducted independently by two members of the diagnostic assessment team and consensus codes were agreed as soon as possible after the diagnostic assessment. The diagnostic assessment team comprised of at

Confidential: For Review Only

least one very experienced research psychologist alongside a research assistant who also had been trained to reliability standards.

The Autism Diagnostic Interview-Revised (ADI-R)[44] was completed with the caregiver. The ADI-R is a diagnostic semi-structured interview that generates an algorithm score based on behaviours in three domains: social communication, social interaction and repetitive and stereotyped behaviours and interests. The interview also enquires about age of onset of the symptoms. Due to the practicalities in arranging the diagnostic assessments, it was not possible to counterbalance completion of the ADOS-G and ADI-R. However, the ADI-R and ADOS-G was never carried out by the same researcher to reduce the possibility of experimenter expectation bias. All ADI-R assessments were audio-taped.

The Mullen Scales of Early Learning (MSEL)[45] is a standardised measure of cognitive, expressive and receptive language, motor and perceptual abilities. The MSEL was usually carried out while the other researcher was completing the ADI-R with the caregiver. The gross motor scale was not completed as this does not contribute to the overall Early Learning Composite (ELC). The Vineland Adaptive Behavior Scale (VABS)[46] is a standardised measure of personal and social skills. The interview version was completed with the parent. There are four domains that measure communication, daily living skills, socialisation and motor skills, as well as an optional maladaptive domain (not completed here). The VABS was usually completed at the end of the testing session.

Confidential: For Review Only

Table 1: Phase 1 Follow-up Diagnostic Assessment Sampling Strategy, with study numbers

Sampling Group	Observed Q-CHAT (missing items = 0)	Maximum Q-CHAT (missing items = 4)	% sampled	Total	Assessment numbers		
					Selected	Invited	Agreed
1	≥38	≥44	100%	86	86	74	40
2	≤37	≥44	50%	21	10	9	6
3	41–43	41–43	50%	61	31	28	16
4	≤40	41–43	50%	21	10	8	5
5	38–40	38–40	25%	129	31	28	20
6	≤37	38–40	25%	25	5	5	2
7	≤37	≤37	1%	3300	40	39	32
**Total**				**3643**	**213**	**191**	**121**

Confidential: For Review Only

Statistical methods

Due to the stratified sample all analyses were adjusted for the probability of being selected into the diagnostic assessment subsample at each Phase via inverse probability weighting. This takes account of the differences in sampling proportions and the selective participation in the diagnostic assessment across the sampling groups. A sensitivity analysis also included within the weights are the health trust non–response rates at each stage. A receiver operator characteristic (ROC) area under the curve (AUC) analysis assessed the discriminant power of the Q–CHAT and CAST and CFR in distinguishing autism cases from non–autism cases (defined as typical and atypical) and from autism and all other atypical development from typically developing children, across all score values. Confidence intervals were obtained from robust standard errors. All analysis was undertaken in Stata 14.[47] A series of sensitivity analyses to decisions on the screening instrument were undertaken (see Table 2). At both phases, these included examining the cut-point at 31 and 38, as well as the cut-point of 39 including those parents that reported concerns, and 39 adjusted for initial non-response at screening.

Role of the funding source

The funding source had no role in the data collection, data analysis, data interpretation, or writing of the report. The corresponding author had full access to all the data in the study and had final responsibility for the decision to submit for publication.

Results

Phase 1

3,770 Q–CHAT questionnaires were returned: 436 (14% response) from Luton, 1,256 (25% response) from Bedfordshire and 2,078 (42% response) from Cambridgeshire. 223 were selected for diagnostic assessment, 32 did not give consent to be contacted, therefore 191 were invited and 121 competed the diagnostic assessments. See Table 1 for Phase 1 sampling strategy with study numbers, Supplementary Figure 1 for the flow chart from phase 1 and Supplementary Table 1 for characteristics of children and their families throughout the study, supplementary Figure 2 shows the distribution of Q–CHAT with sampling bands indicated.

Following diagnostic assessment, 11 children were defined as possible autism, 16 as atypical (7 language delay, 5 developmental delay, 4 other atypical), and 94 as typical. At a cut–point of ≥39, the positive predictive value (PPV) for autism was 17% (95%CI 8%–31%) (Table 2).

Confidential: For Review Only

Autism	TP	FN	FP	TN	Sensitivity	Specificity	PPV	NPV	AUC
**Main analysis – phase 1**									
Q–CHAT 39+	36	0	179	3428	1.00 (0.72–1.0)*	0.95 (0.92–0.97)	0.17 (0.08–0.31)	1.00 (0.93–1.0)*	0.98 (0.96–1.00)
**Main analysis – phase 2**									
**Q–CHAT 39+**	**27**	**34**	**69**	**3513**	**0.44 (0.26–0.64)**	**0.98 (0.97–0.99)**	**0.28 (0.15–0.46)**	**0.99 (0.98–0.99)**	**0.86 (0.78–0.94)**
CAST ≥15+ / CFR positive	50	7	98	3488	0.88 (0.63–0.97)	0.97 (0.95–0.98)	0.34 (0.23–0.46)	1.00 (0.99–1.00)	0.99 (0.98–1.00)
**Sensitivity analysis – phase 1**									
Q–CHAT 31+	36	0	1104	2504	1.00 (0.72–1.0)*	0.69 (0.53–0.82)	0.03 (0.01–0.07)	1.00 (0.88–1.0)*	0.98 (0.96–1.00)
Q–CHAT 38+	36	0	238	3369	1.00 (0.71–1.0)*	0.93 (0.90–0.96)	0.13 (0.06–0.25)	1.00 (0.92–1.0)*	0.98 (0.96–1.00)
Q–CHAT 39+ with parental concern	30	6	73	3534	0.84 (0.43–0.97)	0.98 (0.97–0.99)	0.29 (0.13–0.52)	1.00 (0.99–1.00)	1.00 (0.99–1.00)
Q–CHAT 39+ adjust initial non–response	49	0	201	3393	1.00 (0.72–1.0*)	0.94 (0.91–0.97)	0.20 (0.09–0.37)	1.00 (0.93–1.0)*	0.98 (0.96–1.00)
**Sensitivity analysis – phase 2**									
Q–CHAT 31+	48	13	589	2993	0.79 (0.64–0.89)	0.84 (0.65–0.93)	0.08 (0.03–0.19)	1.00 (0.99–1.00)	0.86 (0.78–0.94)
Q–CHAT 38+	35	26	92	3489	0.57 (0.39–0.74)	0.97 (0.96–0.99)	0.28 (0.16–0.43)	0.99 (0.99–1.00)	0.86 (0.78–0.94)
Q–CHAT 39+ with parental concern	24	37	29	3553	0.40 (0.23–0.60)	0.99 (0.98–1.00)	0.46 (0.24–0.69)	0.99 (0.98–0.99)	0.90 (0.84–0.96)
Q–CHAT 39+ adjust initial non–response	38	35	73	3497	0.51 (0.30–0.72)	0.98 (0.96–0.99)	0.34 (0.17–0.55)	0.99 (0.98–0.99)	0.89 (0.81–0.96)

https://mc.manuscriptcentral.com/bmjpo

Confidential: For Review Only

CAST ≥15	32	25	8	3578	0.56 (0.37–0.74)	1.00 (0.99–1.00)	0.81 (0.59–0.92)	0.99 (0.99–1.00)	0.94 (0.88–0.99)
Q–CHAT 39+ include parent reported cases	30	34	67	3512	0.47 (0.30–0.65)	0.98 (0.97–0.99)	0.31 (0.52–0.82)	0.99 (0.98–0.99)	0.86 (0.79–0.94)

Table 2: Autism diagnosis results Phase 1 and Phase 2

* One–sided confidence interval due to perfect predictor (not weighted)
TP, true-positive cases. FN, false-negative cases; FP, false-positive cases; TN, true-negative cases; PPV, positive predictive value; NPV, negative predictive value; AUC, area under the receiver operating characteristic curve

Confidential: For Review Only

Phase 2

3,472 consenting caregivers from Phase 1 were sent the Phase 2 study materials. 2,005 returned both the CFR and CAST (58% response), a further 6 returned just the CAST and 5 returned just the CFR. 42 children who had a diagnostic assessment in Phase 1 did not return the CAST or CFR.

172 caregivers reported developmental concerns on the CFR and 43 children scored 15 and above on the CAST (not in Phase 1 diagnostic assessment sample) and were selected for diagnostic assessment. 81 children who had been assessed in Phase 1 completed the diagnostic assessment (1 child did not complete all the diagnostic assessments and is therefore not included in the analysis). In total, 158 children completed the diagnostic assessment at Phase 2, 23 with CFR concerns and CAST score ≥ 15, 63 with CFR concerns and CAST scores ≤ 15, 6 with CAST scores of ≥ 15 with no CFR concerns, 56 who scored ≤ 15 on the CAST and who did not report any developmental concerns on the CFR but who were assessed in Phase 1, and 10 children who were assessed in Phase 1 but who did not return the CFR or CAST (Figure 1 summarises the overall study design, with Figure 2 showing the characteristics and comparison of the Q-CHAT at Phases 1 and 2, and the CAST at Phase 2 (not weighted). 11 children were considered as possibly autism in Phase 1 (prevalence of 0·98%, 95%CI 0·45%–2·16%).

81 children were assessed at both Phase 1 and 2. Of the 9 children whose diagnostic outcome at Phase 1 was autism who had a diagnostic assessment in Phase 2, all were still classified as autism. A further 6 children were now classified as autism (4 who were typical at Phase 1, and 2 who were atypical). 77 children were assessed for the first time at Phase 2, having completed a Q-CHAT at Phase 1. Some of these children were invited for diagnostic assessment due to indicating on the CFR that they had received a clinical diagnosis of autism, and others because of their CAST score (15 or above). Of these 29 were classified as autism, 24 as atypical development and 24 as typically developing, giving a prevalence of autism of 1·57% (95%CI 0·90%–2·74%). Figure 3 shows participant study flow through screening and diagnostic assessments from Phase 1 to Phase 2. Clinical characterisation of the sample by screening status will be reported in a separate paper.

https://mc.manuscriptcentral.com/bmjpo
Confidential: For Review Only

Insert Figure 1 here

Using the CAST alone as the screen at Phase 2, 1,994 children had complete data. At a cut–point of ≥15, sensitivity was 56% (95%CI 37%–74%) specificity was 100% (95%CI 99%–100%), and PPV was 81% (95%CI 59%–92%) (Table 2). The area under the ROC curve was 0·94 (95%CI 0·88–0·99) (Supplementary Figure 3). Adding in the CFR selection criteria improved sensitivity to 88% (95%CI 63%–97%) but decreased specificity slightly to 97% (95%CI 95%–98%), with a PPV of 34% (95%CI 23%–46%) and an area under the ROC curve of 0·99 (95%CI 0·98–1·00). Supplementary Figure 4 shows a box plot of the distribution of Q-CHAT scores split by both Phase 1 and Phase 2 diagnosis.

At a cut–point of ≥39, the sensitivity of the Q–CHAT to predict Phase 2 outcome (autism vs atypical and/or typical) was 44% (95%CI 26%–64%), specificity was 98% (95%CI 97%–99%), and PPV was 28% (95%CI 15%–46%) (Table 2). The area under the ROC curve was 0·86 (95%CI 0·78–0·94). The cut-point of 39 was determined by the data obtained at Phase 1 and Phase 2. We selected participants across the threshold of 44 following the Q-CHAT at Phase 1 in varying proportions in order to accurately determine where the cut-point should lie. Examination of the Q-CHAT data against diagnostic outcome, suggested that the cut-point of 39 is the point that maximises sensitivity and specificity at 18 months.

Sensitivity analyses: Optimising the Q–CHAT cut–point for Phase 2 outcome found two additional cut–points, Q–CHAT 31+ maximized sensitivity and specificity at the cost of false positives and using Q–CHAT 38+ marginally improved sensitivity with little additional benefit. Other sensitivity analyses at Phase 1 and Phase 2 are shown in Table 2. See Figure 2 for characteristics and comparison of the Q-CHAT at Phases 1 and 2, and the CAST at Phase 2 (not weighted), according to diagnostic outcome.

Insert Figures 2 and 3 here

Discussion

The first two aims of the study were to determine the optimal screening cut-point on the Q-CHAT, and to determine the test accuracy. At Phase 1 outcome, all children who were classified as possible autism failed the Q–CHAT at a cut–point of 39, demonstrating that early detection and diagnosis of autism is possible. The PPV of the Q–CHAT for autism was

https://mc.manuscriptcentral.com/bmjpo
Confidential: For Review Only

17%. Misinterpretation of behaviours that are not well–established as well as developmental immaturity may contribute to low PPV. An aim of Phase 2 was to rescreen the population at a minimum age of four using the Childhood Autism Spectrum Test (CAST) in order to identify autistic children who were not detected at Phase 1 (false negatives) and the Checklist for Referral (CFR) to seek information on the child's history of developmental concerns and diagnoses. The results indicated that the Q-CHAT did not identify all children who were later diagnosed as autistic by age four. We therefore were able to confirm the outcome of those who were detected by the Q-CHAT at 18 – 30 months, and those who were not. A further aim at Phase 2 was to confirm the discriminant power of the Q–CHAT and CAST and CFR in distinguishing autism cases from non–autism cases. At a cut–point of ≥39, the sensitivity of the Q–CHAT to predict autism was 44% (95%CI 26%–64%), specificity was 98% (95%CI 97%–99%), and PPV was 28% (95%CI 15%–46%). This demonstrates that the Q–CHAT alone cannot be used to detect all autistic children. This might reflect the Q–CHAT properties, but equally it likely reflects a subgroup of autistic children who do not show symptoms of sufficient 'severity' until later in childhood, and/or that symptoms of autism change with age, so require developmentally sensitive instruments at different ages. DSM-5's caveat that symptoms might not fully manifest until social demands exceed capacities may apply here. The CAST picked up many of the cases of autism that were missed by the Q–CHAT, demonstrating the need for repeat screening rather than relying on a single instrument at one time point. However, the sensitivity of the CAST at a cut point of 15 was only moderate (56%), compared to our earlier study showing sensitivity of 100%.[39] All children who participated in Phase 2 diagnostic assessments who were identified as 'possible autism' at Phase 1 were still classified as autistic, suggested the stability of the diagnosis from Phase 1 to Phase 2.

A strength of this study was the prospective, population–based design that allowed us to assess the Q–CHAT accurately at two time–points. Our approach to missing data and subsequent sampling strategy was conservative, and ensured maximum capture of potential cases in the diagnostic assessment phase, maximising sensitivity. However, studies such as the current one are challenged by modest participation rates, selection bias and attrition over time. Despite adopting strategies to attempt to increase response, overall it was low, but not unusual for an unsolicited postal survey. Whether or not those who responded initially to the Q–CHAT (which determined the population for the rest of the study) were biased towards those with concerns (either justified or the 'worried well') or in the opposite direction

https://mc.manuscriptcentral.com/bmjpo

remains largely unknown. A second limitation is that in Phase 1, only 1% of children who scored ≤37 were sampled. This proportion was chosen for pragmatic reasons in relation to feasibility (i.e. to keep the number of lengthy diagnostic assessments in children with no developmental concerns to a minimum, and to reduce expectation bias in the research team). None of the sampled children with scores ≤37 met our diagnostic assessment case definition. It is important to underline that these results are based purely on outcome at Phase 1 and do not reflect the overall test accuracy statistics from Phase 2.

Few studies attempt population screening, partly because they are very time consuming and expensive. Younger toddlers have mild or transient developmental delays that resolve, resulting in low PPV, as was found in another study.[48] Introducing a follow–up interview (such as used with the M–CHAT[25, 27, 28]) for those who screen positive may reduce the number of unnecessary referrals for a diagnostic assessment, especially with children older than 20 months.[49] Nevertheless, a recent meta–analysis of M–CHAT studies found a lack of evidence on its performance in low–risk children or at age 18 months.[50] A universal, primary care-based screening study was conducted using the Modified Checklist for Autism in Toddlers with Follow-Up (M-CHAT/F) in the United States. Overall, the sensitivity was 38.8%, and PPV was 14.6%. Sensitivity was higher in older toddlers and with repeated screenings, whereas PPV was lower in girls[29]. This study was the first using the M-CHAT to follow up children screened in the population, thus allowing for an accurate estimate of the sensitivity of the instrument. Similar results were found with another population-based sample (the ABC cohort), whereby the majority diagnosed as autistic at the 6 year follow up scored below cut-off on the M-CHAT at 18 months[26]. Since the publication of the original CHAT[7, 8, 18] no study in the UK to date has achieved screening data on such a large population sample of toddlers and accurately determined sensitivity through follow up.

Total Q–CHAT score was used to determine which sampling band each child fell into. A sum (total) score based on equal weighting of all items may not be the most appropriate method by which to quantify an individual's autistic traits. Other work by our group has shown that there are at least two items on the Q–CHAT whereby the prevalence of endorsement of the trait in the autistic direction is lower for toddlers with a diagnosis of autism, compared to population controls (items 8 and 18).[37] One study[51] found specific item clusters which gave high sensitivity and specificity values at 18 months (100% and 93.9 respectively). Item response analysis will be conducted to determine whether every item equally contributes

Confidential: For Review Only

useful information about the underlying latent dimensions. Defining a threshold on each factor that can be measured quantitatively (continuous approach) may be a more appropriate scoring method. Furthermore, exploration of different age–specific scoring algorithms should be implemented to determine which variables carry greatest relative importance to optimise screening results.[3, 49]

Overall, this study demonstrates that (1) that the Q–CHAT detected every child who received an autism diagnosis at Phase 1 outcome and therefore it is possible to detect autism at 18–30 months; and (2) it is not possible to detect every child at a very young age who will later be diagnosed as autistic. The Q–CHAT can be used to screen toddlers at 18–30 months for autism to enable accelerated referral into the diagnostic pathway. However, the PPV is low, thus potentially generating a high number of children who may not have autism, but who may still require a developmental assessment to determine whether they warrant a referral for intervention and support. Outcome data from Phase 2 revealed many autistic children that the Q–CHAT did not identify. The autism spectrum is broad and not every child will be detectable as a young toddler. Therefore, when looking at the Q–CHAT longitudinally, it is evident that screening at a single time–point results in less than optimal test properties. In a future study, we will evaluate whether the missed cases reflect a subgroup of autistic children whose symptoms are not severe enough to warrant a diagnosis during toddlerhood, and that the symptoms of autism change with age. We therefore recommend continued surveillance and re–screening at multiple time–points using developmentally sensitive instruments in order to identify a higher proportion of cases for maximal public health impact, which is in line with the recommendation by the American Academy of Pediatrics[52]. This study demonstrates that no single instrument will detect all cases of autism at a single time point. Thus, continued monitoring throughout the life course is necessary in order to identify when autistic traits begin to impair an individual's life. High quality evidence from random controlled trials is needed to determine whether early detection and consequent early implementation of specific interventions is able to change outcomes in autistic toddlers.

Word Count: 6010

Figure 1: Study Design

Figure 2: Characteristics and comparison of the Q-CHAT at Phases 1 and 2, and the CAST at Phase 2 (not weighted)

https://mc.manuscriptcentral.com/bmjpo
Confidential: For Review Only

Figure 3: Participant study flow through screening and diagnostic assessments from Phase 1 to Phase 2

Funding: The study was funded by the National Lottery Community Fund, the Autism Research Trust, and was conducted in association with the Gillings Family Charitable Trust and the National Institute for Health Research (NIHR) Collaboration for Leadership in Applied Health Research and Care (CLAHRC) East of England at Cambridgeshire and Peterborough NHS Foundation Trust, and by the NIHR Cambridge Biomedical Research Centre. The views expressed are those of the author(s) and not necessarily those of the NHS, the NIHR or the Department of Health. SBC and TC were supported by the Medical Research Council UK and the Innovative Medicines Initiative (IMI) 2 Joint Undertaking (JU) under grant agreement no. 777394. The JU receives support from the European Union's Horizon 2020 research and innovation programme and EFPIA and Autism Speaks, Autistica, and SFARI. FEM was supported by MRC U105292687. SBC and CA also received funding from the Wellcome Trust 214322\Z\18\Z, the Templeton World Charitable Foundation and SFARI. For the purpose of Open Access, SBC has applied a CC BY public copyright licence to any Author Accepted Manuscript version arising from this submission.

Acknowledgements: We are grateful to the families who participated in all phases of the study. We are also grateful to staff at Luton, Bedfordshire and Cambridgeshire CCGs for their cooperation in defining the target population and mailing the questionnaires. We are grateful to Gina Gomez, Kristelle Hudry, Sally Clifford, Georgina Woods, Emma Robson, Philippa Lewington, Susan Sadek, Kimberly Armstrong, Martine Roelfsema and Kim Davies for help with data collection.

Author Contribution: CA, FM, CB, TC and SB–C contributed to the study design. CA, LR, GP and RS collected the data. CA and FEM did the data analysis and FEM is guarantor of the analysis.

All authors contributed to data interpretation and writing of the report.

Competing Interests: Dr. Charman has served as a consultant to F. Hoffmann–La Roche Ltd and Servier. He has received royalties from Sage Publications and Guilford Publications. All other authors report no biomedical financial interests or potential conflicts of interest.

Transparency declaration: The lead author and guarantor affirms that the manuscript is an honest, accurate, and transparent account of the study being reported; that no important aspects of the study have been omitted; and that any discrepancies from the study as planned and registered have been explained.

Confidential: For Review Only

Dissemination to participants and related patient and public communities: The authors plan to send a summary of the results to participating NHS Trusts. A lay summary of the results will be available on the Autism Research Centre's (ARC) website. Results will be presented at international conferences.

Patient and public involvement: The first and senior authors (CA and SBC) gave talks to parent support groups and clinicians about this research during all phases of the research, and received feedback through questions and discussion. The format of the Q-CHAT itself was co-designed with a parent support group. Patients or the public were not involved in the research design but will be invited to comment on the policy implications of this research via the Autism Advisory Board of the ARC.

Data sharing: At the time of data collection, consent was not obtained from participants for the purposes of data sharing. Therefore anonymised data are not available.

Confidential: For Review Only

References

1. Baron-Cohen S, Scott FJ, Allison C, et al. Prevalence of autism-spectrum conditions: UK school-based population study. Br J Psychiatry. 2009; **194**(6): 500-9.
2. Maenner MJ, Shaw KA, Baio J, et al. Prevalence of Autism Spectrum Disorder Among Children Aged 8 Years - Autism and Developmental Disabilities Monitoring Network, 11 Sites, United States, 2016. Morbidity and mortality weekly report Surveillance summaries (Washington, DC : 2002). 2020; **69**(4): 1-12.
3. Zwaigenbaum L, Bauman ML, Fein D, et al. Early Screening of Autism Spectrum Disorder: Recommendations for Practice and Research. Pediatrics. 2015; **136 Suppl 1**: S41-59.
4. National Institute for Health and Clinical Excellence. Autism: recognition, referral and diagnosis of children and young people on the autism spectrum. (Clinical guideline 128). https://www.nice.org.uk/guidance/cg128; 2011.
5. Glascoe FP. Screening for developmental and behavioral problems. Ment Retard Dev Disabil Res Rev. 2005; **11**(3): 173-9.
6. Johnson CP, Myers SM. Identification and Evaluation of Children With Autism Spectrum Disorders. Pediatrics. 2007; **120**(5): 1183-215.
7. Baron-Cohen S, Allen J, Gillberg C. Can autism be detected at 18 months? The needle, the haystack, and the CHAT. Br J Psychiatry. 1992; **161**: 839-43.
8. Baron-Cohen S, Cox A, Baird G, et al. Psychological markers in the detection of autism in infancy in a large population. Br J Psychiatry. 1996; **168**(2): 158-63.
9. Robins DL, Fein D, Barton ML, Green JA. The Modified Checklist for Autism in Toddlers: an initial study investigating the early detection of autism and pervasive developmental disorders. J Autism Dev Disord. 2001; **31**(2): 131-44.
10. Al-Qabandi M, Gorter JW, Rosenbaum P. Early autism detection: are we ready for routine screening? Pediatrics. 2011; **128**(1): e211-7.
11. Siu AL, and the U. S. Preventive Services Task Force. Screening for autism spectrum disorder in young children: Us preventive services task force recommendation statement. JAMA. 2016; **315**(7): 691-6.
12. Crane L, Chester JW, Goddard L, Henry LA, Hill E. Experiences of autism diagnosis: A survey of over 1000 parents in the United Kingdom. Autism. 2016; **20**(2): 153-62.
13. Brett D, Warnell F, McConachie H, Parr JR. Factors Affecting Age at ASD Diagnosis in UK: No Evidence that Diagnosis Age has Decreased Between 2004 and 2014. J Autism Dev Disord. 2016; **46**(6): 1974-84.
14. Zwaigenbaum L, Bauman ML, Choueiri R, et al. Early Intervention for Children With Autism Spectrum Disorder Under 3 Years of Age: Recommendations for Practice and Research. Pediatrics. 2015; **136 Suppl 1**: S60-81.
15. Leekam S, Tandos J, McConachie H, et al. Repetitive behaviours in typically developing 2-year-olds. J Child Psychol Psychiatry. 2007; **48**(11): 1131-8.
16. Leekam SR, Prior MR, Uljarevic M. Restricted and repetitive behaviors in autism spectrum disorders: a review of research in the last decade. Psychol Bull. 2011; **137**(4): 562-93.
17. Williams AN, Mold B, Kilbey L, Naganna P. Forty years of referrals and outcomes to a UK Child Development Centre (CDC): Has demand plateaued? Child Care Health Dev. 2018.
18. Baird G, Charman T, Baron-Cohen S, et al. A screening instrument for autism at 18 months of age: a 6-year follow-up study. J Am Acad Child Adolesc Psychiatry. 2000; **39**(6): 694-702.
19. Baron-Cohen S. Autism: A specific cognitive disorder of "mind-blindness.". International Review of Psychiatry. 1990; **2**(1): 81-90.

Confidential: For Review Only

20. Baron-Cohen S. Perceptual role taking and protodeclarative pointing in autism. British Journal of Developmental Psychology. 1989; **7**(2): 113-27.
21. Baron-Cohen S. Joint-attention deficits in autism: Towards a cognitive analysis. Development and Psychopathology. 1989; **1**(3): 185-9.
22. Goodhart F, Baron-Cohen S. How many ways can the point be made? Evidence from children with and without autism. First Language. 1993; **13**(38, Pt 2): 225-33.
23. Baron-Cohen S. Autism and symbolic play. British Journal of Developmental Psychology. 1987; **5**(2): 139-48.
24. Charman T, Baron-Cohen S, Swettenham J, Baird G, Cox A, Drew A. Testing joint attention, imitation, and play as infancy precursors to language and theory of mind. Cognitive Development. 2000; **15**(4): 481-98.
25. Kleinman JM, Robins DL, Ventola PE, et al. The modified checklist for autism in toddlers: a follow-up study investigating the early detection of autism spectrum disorders. J Autism Dev Disord. 2008; **38**(5): 827-39.
26. Stenberg N, Bresnahan M, Gunnes N, et al. Identifying children with autism spectrum disorder at 18 months in a general population sample. Paediatr Perinat Epidemiol. 2014; **28**(3): 255-62.
27. Robins DL, Casagrande K, Barton M, Chen CM, Dumont-Mathieu T, Fein D. Validation of the modified checklist for Autism in toddlers, revised with follow-up (M-CHAT-R/F). Pediatrics. 2014; **133**(1): 37-45.
28. Chlebowski C, Robins DL, Barton ML, Fein D. Large-scale use of the modified checklist for autism in low-risk toddlers. Pediatrics. 2013; **131**(4): e1121-7.
29. Guthrie W, Wallis K, Bennett A, et al. Accuracy of Autism Screening in a Large Pediatric Network. Pediatrics. 2019; **144**(4).
30. Allison C, Baron-Cohen S, Wheelwright S, et al. The Q-CHAT (Quantitative CHecklist for Autism in Toddlers): a normally distributed quantitative measure of autistic traits at 18-24 months of age: preliminary report. J Autism Dev Disord. 2008; **38**(8): 1414-25.
31. Baron-Cohen S, Wheelwright S, Skinner R, Martin J, Clubley E. The autism-spectrum quotient (AQ): evidence from Asperger syndrome/high-functioning autism, males and females, scientists and mathematicians. J Autism Dev Disord. 2001; **31**(1): 5-17.
32. Baron-Cohen S, Hoekstra RA, Knickmeyer R, Wheelwright S. The Autism-Spectrum Quotient (AQ)--adolescent version. J Autism Dev Disord. 2006; **36**(3): 343-50.
33. Auyeung B, Baron-Cohen S, Wheelwright S, Allison C. The Autism Spectrum Quotient: Children's Version (AQ-Child). J Autism Dev Disord. 2008; **38**(7): 1230-40.
34. Constantino JN, Davis SA, Todd RD, et al. Validation of a brief quantitative measure of autistic traits: comparison of the social responsiveness scale with the autism diagnostic interview-revised. J Autism Dev Disord. 2003; **33**(4): 427-33.
35. Gatica-Bahamonde G, Alarcon P, Fadol AM, Allison C, Baron-Cohen S, Roman-Urrestarazu A. The Quantitative Checklist for Autism in Toddlers (Q-CHAT): A preliminary psychometric study in Chile. . https://doiorg/1031234/osfio/hmjkx. 2021.
36. Tartarisco G, Cicceri G, Di Pietro D, et al. Use of Machine Learning to Investigate the Quantitative Checklist for Autism in Toddlers (Q-CHAT) towards Early Autism Screening. Diagnostics (Basel). 2021; **11**(3).
37. Allison C, Auyeung B, Baron-Cohen S. Toward brief "Red Flags" for autism screening: The Short Autism Spectrum Quotient and the Short Quantitative Checklist for Autism in toddlers in 1,000 cases and 3,000 controls [corrected]. J Am Acad Child Adolesc Psychiatry. 2012; **51**(2): 202-12 e7.
38. Petrocchi S, Levante A, Lecciso F. Systematic Review of Level 1 and Level 2 Screening Tools for Autism Spectrum Disorders in Toddlers. Brain Sci. 2020; **10**(3).

Confidential: For Review Only

39. Williams J, Scott F, Stott C, et al. The CAST (Childhood Asperger Syndrome Test): test accuracy. Autism. 2005; **9**(1): 45-68.
40. Scott FJ, Baron-Cohen S, Bolton P, Brayne C. The CAST (Childhood Asperger Syndrome Test): preliminary development of a UK screen for mainstream primary-school-age children. Autism. 2002; **6**(1): 9-31.
41. World Health Organization. The ICD-10 Classification of Mental and Behavioural Disorders: Diagnostic Criteria for Research. Geneva: WHO; 1993.
42. Statistics. OfN. Self–coded version of the NS–SEC 2008 [Available from: http://www.ons.gov.uk/about–statistics/classifications/current/ns–sec/self–coded/index.html.
43. Lord C, Risi S, Lambrecht L, et al. The autism diagnostic observation schedule-generic: a standard measure of social and communication deficits associated with the spectrum of autism. J Autism Dev Disord. 2000; **30**(3): 205-23.
44. Lord C, Rutter M, Le Couteur A. Autism Diagnostic Interview-Revised: a revised version of a diagnostic interview for caregivers of individuals with possible pervasive developmental disorders. J Autism Dev Disord. 1994; **24**(5): 659-85.
45. Mullen EM. Mullen Scales of Early Learning manual. AGS ed. Circle Pines, MN: American Guidance Service; 1995. ix, 85 p. p.
46. Sparrow SS, Balla DA, Cicchetti DV. Vineland adaptive behavior scales: Survey forms manual: AGS Publ.; 2005.
47. StataCorp. Stata Statistical Software: Release 14. College Station, TX: StataCorp LP; 2015.
48. Pandey J, Verbalis A, Robins DL, et al. Screening for autism in older and younger toddlers with the Modified Checklist for Autism in Toddlers. Autism. 2008; **12**(5): 513-35.
49. Sturner R, Howard B, Bergmann P, et al. Accurate Autism Screening at the 18-Month Well-Child Visit Requires Different Strategies than at 24 Months. J Autism Dev Disord. 2017.
50. Yuen T, Penner M, Carter MT, Szatmari P, Ungar WJ. Assessing the accuracy of the Modified Checklist for Autism in Toddlers: a systematic review and meta-analysis. Dev Med Child Neurol. 2018.
51. Devescovi R, Monasta L, Bin M, et al. A Two-Stage Screening Approach with I-TC and Q-CHAT to Identify Toddlers at Risk for Autism Spectrum Disorder within the Italian Public Health System. Brain Sci. 2020; **10**(3).
52. Hyman SL, Levy SE, Myers SM, Council On Children With Disabilities SOD, Behavioral P. Identification, Evaluation, and Management of Children With Autism Spectrum Disorder. Pediatrics. 2020; **145**(1).

Confidential: For Review Only

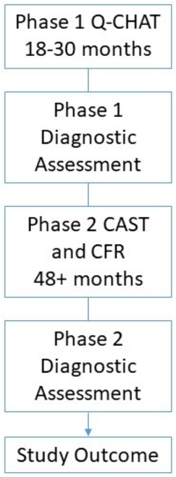

Figure 1 Study Design

190x338mm (96 x 96 DPI)

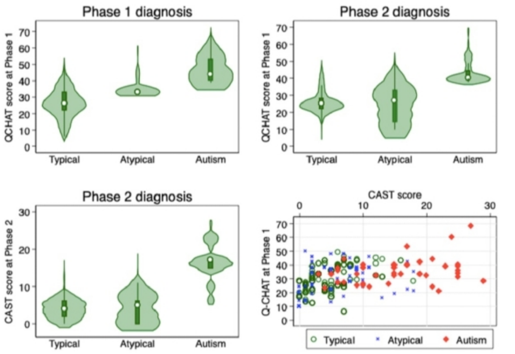

Figure 2 Characteristics and comparison of the Q-CHAT at Phases 1 and 2, and the CAST at Phase 2 (not weighted)

330x158mm (144 x 144 DPI)

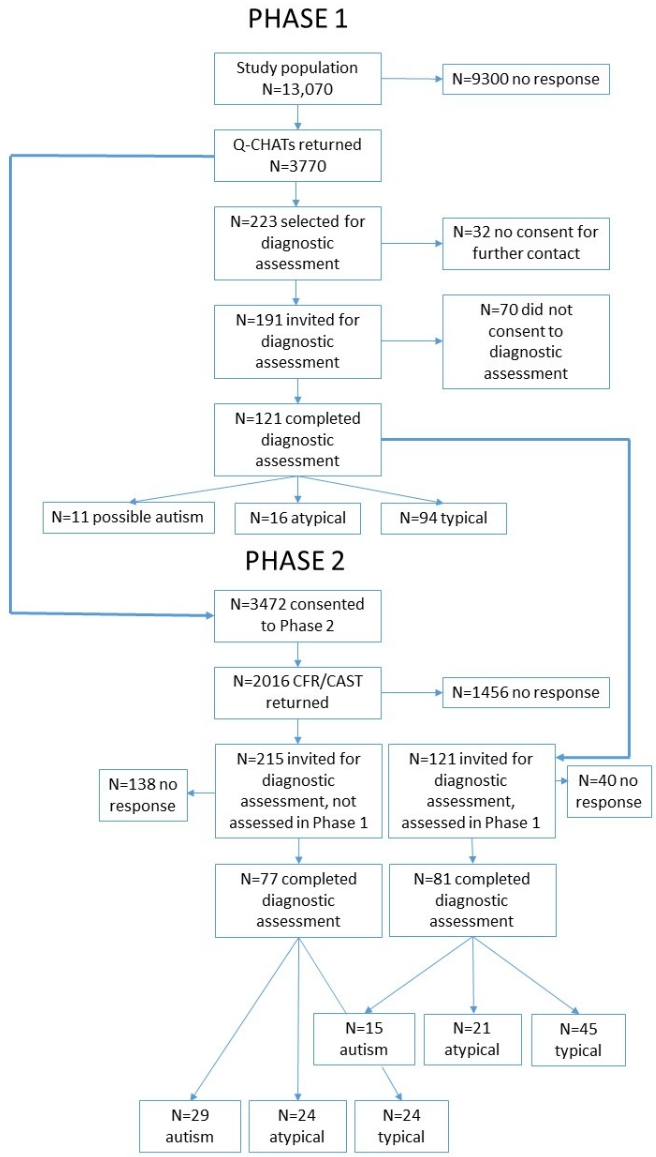

Figure 3 Participant study flow through screening and diagnostic assessments from Phase 1 to Phase 2

190x338mm (96 x 96 DPI)

Supplementary Appendix

Supplementary methods

Sensitivity analyses

Analysis was undertaken to maximise the sensitivity and specificity using a range of cut points across the Q-CHAT distribution. Additional cut points that also maximised sensitivity and specificity but had a larger number of false positives are provided. In addition a-priori a measure of parental concern taken at the same time as the screening test was included to investigate whether it improved the screening properties. Additionally, an analysis for initial non-response was undertaken to show the results were robust to the initial missing data.

Confidential: For Review Only

Supplementary Table 1: Study Characteristics

		Phase I				Phase II			
		Total		Assessment		Total		Assessment	
PCT	Luton	416	(11%)	22	(18%)	176	(9%)	19	(12%)
	Bedfordshire	1217	(33%)	39	(32%)	638	(32%)	55	(35%)
	Cambridgeshire	2018	(55%)	60	(50%)	1183	(59%)	84	(53%)
Child sex	Female	1759	(48%)	42	(35%)	972	(49%)	56	(35%)
	Male	1884	(52%)	79	(65%)	1025	(51%)	102	(65%)
	Unknown*	70		0		1		0	
Age child at Phase I	16–19 months	251	(7%)	15	(12%)	142	(7%)	13	(8%)
	20–24 months	1478	(41%)	49	(40%)	831	(42%)	69	(44%)
	25–29 months	1474	(40%)	46	(38%)	814	(41%)	66	(42%)
	30–34 months	440	(12%)	11	(9%)	210	(11%)	10	(6%)
	Unknown*	125		0		6		0	

* Children with unknown sex or age were excluded from the analysis and not included within the rest of the table
+ General Certificate of Secondary Education

Confidential: For Review Only

		Phase I				Phase II			
		Total		Assessment		Total		Assessment	
Mothers age at birth (Median, IQR)		32	[28,35]	31	[27,36]	33	[29,36]	32	[29,36]
Mothers education	None / <5 GCSE[+]	480	(13%)	25	(21%)	166	(8%)	19	(12%)
	5 GCSE	548	(15%)	21	(17%)	252	(13%)	24	(15%)
	2+ A levels	512	(14%)	11	(9%)	255	(13%)	15	(9%)
	Degree	1684	(45%)	48	(40%)	1068	(53%)	80	(51%)
	Unknown	546	(14%)	16	(13%)	267	(13%)	20	(13%)
Mother occupation	Not working	1545	(41%)	58	(48%)	736	(37%)	67	(42%)
	Student	86	(2%)	4	(3%)	40	(2%)	5	(3%)
	Employed	1044	(28%)	28	(23%)	592	(29%)	43	(27%)
	Self employed no employees	176	(5%)	5	(4%)	105	(5%)	9	(6%)
	Self employed with employees, employed supervisor	835	(22%)	22	(18%)	513	(26%)	31	(20%)
	Unknown	84	(2%)	4	(3%)	22	(1%)	3	(2%)
Mother	White	3236	(86%)	94	(78%)	1795	(89%)	141	(89%)
	Black (African or Caribbean)	103	(3%)	6	(5%)	36	(2%)	4	(3%)
	South East Asian	231	(5%)	13	(11%)	78	(4%)	7	(4%)
	Other / Unknown	200	(5%)	8	(7%)	99	(5%)	6	(4%)

Confidential: For Review Only

		Phase I				Phase II			
		Total		Assessment		Total		Assessment	
Fathers age at birth (Median, IQR)		34	[31,38]	34	[31,37]	35	[31,39]	35	[31,39]
Father education	None / <5 GCSE	613	(16%)	22	(18%)	268	(13%)	23	(15%)
	5 GCSE	477	(13%)	11	(9%)	223	(11%)	14	(9%)
	2+ A levels	439	(12%)	14	(12%)	232	(12%)	22	(14%)
	Degree	1547	(41%)	41	(34%)	983	(49%)	62	(39%)
	Unknown	694	(18%)	33	(27%)	302	(15%)	37	(23%)
Father occupation	Not working	166	(4%)	10	(8%)	61	(3%)	5	(3%)
	Student	40	(1%)	1	(1%)	14	(1%)	1	(1%)
	Employed	1153	(31%)	38	(31%)	634	(32%)	51	(32%)
	Self–employed – no employees	351	(9%)	11	(9%)	196	(10%)	20	(13%)
	Self–employed – with employees, employed supervisor	1729	(46%)	41	(34%)	994	(49%)	66	(42%)
	Unknown	331	(9%)	20	(17%)	109	(5%)	15	(9%)
Father ethnicity	White	3171	(84%)	96	(79%)	1780	(87%)	138	(87%)
	Black (African or Caribbean)	142	(4%)	8	(7%)	52	(3%)	6	(4%)
	South East Asian	237	(6%)	10	(8%)	78	(4%)	6	(4%)
	Other / Unknown	220	(6%)	7	(6%)	98	(5%)	8	(5%)

Confidential: For Review Only

Supplementary Figure 1: Phase 1 study flow

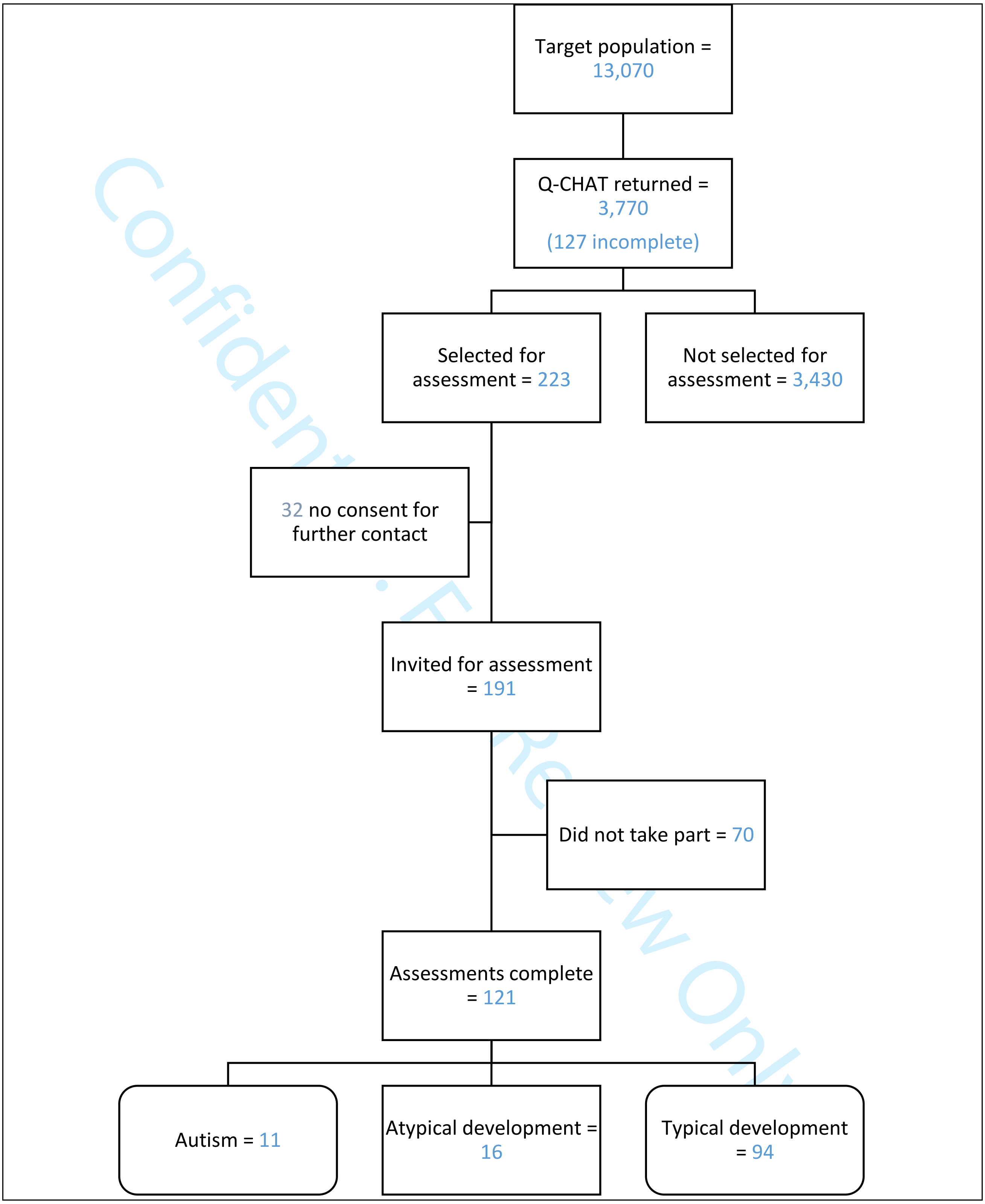

Confidential: For Review Only

Supplementary Figure 2: Distribution of Q–CHAT at first interview, with marks for screening groups

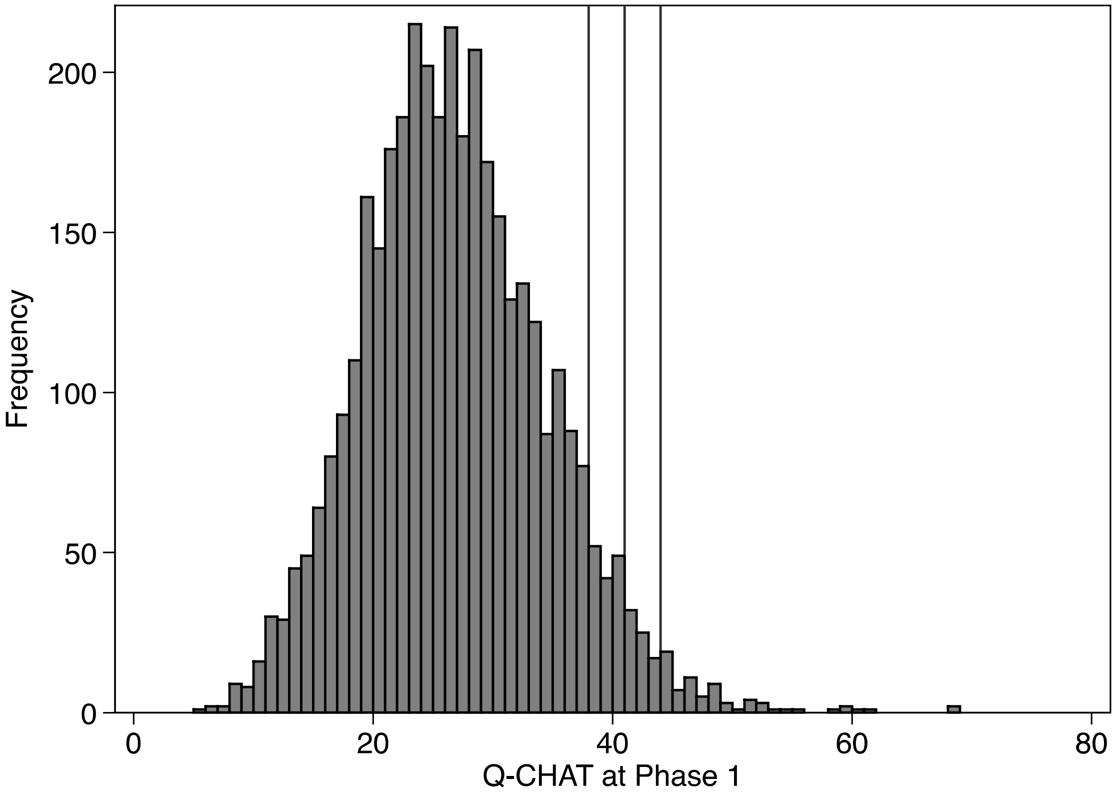

Review Only

Supplementary Figure 3: ROC curves for Q–CHAT and CAST on Phase 1 and Phase 2 outcomes for autism

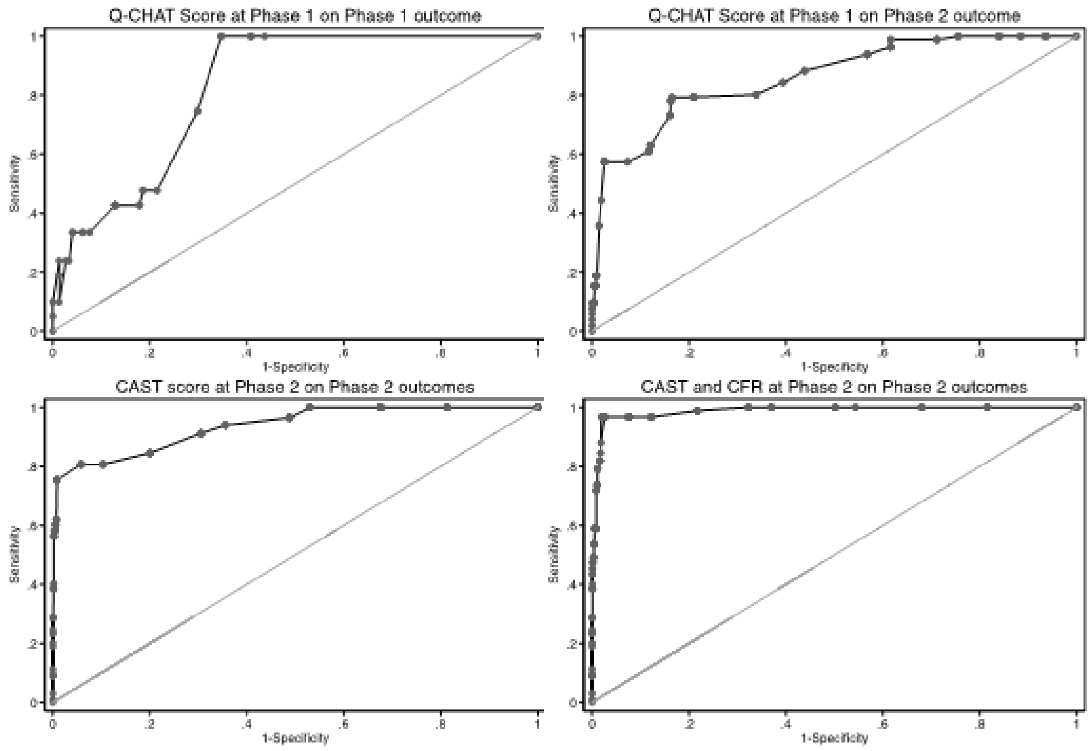

Supplementary Figure 4: Box-plot of the distribution of Q-CHAT scores split by both Phase 1 and Phase 2 diagnosis

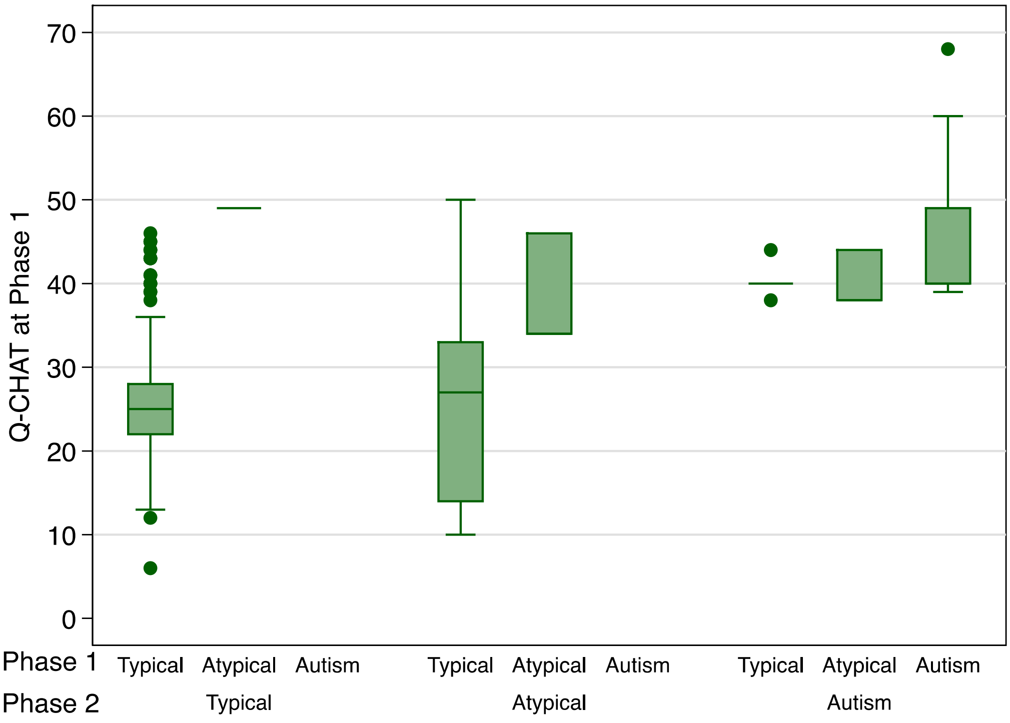

For Review Only
